# A Multidisciplinary Approach That Considers Occurrence, Geochemistry, Bioavailability, and Toxicity to Prioritize Critical Minerals for Environmental Research

**DOI:** 10.1021/acs.est.4c11211

**Published:** 2024-12-12

**Authors:** Sarah Jane O. White, Tyler J. Kane, Kate M. Campbell, Marie-Noële Croteau, Michael Iacchetta, Johanna M. Blake, Charles A. Cravotta, Bethany K. Kunz, Charles N. Alpers, Jill A. Jenkins, Katherine Walton-Day

**Affiliations:** † U.S. Geological Survey, Reston, Virginia 20192, United States; ‡ U.S. Geological Survey, Denver, Colorado 80225, United States; § U.S. Geological Survey, Columbia, Missouri 65201, United States; ⊥ U.S. Geological Survey, Moffett Field, California 94035, United States; || U.S. Geological Survey, New Cumberland, Pennsylvania 17070, United States; # Cravotta Geochemical Consulting, Bethel, Pennsylvania 19507, United States; □ U.S. Geological Survey, Sacramento, California 95819, United States; ○ U.S. Geological Survey, Albuquerque, New Mexico 87113, United States; △ U.S. Geological Survey, Lafayette, Lousiana 70506, United States

**Keywords:** Trace metals, technology critical elements, critical elements, environmental behavior, environmental health effects, sustainable resources

## Abstract

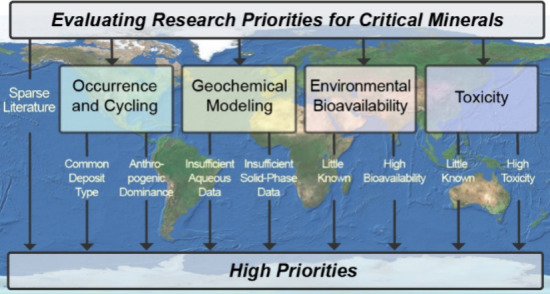

Critical minerals (or critical elements) are minerals or elements that are essential to global security and development and have supply chains vulnerable to disruption. In general, knowledge of the environmental behavior and health effects of critical elements is needed to support the development of safe and environmentally responsible supplies. This knowledge includes identifying potential consequences of increased critical element production and use, alternative critical element sources such as mine wastes, and adverse effects of critical elements on ecosystem condition and organismal health. Here we identify significant data gaps in the understanding of critical elements in surficial and aquatic environments, and the need, given the large number of commodities (50) identified on the 2022 critical minerals list for the United States, for an approach to prioritize them for study of their environmental fate and effects. We propose a multidisciplinary approach for this prioritization, considering measures of occurrence, geochemistry, bioavailability, and toxicity. We describe relatively easy-to-obtain metrics for each of these topic areas and demonstrate the utility of this integrated prioritization approach using indium and zinc as examples. This approach facilitates prioritizing research with a focus on those critical elements that are most mobile in the environment, bioavailable, toxic, or simply lacking data in these categories.

## Abstract 2

The environmental behavior and adverse effects of many critical elements are poorly understood. We propose an approach to prioritize critical element research.

## Background and Motivation

Critical minerals are essential to national and global security and prosperity, with supply chains vulnerable to disruption.^1^ The 2022 Critical Minerals list for the United States (U.S.)^2^ identifies 50 mineral commodities, of which rare-earth elements (REE), platinum group metals (PGM), and indium are most frequently identified as critical, globally.^3^ Other countries also have critical minerals lists, with overlapping and contrasting commodities to the United States.^3^ These commodities are associated with elements across the periodic table, and are used in a wide array of applications, including green energy, communications, defense, and consumer electronics. Yet for many of the critical minerals, only limited information is available about environmental occurrence, distribution, mobility, and potential adverse effects on ecosystem and biota health.^e.g.^^4−7^

Because the U.S. Critical Minerals list^2^ originates from a legislative mandate, “critical minerals” is a general term that is not strictly technical. The list includes a combination of elements and minerals; for this research prioritization effort, for tractability, we will discuss the primary element of interest associated with each mineral and will refer to them as “critical elements” (Table S1).

Increasing recovery and use of critical elements can enhance mobilization, environmental distribution, and exposures, with unexplored or unanticipated effects on biota and ecosystem health. For example, mineral extraction and processing often increase initial element concentrations by orders of magnitude in recovered concentrates or wastes, compounding the potential for harmful environmental release and exposure. However, the effects of increased exposures are difficult to predict because of substantial data gaps in geochemistry, bioavailability, and toxicity for many critical elements. At the same time, understanding the behavior of critical elements during processing and in wastes is the first step in improving their recovery and minimizing environmental impact. Given the large number of critical elements (∼50 on the most recent U.S. list (Table S1)) and the growing urgency to increase production, how do governments, regulators, scientists, and industries identify research priorities to help ensure that critical elements are recovered, refined, used, reused, and disposed in a sustainable, environmentally responsible manner?

A few studies have prioritized elements for research based on a single topic area (e.g., criticality, health impacts) using criteria such as geographic origin of current supplies, availability of alternative resources, anthropogenic disturbance of natural elemental cycles, and data availability for biomarkers and health impacts. For example, in determining which elements should be placed on the U.S. Critical Minerals list, Nassar et al.^1,8^ developed metrics for essentiality and vulnerability to supply chain disruption by which they could prioritize criticality. Likewise, Klee and Graedel,^9^ with further refinement from Sen and Peucker-Ehrenbrink,^10^ attempted to quantify human influence on the natural cycling of elements to understand and prioritize those elements whose cycling has been most perturbed. In a complementary report, Jenkins et al.^4^ summarized physicochemical and biochemical factors that affect the occurrence and bioavailability of selected critical elements, with emphasis on their potential toxicity in aquatic ecosystems.

**Figure 1:**
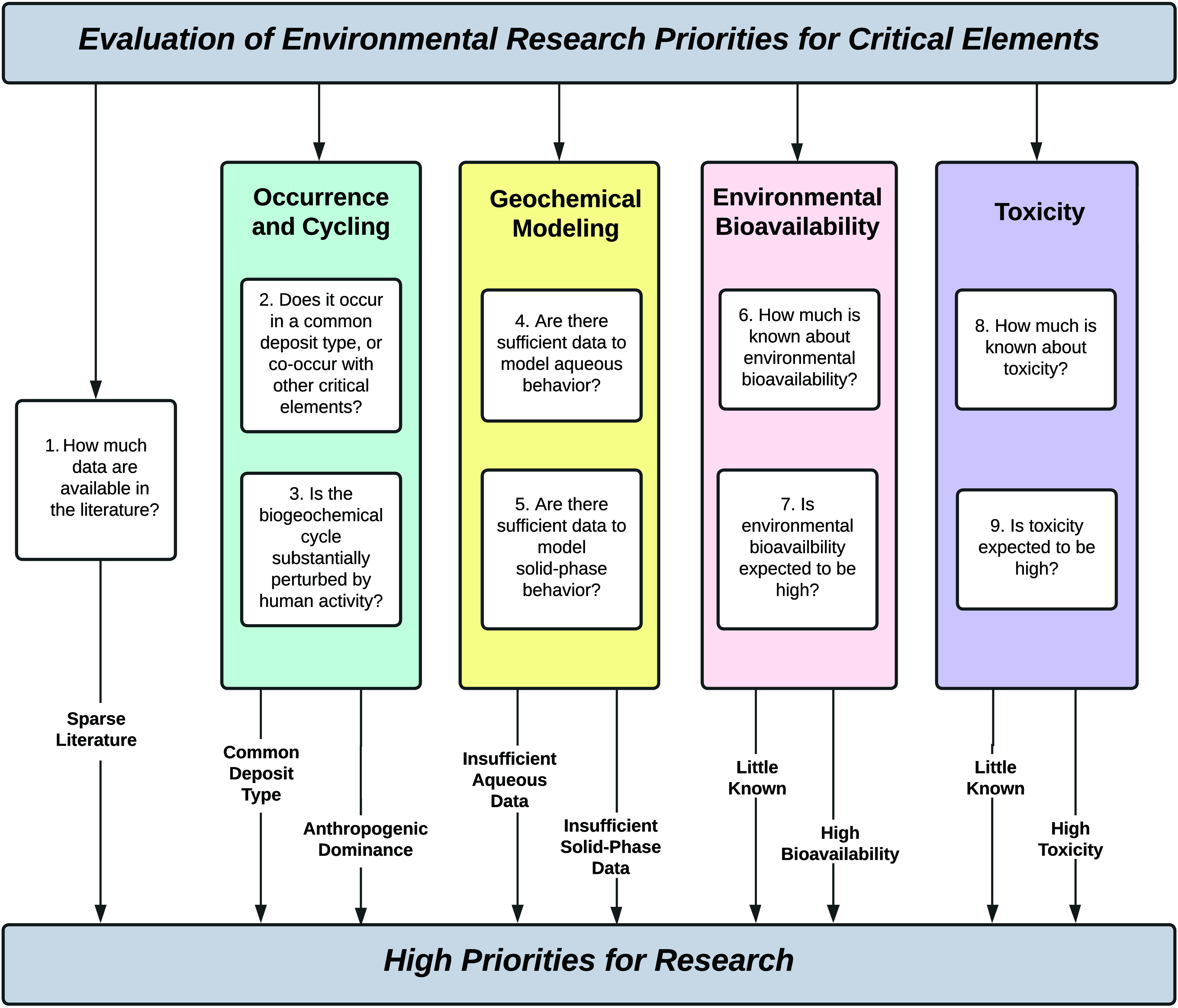
Multidisciplinary approach to prioritize critical elements for environmental research.

In this Perspective Piece we propose a multidisciplinary screening tool with multiple criteria to prioritize critical elements for study considering measures of available information in the literature, environmental occurrence and cycling, geochemistry, bioavailability, and toxicity (Figure 1). Given that 50 or more elements are considered critical for the United States (Table S1), using relatively easy-to-obtain metrics across multiple disciplines could enable a timely prioritization of research. The approach outlined here could be applied to critical minerals lists from other countries or industries. It builds on previous and existing efforts, including the persistence, bioaccumulation, and toxicity (PBT) characteristics central to environmental health risk assessment,^11^ extending the PBT approach to include occurrence and geochemical factors that affect metal mobility and behavior, and refining bioaccumulation to focus on direct measures of bioavailability (discussed below). Our approach also builds on efforts conducted in the 1980s to prioritize research on the effects of acid deposition on geochemical cycling and biological availability of trace elements.^12^ Aspects of the approaches are similar, though our goal is to identify metrics that do not rely on an exhaustive literature search for each element, nor expert opinion for interpretation of the metrics.

This Perspective Piece introduces an approach that could subsequently be used to prioritize environmental research for the full range of critical elements. Here we discuss: 1) suggested metrics for each of several topic areas; 2) an illustration of the metrics’ application, using two contrasting examples: a well-studied element, zinc (Zn), and a poorly studied, co-occurring element, indium (In); and 3) a simplified, binary scoring system to demonstrate the identification of research priorities. Such a multidisciplinary approach is key to building a holistic understanding of the fate, transport, and potential environmental health effects of critical elements.

## Disciplines to Consider and Possible Metrics

This multidisciplinary prioritization integrates knowledge from geochemistry, geology, biochemistry, physiology, ecology, and ecotoxicology. Toxicity to humans is not explicitly considered in the present discussion, but the work aligns with a One Health approach that assumes the connectedness of animal, human, and environmental health.^e.g.^^13^ For reviews of the human health effects of critical minerals, refer to Gwenzi et al., 2018,^14^ Jenkins et al., 2023,^4^ and Wang et al., 2024.^15^ Metrics in the current prioritization, based on the questions in Figure 1 and discussed in the following sections, include the number of review papers by element, element occurrence, anthropogenic influence on element cycling, and availability of data on geochemistry, bioavailability, and toxicity. These metrics can be evaluated individually, but we view them as integrated, and by evaluating them as a whole, biases introduced by any individual metric may be attenuated. For the purposes of this Perspective Piece, a simplified, cumulative binary scoring system, where an element is assigned a 0 or 1 for each metric (0 meaning low priority, 1 meaning high) allows both a comparison of relative priority among elements and prioritization of topic areas for individual elements. As discussed below, zinc is a critical element with a moderate number of review papers available, and an abundance of data showing high toxicity.^16,17^ Zinc is essential to a number of biological functions, and its bioavailability is relatively high.^18^ We contrast zinc with indium, an element with sparse data for each of the topic areas considered, a biogeochemical cycle dominated by human activities,^9^ and the potential for moderate toxicity according to the limited available data.^16,19^ The behavior and impacts of indium are highly uncertain, and research to address data gaps could be impactful. Although research on zinc is still warranted, particularly where gaps have been identified, new research on zinc may be less of a priority than that for other critical elements with larger data gaps.

## Review Papers by Element

One metric to represent the body of knowledge of an element is the total number of review papers published (Question 1 (Q1) in Figure 1). Review papers provide a tractable quantity (as opposed to total papers published, for example), and represent both interest in, and understanding of, the occurrence and behavior of a particular element. This metric can be used to indicate the types of data available, as well as information on relevant data gaps.

For each critical element, a standardized search was performed using the Web of Science Core Collection database. Search queries returned only publications classified as review articles where the critical element name was included in the publication title; additional keywords (“geochemistry,” “toxicity,” “resource,” “environment,” “bioavailability,” “mining,” and “thermodynamic”) were examined individually. Further detail and a full list of references is provided in the Supporting Information (SI).

**Figure 2:**
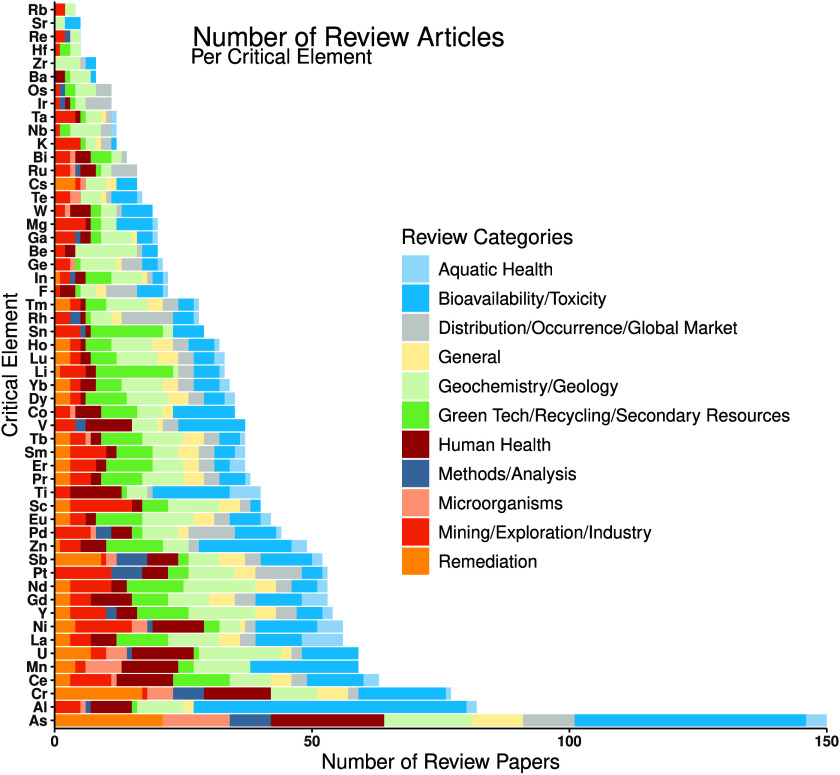
Review papers published through March 2023 for each of the critical elements listed on the 2018 and 2022 U.S. Critical Minerals lists^2,20^ (Table S1), arranged by category defined by this study. A complete list of references for this figure are in the SI, obtained from the Web of Science Core Collection database.

**Figure 3:**
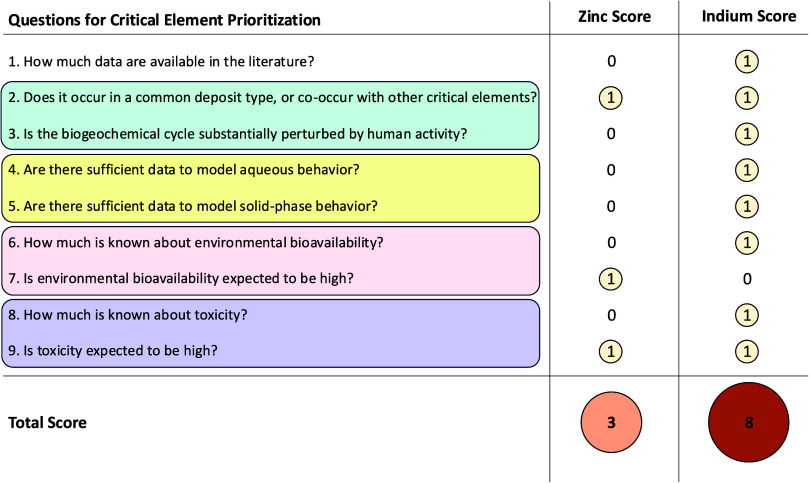
Prioritization scores for zinc and indium using a binary system based on the questions posed in Figure 1. A score of 1 indicates that the answer to the question elevates the priority of the critical element for future studies. A score of 0 indicates that the answer to the question reduces the priority of the element for future studies. Possible total scores range from 0 (lowest priority) to 9 (highest priority).

More than 1,200 publications published through March 2023 were categorized into 11 review categories (Figure 2 and SI References). Of the critical elements considered, zinc had the 14th highest number of reviews (49), and indium the 34th highest (22). This ranking would elevate indium’s priority for study (Figure 3). There are more zinc reviews focused on “Bioavailability/Toxicity” than any other category, whereas the “Geochemistry/Geology” category was dominant for indium. Both elements are missing representation in the “Microorganisms” category, which encompasses reviews focused predominantly on microbial-element interactions. The distribution of review articles across the critical elements likely has been driven by factors such as known human and environmental health effects, technological and industrial use, as well as historic economic value.

## Occurrence and Cycling

Geologic formations and mineral deposits control where specific resources and co-occurring elements are naturally found. For example, indium is primarily produced as a byproduct of mining the mineral sphalerite (ZnS)^21^ due to its substitution into the ZnS structure; therefore, the mining, processing, and environmental behaviors of these two elements are linked. The average crustal abundance of indium (0.056 mg/kg or 0.49 μmol/kg) is much less than that of zinc (70 mg/kg or 1.1 mmol/kg),^22,23^ and the mean concentration of indium (3.22 x10^–5^ μg/L or 0.28 pM) in surface waters is much less than that of zinc (0.6 μg/L or 9.2 nM).^24^ Data are available for stream sediment and suspended sediment concentrations in world rivers for zinc but not for indium.^25,26^ Both elements have soil chemistry data available.^27^ In prioritizing critical elements for research, it can be advantageous to consider the distribution of deposit types and (or) mineral systems in which the element occurs^e.g.^^28−30^ (Q2 in Figure 1); those elements that are associated with more widespread deposits have ample opportunity for mobilization and exposure through mining and mineral processing, and are therefore considered higher priority than those that are rare or have limited spatial distribution. Because indium and zinc co-occur in sphalerite, which is common, both are considered high priority for research in this respect.

Perturbing the natural cycling of an element may lead to detrimental environmental health effects (Q3 in Figure 1). For example, emissions of mercury to the atmosphere from coal combustion and widespread small-scale mining are a global-scale problem whereby deposition and biomagnification through food chains impact humans and wildlife worldwide.^31^ Researchers have proposed that anthropogenic influences on natural biogeochemical cycling can be used to identify an element for research and monitoring, and several have attempted to quantify cycling for many of the elements of the periodic table.^e.g.^^9,10^ Using the quantification by Klee & Graedel^9^ as a metric for anthropogenic influence, we can bin anthropogenic disturbance into low (0–49% of the quantified fluxes are anthropogenic) and high (50–100% anthropogenic). Zinc falls into the low bin (47%), suggesting a low priority for further research. In contrast, indium has high anthropogenic disturbance (90%), which may lead to environmental health problems associated with increased exposure. Thus, further research on indium may be impactful (Figure 3).

## Geochemistry

Geochemistry provides a framework to understand and explain quantitative interactions of the critical elements in terrestrial and aquatic environments. Chemical properties such as environmentally relevant oxidation state(s) and common aqueous ionic form(s) are fundamental to geochemical behavior,^22,32^ and can impact an element’s bioavailability or toxicity; identifying whether a critical element is an essential nutrient or microbial growth substrate can help link geochemical mechanisms to an element’s bioavailability or toxicity. Zinc has one environmentally relevant oxidation state (+2) whereas indium has two (+1 and +3), providing an opportunity for redox behavior. Both elements are commonly found in aqueous systems as cations, which is important because ionic form influences interactions with particles and organisms. Zinc is considered an essential nutrient, whereas indium is not.

Geochemical modeling is widely used to understand element mobility and speciation in natural and contaminated waters. Evaluating available aqueous complexation and mineral solubility constants used in geochemical modeling can help identify gaps in understanding mechanisms such as mineral precipitation/dissolution, adsorption/desorption, or aqueous speciation that could affect distribution, bioavailability, and toxicity.^32,33^ Here, we suggest that availability of data in common thermodynamic database files (TDF) can be used as a metric to prioritize critical element research. For the current example, we compared five relatively comprehensive TDF, which were built for varying purposes and previously compiled for use with PHREEQC^34,35^ (see SI for further detail), to assess availability of constants needed to determine: (1) mineral solubility and associated limitations on dissolved concentrations; and (2) inorganic aqueous speciation (distribution of dissolved components among free ions, ion pairs, and complexes). Not considered here, but ultimately of importance, are surface reactions (adsorption) and metal–organic interactions.

The behavior of zinc in the environment is well documented,^18,36^ reflected by the abundance of thermodynamic data for an array of ligands for both aqueous speciation and mineral precipitation^37,38^ (Figure S1; Q4&5 in Figure 1). Zinc is therefore not a high priority for study based on geochemical modeling considerations (Q4&5 in Figure 3). In contrast, indium has only a few reported equilibrium constants for selected aqueous species (Q4) and only a single solubility product constant for oxidation of metallic In to aqueous In^3+^ (Q5; Figure S1). Additional data on indium have been reviewed by Wood and Samson^39^ and Tuck^40^ that may supplement the TDF, but the available data are not comprehensive. Thus, indium is a high priority for geochemical research based on this approach (Q4&5 in Figure 3).

To further develop an optimal metric for geochemical prioritization, modeling the behavior of each element in a simple representative natural water can be used to identify issues not highlighted by a simple counting of constants, including inconsistencies between databases. Additionally, an element with a greater chemical tendency to form complexes or compounds will result in a higher number of thermodynamic constants. Another benefit of modeling element behavior is to predict, for example, the most probable exposure pathway(s); waterborne or dietary uptake routes may be predicted based on the element’s modeled presence in aqueous and solid phases. In the case of zinc, the five TDF are consistent and show that waterborne exposure is expected for organisms in a simulated circumneutral surface water (see SI for description of modeling). Indium lacks sufficient data to predict a likely exposure pathway.

## Bioavailability

Bioavailability is central to determining environmental effects of critical elements as it directly links exposure to uptake by living organisms. Definitions vary for bioavailability across disciplines. Here we define bioavailability as the fraction of metal internalized or adsorbed by an organism.^41−43^ By controlling the extent of bioaccumulation (the accumulation of contaminants in biological tissues over time), bioavailability critically influences toxicity because bioaccumulated metals, specifically metabolically available elements, are precursors to adverse effects.^44^ Water chemistry (e.g., pH, alkalinity, dissolved organic matter) is a critical driver of metal bioavailability and toxicity.^33,45^ Geochemical models have been central to identifying the chemical species that best predict biological responses from dissolved exposures.^33,46^ But the thermodynamic data needed for modeling are lacking for many critical elements. Guidelines for environmental assessments often assume 100% bioavailability for contaminants,^47^ but bioavailability varies greatly among elements^48,49^ and geochemical conditions.^33,45^ Bioavailability is also a more direct measure of potential element assault to organisms than bioaccumulation, which is the result of complex physiological and adaptative processes, including uptake, loss, sequestration, and detoxification.^49^

In addition to direct measurements of bioaccumulated concentrations, bioavailability proxies such as influx rates and assimilation efficiencies (for dietary exposures) are determined in *in vivo* studies using model species and often tracer techniques.^e.g.^^50−52^ Although a great deal of mechanistic data on metal bioavailability in aquatic systems has been generated over the last three decades,^45,49,50,53−55^ little is known about the bioavailability of most critical elements, especially when they occur in solid phases, because dietary bioavailability experiments are complex.^56^ Furthermore, uptake pathways and cellular transport mechanisms are largely unknown for most critical elements, especially those that are not nutritionally essential.^4,7^

In the context of our prioritization framework, high priority is given to critical elements whose environmental bioavailability is expected to be high, and to those with little available information (Q6&7 in Figure 1). For example, elements with known essential biological roles (e.g., Co, Ni, Zn), or elements that can substitute for an essential element because of similar chemical properties (e.g., Sr for Ca, Cs for K or Na) would be expected to be highly bioavailable and thus prioritized. For this reason, zinc is given high priority (Q7); however, its bioavailability is relatively well-studied, meaning that further studies may be less impactful than for poorly studied elements (Q6, Figure 3). Indium is the opposite–it is low priority based on expected low bioavailability^57^ (Q7; i.e., indium has no known biological role and is not known to substitute for essential elements), but high priority (Q6) based on lack of information (e.g., none of the review studies on indium addresses bioavailability (Figure 2)). Ultimately, assessments of the bioavailability of critical elements by experimentally determining bioavailability proxies can help to fill this knowledge gap.

## Toxicity

Quantifying the toxicity of an element is crucial in determining the potential effects of increased environmental exposures. Elevated metal exposures may cause pervasive, damaging effects on wildlife and humans. For example, lead is a well-studied element which induces neurological, respiratory, urinary, and cardiovascular disorders upon chronic exposure to humans and wildlife.^58^ Understanding the toxicity of increased levels of lead in the bloodstream has resulted in regulation to reduce use of lead in the United States to protect humans^e.g.^^59,60^ and culturally important or imperiled wildlife.^61^

Toxicity is dependent on a substance’s chemical structure, bioavailability in the environment, and the affected organism’s ability to eliminate and detoxify the substance.^e.g.^^22^ These geochemical and physiological properties differ depending on the element and the organism exposed. In a prioritization based on toxicity, higher priority is given to critical elements that have higher relative toxicity and subsequently greater potential environmental effect (Q9 in Figure 1). Also, high priority is given to critical elements that lack available toxicity information because there is a greater likelihood for misinformed decisions when the understanding of an element’s toxicity is low (Q8 in Figure 1).

To better understand critical element toxicity, we surveyed existing data from the U.S. Environmental Protection Agency (EPA) ECOTOXicology Knowledgebase (ECOTOX).^16,62^ This is a curated database of ecologically relevant toxicity tests used to support environmental research and risk assessments.^16^ Using this database allowed comparison of standardized toxicity end points across all available critical elements. Data for many elements were limited. Toxicity metrics used in the current approach were restricted to the most common type of data reported–acute toxicity studies using aquatic arthropods and fish as test organisms. Once the records were organized and summarized, we designated thresholds for grouping relative toxicity of the critical elements based on the “Ecotoxicity Categories” for aquatic organisms in acute toxicity experiments, originally developed by the EPA for pesticides.

Zinc is a highly bioavailable critical element with an abundance of information available on its environmental toxicity. In ECOTOX,^16^ there are >400 total records on the toxicity of zinc to aquatic arthropods and fish (Table S2). The median LC_50_ (lethal concentration for 50% of the sampled population) value for all records available for aquatic arthropods in acute exposures falls in the range 0.1 to 1.0 mg/L (or 1.5 to 15 μM) for zinc (Table S2). This categorizes zinc as a highly toxic element and therefore a high priority for future environmental health studies (Q9, Figure 3). In contrast, indium has very few data on environmental toxicity. Indium has only two available records in ECOTOX, both of which were conducted using the same organism in acute exposures (Table S2). The median LC_50_ of these two records is 2.1 mg/L (18 μM), considered moderately toxic (Table S2). Although indium may have lower toxicity in acute aquatic exposures than zinc, indium’s priority is elevated due to an inability to adequately predict environmental toxicity using existing data (Figure 3). Many critical elements currently lack available toxicity information, which highlights the opportunity to fill in these knowledge gaps by conducting experiments for the most understudied elements.

## Discussion and Conclusions

Critical element research is of high importance globally as researchers and decision makers work to identify primary and alternative sources, and to understand the environmental behavior and risk from elements mobilized from new and legacy mining, and industrial use. Given the large number of critical elements and the sparse data available for many, a prioritization approach for future research can help focus attention on the most pressing questions. In some cases, elements may be assigned higher priority because of insufficient data to understand geochemical, biological, and toxic characteristics. Available data can help focus attention on elements that are likely to be widely distributed, available in an easily transportable or more bioavailable form, or exhibit higher toxicity.

A multidisciplinary approach can help to prioritize critical minerals for research because of the variety of research goals to address and the inherent interdependence of the topic areas involved. For example, geochemical properties such as solubility and tendency to form aqueous complexes with specific ligands inform how an element partitions during mineral processing, which in turn informs its environmental fate, including presence in specific wastes. Along with climatic conditions, geochemical parameters dictate form and speciation in solid and aqueous media, how host minerals and speciation change during weathering of mine wastes, the likelihood of mobilization, and probable exposure pathways (e.g., aqueous or dietary). Bioavailability and toxicity both depend on the geochemical form, and toxicity is closely linked to bioavailability because bioavailability influences the extent of element bioaccumulation.

Whatever prioritization approach is used, it is important that the questions framed include geochemical, biological, and toxicological considerations, and that relatively easy-to-obtain metrics are chosen. The approach presented here is not a complete critical analysis of each element in each topic area (Figure 1); instead, it is a tractable way of prioritizing elements for study that could spawn more in-depth reviews and specific research.

The utility of this approach is demonstrated by comparing results for zinc and indium (Figure 3). Using the approach, zinc is identified as a high priority in three topic areas: occurrence in common deposit types, high expected bioavailability, and high expected toxicity. Indium, on the other hand, is identified as a high priority in eight categories: few review papers available; natural cycling perturbed by human activity; occurrence in common deposit types; sparse data to understand aqueous phase behavior, solid phase behavior, bioavailability, and toxicity; and expected moderate relative toxicity. From this comparison, it is clear that filling data gaps for indium in any of these categories could advance knowledge of that critical element and thus indium would score as an overall higher environmental research priority than zinc. In addition to overall priority, this approach can identify specific topic areas that warrant focus for future research, including for elements that may be of lower overall priority. Examples include: 1) for indium, bioavailability and toxicity studies could benefit from first performing geochemical studies to identify the most relevant ionic species expected, and identifying whether waterborne or dietary exposure (or both) are relevant; and 2) for zinc, studies focusing on conditions favoring high mobilization or potential for exposure may be warranted, given zinc’s well-known high bioavailability and high toxicity. Ultimately, developing a practical, easily applied method for prioritization of critical elements for study can guide research on a wide variety of questions across disciplines and enable a more integrated approach to critical minerals science.

## Supporting Information Available

Full list of critical minerals and associated critical elements; full list of 1217 references used to create Figure 2; additional details of figure creation; additional description and figures for geochemical evaluation; comparison of toxicity data for zinc and indium (PDF)
