# Supplementary material for: A Multidisciplinary Approach That Considers Occurrence, Geochemistry, Bioavailability, and Toxicity to Prioritize Critical Minerals for Environmental Research
Source: Environ Sci Technol. 2024 Dec 12;58(51):22519–27. doi: 10.1021/acs.est.4c11211 (PMC11673130; doi:10.1021/acs.est.4c11211)
Supplement: Supplementary file 1 — es4c11211_si_001.pdf [file es4c11211_si_001.pdf]

A multidisciplinary approach that considers occurrence, geochemistry, bioavailability, and toxicity to prioritize critical minerals for environmental research

Sarah Jane O. White[1,*], Tyler J. Kane[1], Kate M. Campbell[1], Marie-Noële Croteau[1], Michael Iacchetta[1], Johanna M. Blake[1], Charles A. Cravotta III[1,2], Bethany K. Kunz[1], Charles N. Alpers[1], Jill A. Jenkins[1], Katherine Walton-Day[1]

[1]U.S. Geological Survey; [2]Cravotta Geochemical Consulting
[*]Corresponding Author, sjowhite@usgs.gov

Supporting Information

83 pages, 1 figure, 2 tables

Table S1. Compilation of the 2018 and 2022 U.S. Critical Mineral Lists[1, 2] and the associated critical elements used in this work. The "*" indicates critical minerals not considered in this study because they are primarily gaseous (He), the elemental equivalent is ubiquitous (C for graphite), or the element exists in vanishingly small concentrations naturally and is therefore considered to be a synthetically produced element (Pm). A dash (-) indicates the mineral was not included in that year's list.

2018 List	2022 List	Critical Element list (this paper)
Bauxite	Aluminum	Al
Antimony	Antimony	Sb
Arsenic	Arsenic	As
Barite	Barite	Ba
Beryllium	Beryllium	Be
Bismuth	Bismuth	Bi
Cerium	Cerium	Ce
Cesium	Cesium	Cs
Chromium	Chromium	Cr
Cobalt	Cobalt	Co
Dysprosium	Dysprosium	Dy
Erbium	Erbium	Er
Europium	Europium	Eu
Fluorspar	Fluorspar	F
Gadolinium	Gadolinium	Gd
Gallium	Gallium	Ga
Germanium	Germanium	Ge
Graphite	Graphite	C*
Hafnium	Hafnium	Hf
Helium	-	He*
Holmium	Holmium	Ho
Indium	Indium	In
Iridium	Iridium	Ir
Lanthanum	Lanthanum	La
Lithium	Lithium	Li
Lutetium	Lutetium	Lu
Magnesium	Magnesium	Mg
Manganese	Manganese	Mn
Neodymium	Neodymium	Nd
-	Nickel	Ni
Niobium	Niobium	Nb
Osmium	-	Os
Palladium	Palladium	Pd
Platinum	Platinum	Pt

Potash	-	K
Praseodymium	Praseodymium	Pr
Promethium	-	Pm*
Rhenium	-	Re
Rhodium	Rhodium	Rh
Rubidium	Rubidium	Rb
Ruthenium	Ruthenium	Ru
Samarium	Samarium	Sm
Scandium	Scandium	Sc
Strontium	-	Sr
Tantalum	Tantalum	Ta
Tellurium	Tellurium	Te
Terbium	Terbium	Tb
Thulium	Thulium	Tm
Tin	Tin	Sn
Titanium	Titanium	Ti
Tungsten	Tungsten	W
Uranium	-	U
Vanadium	Vanadium	V
Ytterbium	Ytterbium	Yb
Yttrium	Yttrium	Y
-	Zinc	Zn
Zirconium	Zirconium	Zr

Additional description of collection of review papers

For each critical element, a standardized search was performed using the Web of Science Core Collection database. Search queries returned only publications classified as review articles where the critical element name was included in the publication title; additional keywords ("geochemistry," "toxicity," "resource," "environment," "bioavailability," "mining," and "thermodynamic") were searched one by one. Element groups such as rare-earth elements (REE) and platinum group metals (PGM) were searched, as well as individual elements in these two groups. Review articles addressing element groups were assigned to individual elements based on the specific elements addressed within the article. For example, if an article title contained "rare earth elements" but focused only on cerium, lanthanum, and neodymium within the body of the article, it would be included in the article count for each of the three elements addressed. Articles presenting new data from field or laboratory studies or documenting new engineering, technological, or medical applications were excluded as they did not meet our definition of "review."

Additional description of geochemical thermodynamic database evaluation

In some cases, thermodynamic data are incomplete or unavailable for the critical element, limiting possibilities for geochemical modeling. To identify data gaps for all of the critical elements, we reviewed equilibrium reactions for aqueous speciation and mineral solubility in five published thermodynamic database files formatted for use in PHREEQC[3]: wateq4f[4], llnl[5], PRODATA[6], Thermochimie[7], and Thermoddem[8, 9]. The first two databases were provided with PHREEQC[e.g. 3, 10], whereas the other three, in required format and including various additional elements, were obtained separately. The PhreePlot "list species app" [11] was used to help organize the databases for review. Summary results for the available data on zinc and indium are indicated in Figure S3, where counts of equilibrium speciation reactions by element and corresponding ligands are displayed. On the basis of this review, five elements (iridium, osmium, rhenium, tantalum, and tellurium) were identified as lacking any aqueous speciation data in an existing PHREEQC database.

Figure S1. Comparison of available equilibrium speciation reactions for indium and zinc across five thermodynamic databases. For each element, equilibrium speciation reactions describing aqueous species formation and solid phase dissolution are separated for comparison. The total number of entries in each category are further divided into major anion groups defined by the counter ions necessary for the reaction to occur. When more than one anion group is represented in a given reaction, the reaction is counted multiple times, once for each anion group present. For example, $Zn_2(OH)_3Cl$ would be counted twice, once for the OH^- counter ion and once for the Cl^- counter ion. This accounts for 24 of the 163 total solid phase entries and 6 of the 125 total aqueous species entries for zinc. There were no reactions with multiple counter ions for indium.

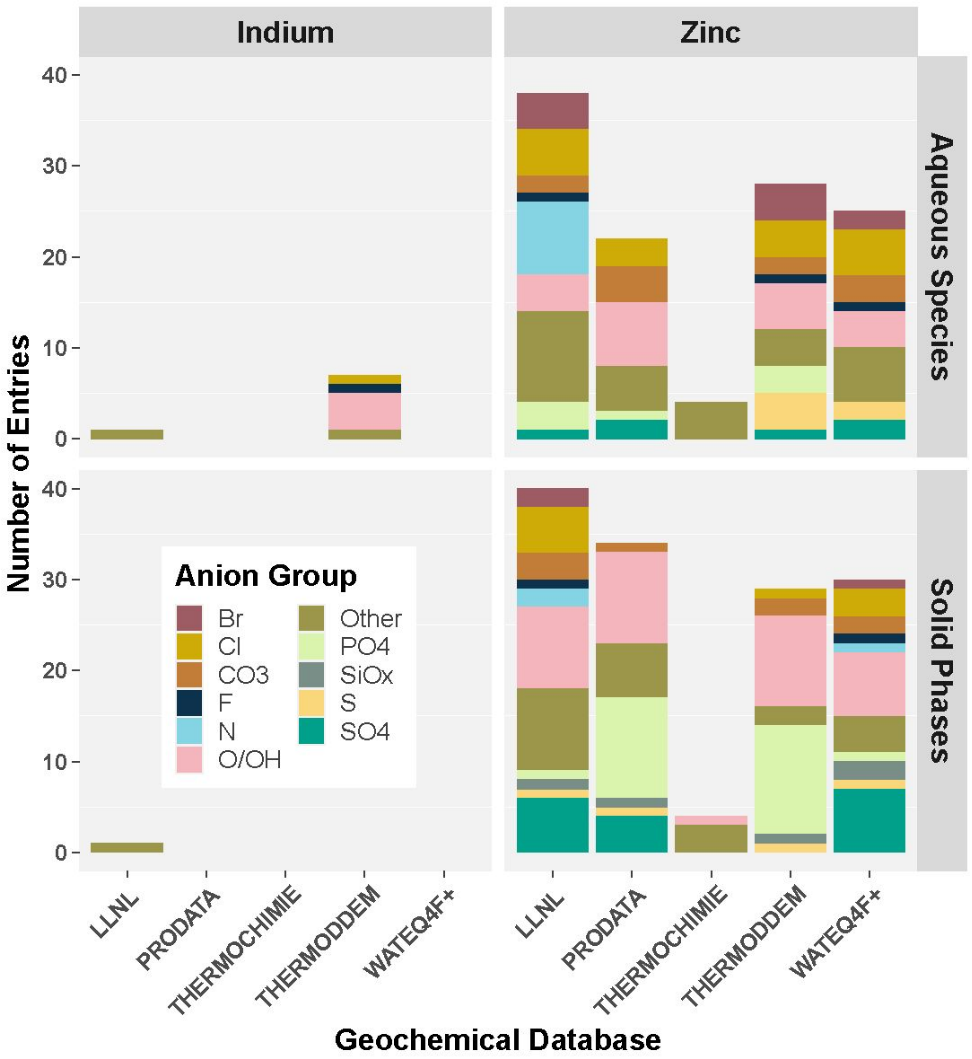

Additional modeling for zinc and indium was conducted with PHREEQC to identify issues not highlighted by the above counting of constants and to reveal what is known about the behavior of zinc and indium in a moderately hard freshwater system. Modeling was done by simulating aqueous speciation of a circumneutral natural surface water commonly used for toxicity and bioavailability testing (moderately hard synthetic water (MOD) [12]). In this series of simulations, 1μM of each critical element was added to simulated MOD water at pH 7.50 and in equilibrium with atmospheric CO_2, and the number of aqueous species and mineral phases were counted. The same concentration of each element was added for independent simulations, so that results could be compared directly across databases and between elements. If any phases were oversaturated, the least soluble phase was allowed to precipitate to determine order-of-magnitude solubility control of the aqueous concentration by that pure mineral phase. We recognize that aqueous concentrations may also be controlled by the formation of solid solutions and/or adsorption onto other mineral phases or organic materials, but these processes were beyond the scope of the present modeling exercise. Predominant dietary exposure was defined as a decrease in aqueous concentrations by at least one order of magnitude upon precipitation of the least soluble solid phase; if this condition was not met then the waterborne pathway was

defined as the dominant pathway. In the case of zinc, the five databases are consistent and show that waterborne exposure is expected for organisms in a simulated circumneutral surface water. Indium lacks sufficient data to predict a likely exposure pathway.

Additional toxicity information for zinc and indium

Table S2. Comparison of available toxicity information for zinc and indium in ECOTOX[13, 14]. Available publications include literature on the toxicity of the corresponding critical element to both humans and other living organisms. Available toxicity records only include records for the five most frequently used endpoints (LC_{50}, EC_{50}, EC_{10}, NOEC, and LOEC) across the critical elements. NA = not available.

Comparison of Toxicity Metrics for Zinc and Indium		
	Zn	In
Available Publications (*ECOTOX database*)	507	1
Total Available Toxicity Records (*Arthropod and Fish Acute Aquatic Exposures*)	475	2
Median LC_{50} in mg/L (*Arthropod-acute exposures*)	0.174 mg/L (2.7×10^{-3} mM)	2.075 mg/L (1.8×10^{-2} mM)
Median LC_{50} in mg/L (*Fish-acute exposures*)	0.5 mg/L	NA
Toxicity Ranking	Highly Toxic	Moderately Toxic

Disclaimer

Any use of trade, firm, or product names is for descriptive purposes only and does not imply endorsement by the U.S. Government.

References (excluding Figure 2, which follow)

(1) Interior Department Office of the Secretary. Final List of Critical Minerals 2018 [notice]. 83(97)23295-23296, F.R. Doc. 2018-10667. Federal Register: May 18, 2018.
(2) U.S. Geological Survey. 2022 Final List of Critical Minerals [notice]. 87(37)10381-10382, F.R. Doc. 2022-04027. Federal Register: February 24, 2022.
(3) Parkhurst, D. L.; Appelo, C. Description of input and examples for PHREEQC version 3—a computer program for speciation, batch-reaction, one-dimensional transport, and inverse geochemical calculations. *US Geological Survey Techniques and Methods* **2013**, *6* (A43), 497.
(4) Ball, J. W.; Nordstrom, D. K. User's manual for WATEQ4F, with revised thermodynamic data base and test cases for calculating speciation of major, trace, and redox elements in natural waters. *US Geological Survey* **1991**.

(5) Daveler, S. A.; Wolery, T. J. *EQPT, a data file preprocessor for the EQ3/6 software package: User's guide and related documentation (Version 7.0). UCRL-MA—110662-Pt2*; Lawrence Livermore National Laboratory, Livermore, California, 1992.
(6) Reiller, P. E.; Descostes, M. Development and application of the thermodynamic database PRODATA dedicated to the monitoring of mining activities from exploration to remediation. *Chemosphere* **2020**, *251*, 126301. DOI: 10.1016/j.chemosphere.2020.126301.
(7) Duro, L.; Grivé, M.; Giffaut, E. ThermoChimie, the ANDRA Thermodynamic Database. *MRS Online Proceedings Library* **2012**, *1475* (1), 589-592. DOI: 10.1557/opl.2012.637.
(8) Blanc, P.; Lassin, A.; Piantone, P.; Azaroual, M.; Jacquemet, N.; Fabbri, A.; Gaucher, E. C. Thermoddem: A geochemical database focused on low temperature water/rock interactions and waste materials. *Appl Geochem* **2012**, *27* (10), 2107-2116. DOI: 10.1016/j.apgeochem.2012.06.002.
(9) Blanc, P. *Thermoddem: Update for the 2017 version. Report BRGM/RP-66811-FR.*; 2017.
(10) Lu, P.; Zhang, G.; Apps, J.; Zhu, C. Comparison of thermodynamic data files for PHREEQC. *Earth-Sci Rev* **2022**, *225*, 103888.
(11) Kinniburgh, D. G.; Cooper, D. M. *PhreePlot Guide, Creating graphical output with PHREEQC*; http://www.phreeplot.org, 2011.
(12) U.S. Environmental Protection Agency. Methods for Measuring the Acute Toxicity of Effluents and Receiving Waters to Freshwater and Marine Organisms, EPA-821-R-02. Washington, DC, 2002.
(13) Olker, J. H.; Elonen, C. M.; Pilli, A.; Anderson, A.; Kinziger, B.; Erickson, S.; Skopinski, M.; Pomplun, A.; LaLone, C. A.; Russom, C. L.; Hoff, D. The ECOTOXicology Knowledgebase: A curated database of ecologically relevant toxicity tests to support environmental research and risk assessment. *Environ Toxicol Chem* **2022**, *41* (6), 1520-1539.
(14) U.S. Environmental Protection Agency. *ECOTOX Knowledgebase*. 2023. https://cfpub.epa.gov/ecotox/ (accessed 2023).

References used to generate Figure 2

(1) Abbas, G.; Murtaza, B.; Bibi, I.; Shahid, M.; Niazi, N. K.; Khan, M. I.; Amjad, M.; Hussain, M.; Natasha. Arsenic Uptake, Toxicity, Detoxification, and Speciation in Plants: Physiological, Biochemical, and Molecular Aspects. *Int J Environ Res Public Health* **2018**, *15* (1). DOI: 10.3390/ijerph15010059.
(2) Abdel-Latif, H. M. R.; Dawood, M. A. O.; Menanteau-Ledouble, S.; El-Matbouli, M. Environmental transformation of n-TiO2 in the aquatic systems and their ecotoxicity in bivalve mollusks: A systematic review. *Ecotoxicology and Environmental Safety* **2020**, *200*, 110776. DOI: 10.1016/j.ecoenv.2020.110776.
(3) Abdelnour, S. A.; Abd El-Hack, M. E.; Khafaga, A. F.; Noreldin, A. E.; Arif, M.; Chaudhry, M. T.; Losacco, C.; Abdeen, A.; Abdel-Daim, M. M. Impacts of rare earth elements on animal health and production: Highlights of cerium and lanthanum. *Sci Total Environ* **2019**, *672*, 1021-1032. DOI: 10.1016/j.scitotenv.2019.02.270.
(4) Abedi, T.; Mojiri, A. Arsenic Uptake and Accumulation Mechanisms in Rice Species. *Plants (Basel)* **2020**, *9* (2). DOI: 10.3390/plants9020129.
(5) Abney, C. W.; Mayes, R. T.; Saito, T.; Dai, S. Materials for the Recovery of Uranium from Seawater. *Chem Rev* **2017**, *117* (23), 13935-14013. DOI: 10.1021/acs.chemrev.7b00355.

(6) Abouchami, W. Secular changes of lead and neodymium in central Pacific seawater recorded by a Fe-Mn crust. *Geochimica et Cosmochimica Acta* **1997**, *61, 3957-3974.* DOI: 10.1016/S0016-7037(97)00218-4.
(7) Adair, J. H.; Krarup, H. G.; Venigalla, S.; Tsukada, T. A review of the aqueous chemistry of the zirconium - Water system to 200 degrees C. *Aqueous Chemistry and Geochemistry of Oxides, Oxyhydroxides, and Related Materials* **1997**, *432*, 101-112. DOI: Doi 10.1557/Proc-432-101.
(8) Adams, J.; Osmond, J.; Rogers, J. The geochemistry of thorium and uranium. *Physics and Chemistry of the Earth* **1959**, *3,* 298-348. DOI: 10.1016/0079-1946(59)90008-4
(9) Agathokleous, E.; Kitao, M.; Calabrese, E. J. Hormetic dose responses induced by lanthanum in plants. *Environ Pollut* **2019**, *244*, 332-341. DOI: 10.1016/j.envpol.2018.10.007.
(10) Ahoulé, D.; Lalanne, F.; Mendret, J.; Brosillon, S.; Maiga, A. Arsenic in African waters: a review. *Water, Air, & Soil Pollution* **2015**, *226,* 302. DOI: 10.1007/s11270-015-2558-4
(11) Aihemaiti, A.; Gao, Y.; Meng, Y.; Chen, X.; Liu, J.; Xiang, H.; Xu, Y.; Jiang, J. Review of plant-vanadium physiological interactions, bioaccumulation, and bioremediation of vanadium-contaminated sites. *Sci Total Environ* **2020**, *712*, 135637. DOI: 10.1016/j.scitotenv.2019.135637.
(12) Alarcon-Poblete, E.; Inostroza-Blancheteau, C.; Alberdi, M.; Rengel, Z.; Reyes-Diaz, M. Molecular regulation of aluminum resistance and sulfur nutrition during root growth. *Planta* **2018**, *247* (1), 27-39. DOI: 10.1007/s00425-017-2805-6.
(13) Alasfar, R. H.; Isaifan, R. J. Aluminum environmental pollution: the silent killer. *Environ Sci Pollut Res Int* **2021**, *28* (33), 44587-44597. DOI: 10.1007/s11356-021-14700-0.
(14) Alejandro, S.; Holler, S.; Meier, B.; Peiter, E. Manganese in Plants: From Acquisition to Subcellular Allocation. *Front Plant Sci* **2020**, *11*, 300. DOI: 10.3389/fpls.2020.00300.
(15) Alessia, A.; Alessandro, B.; Maria, V. G.; Carlos, V. A.; Francesca, B. Challenges for sustainable lithium supply: A critical review. *J Clean Prod* **2021**, *300,* 126954. DOI: 10.1016/j.jclepro.2021.126954.
(16) Alfantazi, A. M.; Moskalyk, R. R. Processing of indium: a review. *Minerals Engineering* **2003**, *16* (8), 687-694. DOI: 10.1016/S0892-6875(03)00168-7.
(17) Ali, W. Insights into the mechanisms of arsenic-selenium interactions and the associated toxicity in plants, animals, and humans: A critical review. *Critical Reviews in Environmental Science and Technology* **2021**, *51,* 704-750. DOI: 10.1080/10643389.2020.1740042
(18) Ali, B. H.; Al Moundhri, M. S. Agents ameliorating or augmenting the nephrotoxicity of cisplatin and other platinum compounds: A review of some recent research. *Food and Chemical Toxicology* **2006**, *44* (8), 1173-1183. DOI: 10.1016/j.fct.2006.01.013.
(19) Ali, S.; Shahzadi, S.; Imtiaz-ud-Din. Anticarcinogenicity and Toxicity of Organotin(IV) Complexes: A Review. *Iranian Journal of Science and Technology Transaction a-Science* **2018**, *42* (A2), 505-524. DOI: 10.1007/s40995-016-0048-1.
(20) Alka, S.; Shahir, S.; Ibrahim, N.; Ndejiko, M. J.; Vo, D. V. N.; Abd Manan, F. Arsenic removal technologies and future trends: A mini review. *J Clean Prod* **2021**, *278,* 123805. DOI: 10.1016/j.jclepro.2020.123805.
(21) Alkurdi, S.; Herath, I.; Bundschuh, J.; Al-Juboori, R.; Vithanage, M.; Mohan, D. Biochar versus bone char for a sustainable inorganic arsenic mitigation in water: What needs to be done in future research? *Environment international* **2019**, *127,* 52-69. DOI: 10.1016/j.envint.2019.03.012
(22) Allahkarami, E.; Rezai, B. Removal of cerium from different aqueous solutions using different adsorbents: A review. *Process Safety and Environmental Protection* **2019**, *124*, 345-362. DOI: 10.1016/j.psep.2019.03.002.

(23) Allahkarami, E.; Rezai, B. A literature review of cerium recovery from different aqueous solutions. *Journal of Environmental Chemical Engineering* **2021**, *9* (1), 104956. DOI: 10.1016/j.jece.2020.104956.
(24) Alshammari, M. S. Solvent extraction of cerium from various solutions by organophosphorus-based extractants: a review. *Desalination and Water Treatment* **2021**, *218*, 345-351. DOI: 10.5004/dwt.2021.26974.
(25) Alvarez, C. C.; Bravo Gomez, M. E.; Hernandez Zavala, A. Hexavalent chromium: Regulation and health effects. *J Trace Elem Med Biol* **2021**, *65*, 126729. DOI: 10.1016/j.jtemb.2021.126729.
(26) Álvarez-Ayuso, E. Stabilization and encapsulation of arsenic-/antimony-bearing mine waste: Overview and outlook of existing techniques. *Critical Reviews in Environmental Science and Technology* **2021**. 3720-3752. DOI: 10.1080/10643389.2021.1944588
(27) Alvial-Hein, G.; Mahandra, H.; Ghahreman, A. Separation and recovery of cobalt and nickel from end of life products via solvent extraction technique: A review. *J Clean Prod* **2021**, *297,* 126592. DOI: 10.1016/j.jclepro.2021.126592.
(28) Aly, M. M.; Hamza, M. F. A Review: Studies on Uranium Removal Using Different Techniques. Overview. *Journal of Dispersion Science and Technology* **2013**, *34* (2), 182-213. DOI: 10.1080/01932691.2012.657954.
(29) Amari, T.; Ghnaya, T.; Abdelly, C. Nickel, cadmium and lead phytotoxicity and potential of halophytic plants in heavy metal extraction. *South African Journal of Botany* **2017**, *111*, 99-110. DOI: 10.1016/j.sajb.2017.03.011.
(30) Ambaye, T. G.; Vaccari, M.; Castro, F. D.; Prasad, S.; Rtimi, S. Emerging technologies for the recovery of rare earth elements (REEs) from the end-of-life electronic wastes: a review on progress, challenges, and perspectives. *Environ Sci Pollut Res Int* **2020**, *27* (29), 36052-36074. DOI: 10.1007/s11356-020-09630-2.
(31) Ameen, N.; Amjad, M.; Murtaza, B.; Abbas, G.; Shahid, M.; Imran, M.; Naeem, M. A.; Niazi, N. K. Biogeochemical behavior of nickel under different abiotic stresses: toxicity and detoxification mechanisms in plants. *Environ Sci Pollut Res Int* **2019**, *26* (11), 10496-10514. DOI: 10.1007/s11356-019-04540-4.
(32) Amilton Barbosa Botelho, J.; Dreisinger, D. B.; Espinosa., D. C. "A review of nickel, copper, and cobalt recovery by chelating ion exchange resins from mining processes and mining tailings.". *"Mining, Metallurgy & Exploration"* **2019**, *36*.
(33) An, H.; Ling, C.; Xu, M.; Hu, M.; Wang, H.; Liu, J.; Song, G.; Liu, J. Oxidative Damage Induced by Nano-titanium Dioxide in Rats and Mice: a Systematic Review and Meta-analysis. *Biol Trace Elem Res* **2020**, *194* (1), 184-202. DOI: 10.1007/s12011-019-01761-z.
(34) Anastopoulos, I.; Anagnostopoulos, V. A.; Bhatnagar, A.; Mitropoulos, A. C.; Kyzas, G. Z. A review for chromium removal by carbon nanotubes. *Chemistry and Ecology* **2017**, *33* (6), 572-588. DOI: 10.1080/02757540.2017.1328503.
(35) Anderson, C. D.; Taylor, P. R.; Anderson, C. G. Extractive metallurgy of rhenium: a review. *Minerals & Metallurgical Processing* **2013**, *30* (1), 59-73. DOI: Doi 10.1007/Bf03402342.
(36) Anderson, S. T. Economics, Helium, and the US Federal Helium Reserve: Summary and Outlook. *Natural Resources Research* **2018**, *27* (4), 455-477. DOI: 10.1007/s11053-017-9359-y.
(37) Andersson, M. Toxicity and tolerance of aluminum in vascular plants. *Water, Air, and Soil Pollution* **1988**, *39,* 439–462. DOI: 10.1007/BF00279487
(38) Anene, F. A.; Jaafar, C. N. A.; Zainol, I.; Hanim, M. A. A.; Suraya, M. T. Biomedical materials: A review of titanium based alloys. *Proceedings of the Institution of Mechanical*

Engineers Part C-Journal of Mechanical Engineering Science **2021**, *235* (19), 3792-3805, 0954406220967694. DOI: 10.1177/0954406220967694.
(39) Angino, E. E.; Long, D. T. *Geochemistry of bismuth*; Dowden, Hutchinson & Ross,Distributed world wide by Academic Press, 1979.
(40) Annu, P.; Sharma, S.; Jain, R.; Raja, A. Review-Pencil Graphite Electrode: An Emerging Sensing Material. *J Electrochem Soc* **2019**, *167*(1). DOI: 10.1149/2.0012003jes.
(41) Ansoborlo, E.; Lebaron-Jacobs, L.; Prat, O. Uranium in drinking-water: A unique case of guideline value increases and discrepancies between chemical and radiochemical guidelines. *Environment International* **2015**, *77*, 1-4. DOI: 10.1016/j.envint.2014.12.011.
(42) Aral, H.; Vecchio-Sadus, A. Toxicity of lithium to humans and the environment--a literature review. *Ecotoxicol Environ Saf* **2008**, *70* (3), 349-356. DOI: 10.1016/j.ecoenv.2008.02.026.
(43) Arregui-Mena, J. D.; Worth, R. N.; Hall, G.; Edmondson, P. D.; Giorla, A. B.; Burchell, T. D. A Review of Finite Element Method Models for Nuclear Graphite Applications. *Archives of Computational Methods in Engineering* **2020**, *27* (1), 331-350. DOI: 10.1007/s11831-018-09310-y.
(44) Arunakumara, K. K. I. U.; Walpola, B. C.; Yoon, M. H. Aluminum Toxicity and Tolerance Mechanism in Cereals and Legumes - A Review. *Journal of the Korean Society for Applied Biological Chemistry* **2013**, *56* (1), 1-9. DOI: 10.1007/s13765-012-2314-z.
(45) Arzuaga, X.; Gehlhaus, M.; Strong, J. Modes of action associated with uranium induced adverse effects in bone function and development. *Toxicol Lett* **2015**, *236* (2), 123-130. DOI: 10.1016/j.toxlet.2015.05.006.
(46) Ashley, K.; Howe, A. M.; Demange, M.; Nygren, O. Sampling and analysis considerations for the determination of hexavalent chromium in workplace air. *J Environ Monit* **2003**, *5* (5), 707-716. DOI: 10.1039/b306105c.
(47) Ashraf, M. A.; Akib, S.; Maah, M. J.; Yusoff, I.; Balkhair, K. S. Cesium-137: Radio-Chemistry, Fate, and Transport, Remediation, and Future Concerns. *Critical Reviews in Environmental Science and Technology* **2014**, *44* (15), 1740-1793. DOI: 10.1080/10643389.2013.790753.
(48) Asic, A.; Kurtovic-Kozaric, A.; Besic, L.; Mehinovic, L.; Hasic, A.; Kozaric, M.; Hukic, M.; Marjanovic, D. Chemical toxicity and radioactivity of depleted uranium: The evidence from in vivo and in vitro studies. *Environ Res* **2017**, *156*, 665-673. DOI: 10.1016/j.envres.2017.04.032.
(49) Aslam, S.; Yousafzai, A. Chromium toxicity in fish: A review article. *Journal of Entomology and Zoology Studies* **2017**, *5, 1483-1488.*
(50) Asmussen, R. M.; Neeway, J. J. The sporadic history of rubidium and its role in corrosion of steel related to nuclear material storage. *Journal of Nuclear Materials* **2020**, *530,* 151914. DOI: 10.1016/j.jnucmat.2019.151914.
(51) Assem, F. L.; Levy, L. S. A review of current toxicological concerns on vanadium pentoxide and other vanadium compounds: gaps in knowledge and directions for future research. *J Toxicol Environ Health B Crit Rev* **2009**, *12* (4), 289-306. DOI: 10.1080/10937400903094166.
(52) Attia, N.; Rostom, D.; Mashal, M. The use of cerium oxide nanoparticles in liver disorders: A double sided coin. *Basic & Clinical Pharmacology & Toxicology* **2021**, 349-363. DOI: 10.1111/bcpt.13700
(53) Au, C.; Benedetto, A.; Aschner, M. Manganese transport in eukaryotes: the role of DMT1. *Neurotoxicology* **2008**, *29* (4), 569-576. DOI: 10.1016/j.neuro.2008.04.022.

(54) Auger, C.; Han, S.; Appanna, V. P.; Thomas, S. C.; Ulibarri, G.; Appanna, V. D. Metabolic reengineering invoked by microbial systems to decontaminate aluminum: implications for bioremediation technologies. *Biotechnol Adv* **2013**, *31* (2), 266-273. DOI: 10.1016/j.biotechadv.2012.11.008.
(55) Avery, S. Microbial interactions with caesium—implications for biotechnology. *Journal of Chemical Technology & Biotechnology International Research in Process, Environmental AND Clean Technology* **1995**, *62,* 3-16. DOI: 10.1002/jctb.280620102
(56) Avery, S. V. Fate of caesium in the environment: Distribution between the abiotic and biotic components of aquatic and terrestrial ecosystems. *Journal of Environmental Radioactivity* **1996**, *30* (2), 139-171. DOI: Doi 10.1016/0265-931x(96)89276-9.
(57) Avudainayagam, S.; Megharaj, M.; Owens, G.; Kookana, R. S.; Chittleborough, D.; Naidu, R. Chemistry of chromium in soils with emphasis on tannery waste sites. *Rev Environ Contam Toxicol* **2003**, *178*, 53-91. DOI: 10.1007/0-387-21728-2_3.
(58) Baalen, V.; R., M. Titanium mobility in metamorphic systems: a review. *Chemical Geology* **1993**, *110*.
(59) Babu, S.; D.; Nidheesh., P. V. A review on electrochemical treatment of arsenic from aqueous medium. *Chemical Engineering Communications* **2021**, *208*.
(60) Bagherifam, S.; Brown, T.; Fellows, C.; Naidu, R. Derivation methods of soils, water and sediments toxicity guidelines: A brief review with a focus on antimony. *Journal of Geochemical Exploration* **2019**, *205*. DOI: 10.1016/j.gexplo.2019.106348
(61) Bagherifam, S.; Brown, T. C.; Fellows, C. M.; Naidu, R. Bioavailability of Arsenic and Antimony in Terrestrial Ecosystems: A Review. *Pedosphere* **2019**, *29* (6), 681-720. DOI: 10.1016/S1002-0160(19)60843-X.
(62) Bailey, J. Fluorine in granitic rocks and melts: a review. *Chemical Geology* **1977**, *19,* 1-42. DOI: 10.1016/0009-2541(77)90002-X
(63) Baker, R. J. Uranium minerals and their relevance to long term storage of nuclear fuels. *Coordination Chemistry Reviews* **2014**, *266*, 123-136. DOI: 10.1016/j.ccr.2013.10.004.
(64) Bakhat, H. F.; Zia, Z.; Fahad, S.; Abbas, S.; Hammad, H. M.; Shahzad, A. N.; Abbas, F.; Alharby, H.; Shahid, M. Arsenic uptake, accumulation and toxicity in rice plants: Possible remedies for its detoxification: A review. *Environ Sci Pollut Res Int* **2017**, *24* (10), 9142-9158. DOI: 10.1007/s11356-017-8462-2.
(65) Bal, B.; Ghosh, S.; Das, A. P. Microbial recovery and recycling of manganese waste and their future application: a review. *Geomicrobiology Journal* **2019**, *36* (1), 85-96. DOI: 10.1080/01490451.2018.1497731.
(66) Balaram, V. Rare earth elements: A review of applications, occurrence, exploration, analysis, recycling, and environmental impact. *Geoscience Frontiers* **2019**, *10* (4), 1285-1303. DOI: 10.1016/j.gsf.2018.12.005.
(67) Balcerzak, M. Methods for the Determination of Platinum Group Elements in Environmental and Biological Materials: A Review. *Critical Reviews in Analytical Chemistry* **2011**, *41* (3), 214-235. DOI: 10.1080/10408347.2011.588922.
(68) Bali, A. S.; Sidhu, G. P. S. Arsenic acquisition, toxicity and tolerance in plants-From physiology to remediation: A review. *Chemosphere* **2021**, *283,* 131050. DOI: 10.1016/j.chemosphere.2021.131050.
(69) Banerjee, P.; Bhattacharya, P. Investigating Cobalt in Soil-plant-animal-human system: Dynamics, Impact and Management. *Journal of Soil Science and Plant Nutrition* **2021**, *21* (3), 2339-2354. DOI: 10.1007/s42729-021-00525-w.

(70) Banerjee, S.; Pillai, M. R.; Knapp, F. F. Lutetium-177 therapeutic radiopharmaceuticals: linking chemistry, radiochemistry, and practical applications. *Chem Rev* **2015**, *115* (8), 2934-2974. DOI: 10.1021/cr500171e.
(71) Banik, A.; Roychowdhury, S.; Biswas, K. The journey of tin chalcogenides towards high-performance thermoelectrics and topological materials. *Chem Commun (Camb)* **2018**, *54* (50), 6573-6590. DOI: 10.1039/c8cc02230e.
(72) Barabanov, V. Geochemistry of tungsten. *International Geology Review* **1971**, *13,* 332-344. DOI: 10.1080/00206817109475439
(73) Baranowska-Wojcik, E.; Szwajgier, D.; Oleszczuk, P.; Winiarska-Mieczan, A. Effects of Titanium Dioxide Nanoparticles Exposure on Human Health-a Review. *Biol Trace Elem Res* **2020**, *193* (1), 118-129. DOI: 10.1007/s12011-019-01706-6.
(74) Barceloux, D. G. Cobalt. *J Toxicol Clin Toxicol* **1999**, *37* (2), 201-206. DOI: 10.1081/clt-100102420.
(75) Barceloux, D. G. Vanadium. *J Toxicol Clin Toxicol* **1999**, *37* (2), 265-278. DOI: 10.1081/clt-100102425.
(76) Barefoot, R. R. Determination of platinum at trace levels in environmental and biological materials. *Environmental Science & Technology* **1997**, *31* (2), 309-314. DOI: DOI 10.1021/es960712i.
(77) Bargar, J. R.; Bernier-Latmani, R.; Giammar, D. E.; Tebo, B. M. Biogenic Uraninite Nanoparticles and Their Importance for Uranium Remediation. *Elements* **2008**, *4* (6), 407-412. DOI: 10.2113/gselements.4.6.407.
(78) Barra, C. M.; Santelli, R. E.; Abrao, J. J.; de la Guardia, M. Arsenic speciation - A review. *Quimica Nova* **2000**, *23* (1), 58-70. DOI: Doi 10.1590/S0100-40422000000100012.
(79) Barral-Fraga, L.; Barral, M. T.; MacNeill, K. L.; Martina-Prieto, D.; Morin, S.; Rodriguez-Castro, M. C.; Tuulaikhuu, B. A.; Guasch, H. Biotic and Abiotic Factors Influencing Arsenic Biogeochemistry and Toxicity in Fluvial Ecosystems: A Review. *Int J Environ Res Public Health* **2020**, *17* (7). DOI: 10.3390/ijerph17072331.
(80) Barras, F.; Fontecave, M. Cobalt stress in Escherichia coli and Salmonella enterica: molecular bases for toxicity and resistance. *Metallomics* **2011**, *3* (11), 1130-1134. DOI: 10.1039/c1mt00099c.
(81) Barry, T. S.; Uysal, T.; Birinci, M.; Erdemoglu, M. Thermal and Mechanical Activation in Acid Leaching Processes of Non-bauxite Ores Available for Alumina Production-A Review. *Mining Metallurgy & Exploration* **2019**, *36* (3), 557-569. DOI: 10.1007/s42461-018-0025-7.
(82) Barsukova, M. O.; Sapchenko, S. A.; Dybtsev, D. N.; Fedin, V. P. Scandium-organic frameworks: progress and prospects. *Russian Chemical Reviews* **2018**, *87* (11), 1139-1167. DOI: 10.1070/Rcr4826.
(83) Barton, M. D.; Young, S. Non-pegmatitic deposits of beryllium: Mineralogy, geology, phase equilibria and origin. *Beryllium: Mineralogy, Petrology, and Geochemistry* **2002**, *50*, 591-691. DOI: DOI 10.2138/rmg.2002.50.14.
(84) Basak, B. B.; Sarkar, B.; Biswas, D. R.; Sarkar, S.; Sanderson, P.; Naidu, R. Bio-Intervention of Naturally Occurring Silicate Minerals for Alternative Source of Potassium: Challenges and Opportunities. *Advances in Agronomy, Vol 141* **2017**, *141*, 115-145. DOI: 10.1016/bs.agron.2016.10.016.
(85) Bastías, J.; Beldarrain, T. Arsenic translocation in rice cultivation and its implication for human health. *Chilean journal of agricultural research* **2016**, *76*. DOI: 10.4067/S0718-58392016000100016

(86) Basu, A.; Saha, D.; Saha, R.; Ghosh, T.; Saha, B. A review on sources, toxicity and remediation technologies for removing arsenic from drinking water. *Research on Chemical Intermediates* **2014**, *40* (2), 447-485. DOI: 10.1007/s11164-012-1000-4.
(87) Bataille, C. P.; Crowley, B. E.; Wooller, M. J.; Bowen, G. J. Advances in global bioavailable strontium isoscapes. *Palaeogeography Palaeoclimatology Palaeoecology* **2020**, *555,* 109849. DOI: 10.1016/j.palaeo.2020.109849.
(88) Bayuo, J. An extensive review on chromium (vi) removal using natural and agricultural wastes materials as alternative biosorbents. *Journal of Environmental Health Science and Engineering* **2021**, *19* (1), 1193-1207. DOI: 10.1007/s40201-021-00641-w.
(89) Behera, S. K.; Mulaba-Bafubiandi, A. F. Advances in microbial leaching processes for nickel extraction from lateritic minerals - A review. *Korean Journal of Chemical Engineering* **2015**, *32* (8), 1447-1454. DOI: 10.1007/s11814-015-0085-z.
(90) Belzile, N.; Chen, Y. W. Tellurium in the environment: A critical review focused on natural waters, soils, sediments and airborne particles. *Applied Geochemistry* **2015**, *63*, 83-92. DOI: 10.1016/j.apgeochem.2015.07.002.
(91) Belzile, N.; Chen, Y. W.; Filella, M. Human Exposure to Antimony: I. Sources and Intake. *Critical Reviews in Environmental Science and Technology* **2011**, *41* (14), 1309-1373. DOI: 10.1080/10643381003608227.
(92) Bem, H.; Bou-Rabee, F. Environmental and health consequences of depleted uranium use in the 1991 Gulf War. *Environ Int* **2004**, *30* (1), 123-134. DOI: 10.1016/S0160-4120(03)00151-X.
(93) Ben Fekih, I.; Zhang, C.; Li, Y. P.; Zhao, Y.; Alwathnani, H. A.; Saquib, Q.; Rensing, C.; Cervantes, C. Distribution of Arsenic Resistance Genes in Prokaryotes. *Front Microbiol* **2018**, *9*, 2473. DOI: 10.3389/fmicb.2018.02473.
(94) Bennet, J.; Breen, C. The aluminium signal: new dimensions to mechanisms of aluminium tolerance. *Plant and soil* **1991**, *134,* 153–166. DOI: 10.1007/BF00010728
(95) Bentley, R.; Chasteen, T. G. Microbial methylation of metalloids: arsenic, antimony, and bismuth. *Microbiol Mol Biol Rev* **2002**, *66* (2), 250-271. DOI: 10.1128/MMBR.66.2.250-271.2002.
(96) Bentley, R. A. Strontium isotopes from the earth to the archaeological skeleton: A review. *Journal of Archaeological Method and Theory* **2006**, *13* (3), 135-187. DOI: 10.1007/s10816-006-9009-x.
(97) Beraldo, H. Pharmacological applications of non-radioactive indium(III) complexes: A field yet to be explored. *Coordination Chemistry Reviews* **2020**, *419,* 213375. DOI: 10.1016/j.ccr.2020.213375.
(98) Bešić, L.; Muhović, I.; Mrkulić, F.; Spahić, L.; Omanović, A.; Kurtovic-Kozaric, A. Meta-analysis of depleted uranium levels in the Middle East region. *Journal of environmental radioactivity* **2018**, *192*.
(99) Bevers, L. E.; Hagedoorn, P. L.; Hagen, W. R. The bioinorganic chemistry of tungsten. *Coordination Chemistry Reviews* **2009**, *253* (3-4), 269-290. DOI: 10.1016/j.ccr.2008.01.017.
(100) Bhattacharjee, P.; Banerjee, M.; Giri, A. K. Role of genomic instability in arsenic-induced carcinogenicity. A review. *Environ Int* **2013**, *53*, 29-40. DOI: 10.1016/j.envint.2012.12.004.
(101) Bhattacharya, P.; Welch, A. H.; Stollenwerk, K. G.; McLaughlin, M. J.; Bundschuh, J.; Panaullah, G. Arsenic in the environment: Biology and Chemistry. *Sci Total Environ* **2007**, *379* (2-3), 109-120. DOI: 10.1016/j.scitotenv.2007.02.037.
(102) Bielicka, A.; Bojanowska, I.; Wisniewski, A. Two faces of chromium - Pollutant and bioelement. *Polish Journal of Environmental Studies* **2005**, *14* (1), 5-10.

(103) Bigham, J. M.; Nordstrom, D. K. Iron and aluminum hydroxysulfates from acid sulfate waters. *Sulfate Minerals - Crystallography, Geochemistry and Environmental Significance* **2000**, *40*, 351-403. DOI: DOI 10.2138/rmg.2000.40.7.
(104) Bijvelds, M. J.; Velden, J. A.; Kolar, Z. I.; Flik, G. Magnesium transport in freshwater teleosts. *J Exp Biol* **1998**, *201 (Pt 13)*, 1981-1990. DOI: 10.1242/jeb.201.13.1981.
(105) Binet, M. T.; Adams, M. S.; Gissi, F.; Golding, L. A.; Schlekat, C. E.; Garman, E. R.; Merrington, G.; Stauber, J. L. Toxicity of nickel to tropical freshwater and sediment biota: A critical literature review and gap analysis. *Environ Toxicol Chem* **2018**, *37* (2), 293-317. DOI: 10.1002/etc.3988.
(106) Bischoff, N. S.; de Kok, T. M.; Sijm, D. T. H. M.; van Breda, S. G.; Briede, J. J.; Castenmiller, J. J. M.; Opperhuizen, A.; Chirino, Y. I.; Dirven, H.; Gott, D.; Houdeau, E.; Oomen, A.G.; Poulsen, M.; Rogler, G.; van Loveren, H. Possible Adverse Effects of Food Additive E171 (Titanium Dioxide) Related to Particle Specific Human Toxicity, Including the Immune System. *International Journal of Molecular Sciences* **2021**, *22* (1), 207. DOI: 10.3390/ijms22010207.
(107) Bissen, M; Frimmel, H. Arsenic—a review. Part I: occurrence, toxicity, speciation, mobility. *Acta hydrochimica et hydrobiologica* **2003**, *31,* 9-18. DOI: 10.1002/aheh.200390025
(108) Bjorklund, G.; Chartrand, M. S.; Aaseth, J. Manganese exposure and neurotoxic effects in children. *Environ Res* **2017**, *155*, 380-384. DOI: 10.1016/j.envres.2017.03.003.
(109) Bjorklund, G.; Pivina, L.; Dadar, M.; Semenova, Y.; Rahman, M. M.; Chirumbolo, S.; Aaseth, J. Depleted uranium and Gulf War Illness: Updates and comments on possible mechanisms behind the syndrome. *Environ Res* **2020**, *181*, 108927. DOI: 10.1016/j.envres.2019.108927.
(110) Bjorklund, G.; Semenova, Y.; Pivina, L.; Dadar, M.; Rahman, M. M.; Aaseth, J.; Chirumbolo, S. Uranium in drinking water: a public health threat. *Arch Toxicol* **2020**, *94* (5), 1551-1560. DOI: 10.1007/s00204-020-02676-8.
(111) Blichert-Toft, J. On the Lu-Hf isotope geochemistry of silicate rocks. *Geostandards Newsletter* **2001**, *25*.
(112) Blinova, I.; Muna, M.; Heinlaan, M.; Lukjanova, A.; Kahru, A. Potential Hazard of Lanthanides and Lanthanide-Based Nanoparticles to Aquatic Ecosystems: Data Gaps, Challenges and Future Research Needs Derived from Bibliometric Analysis. *Nanomaterials (Basel)* **2020**, *10* (2). DOI: 10.3390/nano10020328.
(113) Bloch, K.; Pardesi, K.; Satriano, C.; Ghosh, S. Bacteriogenic Platinum Nanoparticles for Application in Nanomedicine. *Front Chem* **2021**, *9*, 624344. DOI: 10.3389/fchem.2021.624344.
(114) Blunden, S.; Wallace, T. Tin in canned food: a review and understanding of occurrence and effect. *Food Chem Toxicol* **2003**, *41* (12), 1651-1662. DOI: 10.1016/s0278-6915(03)00217-5.
(115) Li, B.; Ma, L.; Luo, N.; Li, S.; Chen, Y.; Zhang, J. Extraction and Separation of Uranium via Solid Phase Extraction. *Progress in Chemistry* **2020**, *32,* 1316-1333. DOI: 10.7536/PC200219
(116) Bodar, C. W.; Pronk, M. E.; Sijm, D. T. The European Union risk assessment on zinc and zinc compounds: the process and the facts. *Integr Environ Assess Manag* **2005**, *1* (4), 301-319..
(117) Bojorquez-Quintal, E.; Escalante-Magana, C.; Echevarria-Machado, I.; Martinez-Estevez, M. Aluminum, a Friend or Foe of Higher Plants in Acid Soils. *Front Plant Sci* **2017**, *8*, 1767. DOI: 10.3389/fpls.2017.01767.

(118) Boland, S.; Hussain, S.; Baeza-Squiban, A. Carbon black and titanium dioxide nanoparticles induce distinct molecular mechanisms of toxicity. *Wiley Interdiscip Rev Nanomed Nanobiotechnol* **2014**, *6* (6), 641-652. DOI: 10.1002/wnan.1302.
(119) Bomhard, E. M. The toxicology of indium tin oxide. *Environ Toxicol Pharmacol* **2016**, *45*, 282-294. DOI: 10.1016/j.etap.2016.06.011.
(120) Bomhard, E. M. The toxicology of indium oxide. *Environ Toxicol Pharmacol* **2018**, *58*, 250-258. DOI: 10.1016/j.etap.2018.02.003.
(121) Bomhard, E. M. The toxicology of gallium oxide in comparison with gallium arsenide and indium oxide. *Environ Toxicol Pharmacol* **2020**, *80*, 103437. DOI: 10.1016/j.etap.2020.103437.
(122) Bomhard, E. M.; Gelbke, H. P.; Schenk, H.; Williams, G. M.; Cohen, S. M. Evaluation of the carcinogenicity of gallium arsenide. *Crit Rev Toxicol* **2013**, *43* (5), 436-466. DOI: 10.3109/10408444.2013.792329.
(123) Bondu, R.; Cloutier, V.; Rosa, E.; Benzaazoua, M. A Review and Evaluation of the Impacts of Climate Change on Geogenic Arsenic in Groundwater from Fractured Bedrock Aquifers. *Water Air and Soil Pollution* **2016**, *227* (9), 296. DOI: 10.1007/s11270-016-2936-6.
(124) Boni, M.; Mondillo, N. The "Calamines" and the "Others": The great family of supergene nonsulfide zinc ores. *Ore Geology Reviews* **2015**, *67*, 208-233. DOI: 10.1016/j.oregeorev.2014.10.025.
(125) Boorboori, M. R.; Gao, Y. J.; Wang, H.; Fang, C. X. Usage of Si, P, Se, and Ca Decrease Arsenic Concentration/Toxicity in Rice, a Review. *Applied Sciences-Basel* **2021**, *11* (17), 8090. DOI: 10.3390/app11178090.
(126) Borchers, N.; Clark, S.; Horstmann, B.; Jayasayee, K.; Juel, M.; Stevens, P. Innovative zinc-based batteries. *Journal of Power Sources* **2021**, *484,* 229309. DOI: 10.1016/j.jpowsour.2020.229309.
(127) Bordbar; H.; Yousefi, A.; Abedini, H. Production of titanium tetrachloride (TiCl4) from titanium ores: A review. *Polyolefins Journal* **2017**, *4*. DOI: 10.22063/POJ.2017.1453
(128) Boreham, C.; Edwards, D.; Poreda, R.; Darrah, T.; Zhu, R.; Grosjean, E.; Main, P.; Waltenberg, K.; Henson, P. Helium in the Australian liquefied natural gas economy. *The APPEA Journal* **2018**, *58,* 209-237. DOI: 10.1071/AJ17049
(129) Bose, J.; Babourina, O.; Rengel, Z. Role of magnesium in alleviation of aluminium toxicity in plants. *J Exp Bot* **2011**, *62* (7), 2251-2264. DOI: 10.1093/jxb/erq456.
(130) Bounouri, Y.; Berkani, M.; Zamouche, A.; Rycerz, L. Optimization and modeling of synthesis parameters of neodymium(III) bromide by dry method using full factorial design analysis. *Arabian Journal of Chemistry* **2020**, *13* (1), 366-376. DOI: 10.1016/j.arabjc.2017.05.003.
(131) Bourdon, B.; Turner, S.; Henderson, G. M.; Lundstrom, C. C. Introduction to U-series geochemistry. *Uranium-Series Geochemistry* **2003**, *52*, 1-21. DOI: Doi 10.2113/0520001.
(132) Bowell, R. J.; Alpers, C. N.; Jamieson, H. E.; Nordstrom, D. K.; Majzlan, J. The Environmental Geochemistry of Arsenic - An Overview -. *Arsenic: Environmental Geochemistry, Mineralogy, and Microbiology* **2014**, *79*, 1-16. DOI: 10.2138/rmg.2014.79.1.
(133) Bowles, J. F. W.; Suarez, S. The formation of alluvial platinum-group minerals: present knowledge and the way ahead. *Mineralogical Magazine* **2021**, *85* (1), 12-21. DOI: 10.1180/mgm.2021.3.
(134) Brammer, H.; Ravenscroft, P. Arsenic in groundwater: a threat to sustainable agriculture in South and South-east Asia. *Environ Int* **2009**, *35* (3), 647-654. DOI: 10.1016/j.envint.2008.10.004.

(135) Brandukova, N. E.; Vygodskii, Y. S.; Vinogradova, S. V. Applications of the Samarium Diiodide in Organic and Polymer Synthesis. *Uspekhi Khimii* **1994**, *63* (4), 361-372.
(136) Brazdil, J. F.; Teller, R. G.; Grasselli, R. K.; Kostiner, E. Structural and Thermodynamic Basis for Catalytic Behavior of Bismuth-Cerium Molybdate Selective Oxidation Catalysts. *Acs Symposium Series* **1985**, *279*, 57-74.
(137) Breit, G. N.; Wanty, R. B. Vanadium Accumulation in Carbonaceous Rocks - a Review of Geochemical Controls during Deposition and Diagenesis. *Chemical Geology* **1991**, *91* (2), 83-97. DOI: Doi 10.1016/0009-2541(91)90083-4.
(138) Briner, W. The toxicity of depleted uranium. *Int J Environ Res Public Health* **2010**, *7* (1), 303-313. DOI: 10.3390/ijerph7010303.
(139) Brinkel, J.; Khan, M. H.; Kraemer, A. A systematic review of arsenic exposure and its social and mental health effects with special reference to Bangladesh. *Int J Environ Res Public Health* **2009**, *6* (5), 1609-1619. DOI: 10.3390/ijerph6051609.
(140) Brix, K. V.; Schlekat, C. E.; Garman, E. R. The mechanisms of nickel toxicity in aquatic environments: An adverse outcome pathway analysis. *Environ Toxicol Chem* **2017**, *36* (5), 1128-1137. DOI: 10.1002/etc.3706.
(141) Brugge, D.; de Lemos, J. L.; Oldmixon, B. Exposure pathways and health effects associated with chemical and radiological toxicity of natural uranium: a review. *Rev Environ Health* **2005**, *20* (3), 177-193. DOI: 10.1515/reveh.2005.20.3.177.
(142) Brunjes, R.; Hofmann, T. Anthropogenic gadolinium in freshwater and drinking water systems. *Water Res* **2020**, *182*, 115966. DOI: 10.1016/j.watres.2020.115966.
(143) Brunner, I.; Sperisen, C. Aluminum exclusion and aluminum tolerance in woody plants. *Front Plant Sci* **2013**, *4*, 172. DOI: 10.3389/fpls.2013.00172.
(144) Buchner, M. R. Recent Contributions to the Coordination Chemistry of Beryllium. *Chemistry* **2019**, *25* (52), 12018-12036. DOI: 10.1002/chem.201901766.
(145) Buckingham, R.; Asset, T.; Atanassov, P. Aluminum-air batteries: A review of alloys, electrolytes and design. *Journal of Power Sources* **2021**, *498,* 229762. DOI: 10.1016/j.jpowsour.2021.229762.
(146) Buettner, K. M.; Valentine, A. M. Bioinorganic chemistry of titanium. *Chem Rev* **2012**, *112* (3), 1863-1881. DOI: 10.1021/cr1002886.
(147) Bundschuh, J.; Maity, J. P. Geothermal arsenic: Occurrence, mobility and environmental implications. *Renewable & Sustainable Energy Reviews* **2015**, *42*, 1214-1222. DOI: 10.1016/j.rser.2014.10.092.
(148) Bundschuh, J.; Nath, B.; Bhattacharya, P.; Liu, C. W.; Armienta, M. A.; Moreno Lopez, M. V.; Lopez, D. L.; Jean, J. S.; Cornejo, L.; Lauer Macedo, L. F.; Filho, A.T. Arsenic in the human food chain: the Latin American perspective. *Sci Total Environ* **2012**, *429*, 92-106. DOI: 10.1016/j.scitotenv.2011.09.069.
(149) Burdige, D. J. The Biogeochemistry of Manganese and Iron Reduction in Marine-Sediments. *Earth-Science Reviews* **1993**, *35* (3), 249-284. DOI: 10.1016/0012-8252(93)90040-E.
(150) Burger, A.; Lichtscheidl, I. Stable and radioactive cesium: A review about distribution in the environment, uptake and translocation in plants, plant reactions and plants' potential for bioremediation. *Science of the Total Environment* **2018**, *618*, 1459-1485. DOI: 10.1016/j.scitotenv.2017.09.298.
(151) Burger, A.; Lichtscheidl, I. Strontium in the environment: Review about reactions of plants towards stable and radioactive strontium isotopes. *Science of the Total Environment* **2019**, *653*, 1458-1512. DOI: 10.1016/j.scitotenv.2018.10.312.

(152) Burlakovs, J.; Vincevica-Gaile, Z.; Krievans, M.; Jani, Y.; Horttanainen, M.; Pehme, K. M.; Dace, E.; Setyobudi, R. H.; Pilecka, J.; Denafas, G.; Grinfelde, I.; Bhatnagar, A.; Rud, V.; Rudovica, V.; Mersky, R. L.; Anne, O.; Kriipsalu, M.; Ozola-Davidane, R.; Tamm, T.; Klavins, M. Platinum Group Elements in Geosphere and Anthroposphere: Interplay among the Global Reserves, Urban Ores, Markets and Circular Economy. *Minerals* **2020**, *10* (6), 558. DOI: 10.3390/min10060558.
(153) Butcher, D. J. Environmental applications of arsenic speciation using atomic spectrometry detection. *Applied Spectroscopy Reviews* **2007**, *42* (1), 1-22. DOI: 10.1080/05704920600939398.
(154) Černý, P. Mineralogy of beryllium in granitic pegmatites. *Reviews in Mineralogy and Geochemistry* **2002**, *50*.
(155) Cabello, J. Lithium Brine Production, Reserves, Resources and Exploration in Chile: An Updated Review. *Ore Geology Reviews* **2020**, 128.
(156) Cabot, C.; Martos, S.; Llugany, M.; Gallego, B.; Tolra, R.; Poschenrieder, C. A Role for Zinc in Plant Defense Against Pathogens and Herbivores. *Front Plant Sci* **2019**, *10*, 1171. DOI: 10.3389/fpls.2019.01171.
(157) Cabral, A. R.; Lehmann, B. Seleniferous minerals of palladium and platinum from ouro preto-bearing mineralisation in Brazil. *Ore Geology Reviews* **2007**, *32* (3-4), 681-688. DOI: 10.1016/j.oregeorev.2005.02.006.
(158) Calvert, S. E.; Pedersen, T. F. Sedimentary geochemistry of manganese: Implications for the environment of formation of manganiferous black shales. *Economic Geology and the Bulletin of the Society of Economic Geologists* **1996**, *91* (1), 36-47. DOI: 10.2113/gsecongeo.91.1.36.
(159) Campbell, K. M.; Gallegos, T. J.; Landa, E. R. Biogeochemical aspects of uranium mineralization, mining, milling, and remediation. *Applied Geochemistry* **2015**, *57*, 206-235. DOI: 10.1016/j.apgeochem.2014.07.022.
(160) Campbell, K. M.; Nordstrom, D. K. Arsenic Speciation and Sorption in Natural Environments. *Arsenic: Environmental Geochemistry, Mineralogy, and Microbiology* **2014**, *79*, 185-216. DOI: 10.2138/rmg.2014.79.3.
(161) Cannata, J. B.; Domingo, J. L. Aluminum toxicity in mammals: a minireview. *Vet Hum Toxicol* **1989**, *31* (6), 577-583.
(162) Caravan, P. Strategies for increasing the sensitivity of gadolinium based MRI contrast agents. *Chem Soc Rev* **2006**, *35* (6), 512-523. DOI: 10.1039/b510982p.
(163) Caravan, P.; Ellison, J. J.; McMurry, T. J.; Lauffer, R. B. Gadolinium(III) chelates as MRI contrast agents: Structure, dynamics, and applications. *Chemical Reviews* **1999**, *99* (9), 2293-2352. DOI: 10.1021/cr980440x.
(164) Carlo, D.; E. Soil quality and vegetation performance indicators for sustainable rehabilitation of bauxite residue disposal areas: a review. *Soil Research* **2019**, *57*.
(165) Carolan, D. Recent advances in germanium nanocrystals: Synthesis, optical properties and applications. *Progress in Materials Science* **2017**, *90*, 128-158. DOI: 10.1016/j.pmatsci.2017.07.005.
(166) Carr, M. H.; Turekian, K. K. The Geochemistry of Cobalt. *Geochimica Et Cosmochimica Acta* **1961**, *23* (1-2), 9-60. DOI: Doi 10.1016/0016-7037(61)90087-4.
(167) Casey, W. H.; Phillips, B. L.; Furrer, G. Aqueous aluminum polynuclear complexes and nanoclusters: A review. *Nanoparticles and the Environment* **2001**, *44*, 167-190. DOI: 10.2138/rmg.2001.44.05.
(168) Cassee, F. R.; van Balen, E. C.; Singh, C.; Green, D.; Muijser, H.; Weinstein, J.; Dreher, K. Exposure, Health and Ecological Effects Review of Engineered Nanoscale Cerium and

Cerium Oxide Associated with its Use as a Fuel Additive. *Critical Reviews in Toxicology* **2011**, *41* (3), 213-229. DOI: 10.3109/10408444.2010.529105.
(169) Cavalca, L.; Corsini, A.; Zaccheo, P.; Andreoni, V.; Muyzer, G. Microbial transformations of arsenic: perspectives for biological removal of arsenic from water. *Future Microbiol* **2013**, *8* (6), 753-768. DOI: 10.2217/fmb.13.38.
(170) Cempel, M.; Nikel, G. Nickel: A review of its sources and environmental toxicology. *Polish Journal of Environmental Studies* **2006**, *15* (3), 375-382.
(171) Cervantes, C.; Campos-Garcia, J.; Devars, S.; Gutierrez-Corona, F.; Loza-Tavera, H.; Torres-Guzman, J. C.; Moreno-Sanchez, R. Interactions of chromium with microorganisms and plants. *FEMS Microbiol Rev* **2001**, *25* (3), 335-347. DOI: 10.1111/j.1574-6976.2001.tb00581.x.
(172) Cervantes, C.; Ji, G.; Ramirez, J. L.; Silver, S. Resistance to arsenic compounds in microorganisms. *FEMS Microbiol Rev* **1994**, *15* (4), 355-367. DOI: 10.1111/j.1574-6976.1994.tb00145.x.
(173) Chakraborti, D; Rahman, M.; Paul, K.; Chowdhury, U.; Sengupta, M.; Lodh, D.; Chanda, C.; Saha, K.; Mukherjee, S. Arsenic calamity in the Indian subcontinent: what lessons have been learned? *Talanta* **2002**, 58.
(174) Chandra, J.; Keshavkant, S. Mechanisms underlying the phytotoxicity and genotoxicity of aluminum and their alleviation strategies: A review. *Chemosphere* **2021**, *278,* 130384. DOI: 10.1016/j.chemosphere.2021.130384.
(175) Chandra, M.; Yu, D. W.; Tian, Q. H.; Guo, X. Y. Recovery of Cobalt from Secondary Resources: A Comprehensive Review. *Mineral Processing and Extractive Metallurgy Review* **2022**, *43* (6), 679-700. DOI: 10.1080/08827508.2021.1916927.
(176) Chandrakar, V.; Naithani, S. C.; Keshavkant, S. Arsenic-induced metabolic disturbances and their mitigation mechanisms in crop plants: A review. *Biologia* **2016**, *71* (4), 367-377. DOI: 10.1515/biolog-2016-0052.
(177) Chang, X. H.; Zhao, H. J.; Gao, J. X.; Chen, L. J.; Zhu, A.; Wang, C.; Yu, S.; Ren, X. L.; Ge, P. F.; Sun, Y. B. Pulmonary toxicity of exposure to nano nickel oxide. *Micro & Nano Letters* **2018**, *13* (6), 733-738. DOI: 10.1049/mnl.2017.0802.
(178) Chankeshwara, S. V.; Indrigo, E.; Bradley, M. Palladium-mediated chemistry in living cells. *Curr Opin Chem Biol* **2014**, *21*, 128-135. DOI: 10.1016/j.cbpa.2014.07.007.
(179) Charbonnier, Q.; Moynier, F.; Bouchez, J. Barium isotope cosmochemistry and geochemistry. *Sci Bull (Beijing)* **2018**, *63* (6), 385-394. DOI: 10.1016/j.scib.2018.01.018.
(180) Chasapis, C. T.; Ntoupa, P. A.; Spiliopoulou, C. A.; Stefanidou, M. E. Recent aspects of the effects of zinc on human health. *Arch Toxicol* **2020**, *94* (5), 1443-1460. DOI: 10.1007/s00204-020-02702-9.
(181) Chasteen, T. G.; Fuentes, D. E.; Tantalean, J. C.; Vasquez, C. C. Tellurite: history, oxidative stress, and molecular mechanisms of resistance. *FEMS Microbiol Rev* **2009**, *33* (4), 820-832. DOI: 10.1111/j.1574-6976.2009.00177.x.
(182) Chauhan, D. K.; Yadav, V.; Vaculik, M.; Gassmann, W.; Pike, S.; Arif, N.; Singh, V. P.; Deshmukh, R.; Sahi, S.; Tripathi, D. K. Aluminum toxicity and aluminum stress-induced physiological tolerance responses in higher plants. *Crit Rev Biotechnol* **2021**, *41* (5), 715-730. DOI: 10.1080/07388551.2021.1874282.
(183) Chehabeddine, L.; Al Saleh, T.; Baalbaki, M.; Saleh, E.; Khoury, S. J.; Hannoun, S. Cumulative administrations of gadolinium-based contrast agents: risks of accumulation and toxicity of linear vs macrocyclic agents. *Crit Rev Toxicol* **2019**, *49* (3), 262-279. DOI: 10.1080/10408444.2019.1592109.

(184) Chelgani, S. C.; Rudolph, M.; Kratzsch, R.; Sandmann, D.; Gutzmer, J. A Review of Graphite Beneficiation Techniques. *Mineral Processing and Extractive Metallurgy Review* **2016**, *37* (1), 58-68. DOI: 10.1080/08827508.2015.1115992.
(185) Chelucci, G.; Baldino, S.; Baratta, W. Recent advances in osmium-catalyzed hydrogenation and dehydrogenation reactions. *Acc Chem Res* **2015**, *48* (2), 363-379. DOI: 10.1021/ar5003818.
(186) Chen, X; Kumari, D.; Coa, C.; Achal, V. A review on remediation technologies for nickel-contaminated soil. *Human and Ecological Risk Assessment: An International Journal* **2019**, *26(3)*, 571-585.
(187) Chen, A.; Holt-Hindle, P. Platinum-based nanostructured materials: synthesis, properties, and applications. *Chem Rev* **2010**, *110* (6), 3767-3804. DOI: 10.1021/cr9003902.
(188) Chen, C. Y.; Huang, D. J.; Liu, J. Q. Functions and Toxicity of Nickel in Plants: Recent Advances and Future Prospects. *Clean-Soil Air Water* **2009**, *37* (4-5), 304-313. DOI: 10.1002/clen.200800199.
(189) Chen, H.; Chen, H.; Chen, Z. A review of in situ phytoextraction of rare earth elements from contaminated soils. *Int J Phytoremediation* **2022**, *24* (6), 557-566. DOI: 10.1080/15226514.2021.1957770.
(190) Chen, J.; Halls, C.; Stanley, C. J. Tin-Bearing Skarns of South China - Geological Setting and Mineralogy. *Ore Geology Reviews* **1992**, *7* (3), 225-248. DOI: Doi 10.1016/0169-1368(92)90006-7.
(191) Chen, J.; Tian, Y. Hexavalent chromium reducing bacteria: mechanism of reduction and characteristics. *Environ Sci Pollut Res Int* **2021**, *28* (17), 20981-20997. DOI: 10.1007/s11356-021-13325-7.
(192) Chen, L.; Liu, J.; Zhang, W.; Zhou, J.; Luo, D.; Li, Z. Uranium (U) source, speciation, uptake, toxicity and bioremediation strategies in soil-plant system: A review. *J Hazard Mater* **2021**, *413*, 125319. DOI: 10.1016/j.jhazmat.2021.125319.
(193) Chen, L.; Liu, J. R.; Hu, W. F.; Gao, J.; Yang, J. Y. Vanadium in soil-plant system: Source, fate, toxicity, and bioremediation. *J Hazard Mater* **2021**, *405*, 124200. DOI: 10.1016/j.jhazmat.2020.124200.
(194) Chen, L. S.; Qi, Y. P.; Jiang, H. X.; Yang, L. T.; Yang, G. H. Photosynthesis and photoprotective systems of plants in response to aluminum toxicity. *African Journal of Biotechnology* **2010**, *9* (54), 9237-9247.
(195) Chen, P.; Culbreth, M.; Aschner, M. Exposure, epidemiology, and mechanism of the environmental toxicant manganese. *Environ Sci Pollut Res Int* **2016**, *23* (14), 13802-13810. DOI: 10.1007/s11356-016-6687-0.
(196) Chen, Q. Y.; Costa, M. Arsenic: A Global Environmental Challenge. *Annu Rev Pharmacol Toxicol* **2021**, *61*, 47-63. DOI: 10.1146/annurev-pharmtox-030220-013418.
(197) Chen, S. Q.; Hu, J. Y.; Han, S. J.; Guo, Y. F.; Belzile, N.; Deng, T. L. A review on emerging composite materials for cesium adsorption and environmental remediation on the latest decade. *Separation and Purification Technology* **2020**, *251,* 117340. DOI: 10.1016/j.seppur.2020.117340.
(198) Chen, X.; Zhou, Z.; Karahan, H. E.; Shao, Q.; Wei, L.; Chen, Y. Recent Advances in Materials and Design of Electrochemically Rechargeable Zinc-Air Batteries. *Small* **2018**, *14* (44), e1801929. DOI: 10.1002/smll.201801929.

(199) Chen, Z.; Li, J.; Chen, L.; Zhao, F.; Jiang, J. The Pneumotoxic Effect and Indium Ion Release Induced by Indium Tin Oxide Nanoparticles. *J Nanosci Nanotechnol* **2019**, *19* (8), 4357-4365. DOI: 10.1166/jnn.2019.16924.
(200) Chen, Z. J.; Han, S.; Zhou, S. P.; Feng, H. M.; Liu, Y.; Jia, G. Review article Review of health safety aspects of titanium dioxide nanoparticles in food application. *Nanoimpact* **2020**, *18,* 100224. DOI: 10.1016/j.impact.2020.100224.
(201) Cheong, S.; Watt, J. D.; Tilley, R. D. Shape control of platinum and palladium nanoparticles for catalysis. *Nanoscale* **2010**, *2* (10), 2045-2053. DOI: 10.1039/c0nr00276c.
(202) Choi, J. H.; Mao, Y.; Chang, J. P. Development of hafnium based high-k materials-A review. *Materials Science & Engineering R-Reports* **2011**, *72* (6), 97-136. DOI: 10.1016/j.mser.2010.12.001.
(203) Choong, T. S. Y.; Chuah, T. G.; Robiah, Y.; Koay, F. L. G.; Azni, I. Arsenic toxicity, health hazards and removal techniques from water: an overview. *Desalination* **2007**, *217* (1-3), 139-166. DOI: 10.1016/j.desal.2007.01.015.
(204) Choppala, G.; Bolan, N.; Park, J. H. Chromium Contamination and Its Risk Management in Complex Environmental Settings. *Advances in Agronomy, Vol 120* **2013**, *120*, 129-172. DOI: 10.1016/B978-0-12-407686-0.00002-6.
(205) Choubey, P. K.; Chung, K. S.; Kim, M. S.; Lee, J. C.; Srivastava, R. R. Advance review on the exploitation of the prominent energy-storage element Lithium. Part II: From sea water and spent lithium ion batteries (LIBs). *Minerals Engineering* **2017**, *110*, 104-121. DOI: 10.1016/j.mineng.2017.04.008.
(206) Choubey, P. K.; Kim, M. S.; Srivastava, R. R.; Lee, J. C.; Lee, J. Y. Advance review on the exploitation of the prominent energy-storage element: Lithium. Part I: From mineral and brine resources. *Minerals Engineering* **2016**, *89*, 119-137. DOI: 10.1016/j.mineng.2016.01.010.
(207) Choudhury, R.; Srivastava, S. Zinc resistance mechanisms in bacteria. *Current Science* **2001**, *81* (7), 768-775.
(208) Chowdhury, A.; Adak, M. K.; Mukherjee, A.; Dhak, P.; Khatun, J.; Dhak, D. A critical review on geochemical and geological aspects of fluoride belts, fluorosis and natural materials and other sources for alternatives to fluoride exposure. *Journal of Hydrology* **2019**, *574*, 333-359. DOI: 10.1016/j.jhydrol.2019.04.033.
(209) Christy, A. G.; Mills, S. J.; Kampf, A. R. A review of the structural architecture of tellurium oxycompounds. *Mineralogical Magazine* **2016**, *80* (3), 415-545. DOI: 10.1180/minmag.2016.080.093.
(210) Chrysochoou, M.; Theologou, E.; Bompoti, N.; Dermatas, D.; Panagiotakis, I. Occurrence, Origin and Transformation Processes of Geogenic Chromium in Soils and Sediments. *Current Pollution Reports* **2016**, *2* (4), 224-235. DOI: 10.1007/s40726-016-0044-2.
(211) Chukhrov, F. V.; Gorshkov, A. I.; Rudnitskaya, E. S.; Beresovskaya, V. V.; Sivtsov, A. V. Manganese Minerals in Clays - a Review. *Clays and Clay Minerals* **1980**, *28* (5), 346-354. DOI: Doi 10.1346/Ccmn.1980.0280504.
(212) Chung, Y. H.; Lee, C. W. Electrochemical behaviors of Indium. *Journal of Electrochemical Science and Technology* **2012**, *3* (1), 1-13. DOI: 10.5229/Jecst.2012.3.1.1.
(213) Ciceri, D.; Manning, D. A.; Allanore, A. Historical and technical developments of potassium resources. *Sci Total Environ* **2015**, *502*, 590-601. DOI: 10.1016/j.scitotenv.2014.09.013.

(214) Clearwater, S. J.; Farag, A. M.; Meyer, J. S. Bioavailability and toxicity of dietborne copper and zinc to fish. *Comp Biochem Physiol C Toxicol Pharmacol* **2002**, *132* (3), 269-313. DOI: 10.1016/s1532-0456(02)00078-9.
(215) Cloke, F. G. N. "Zero oxidation state compounds of scandium, yttrium, and the lanthanides.". *Chemical Society Reviews* **1993**, *22*.
(216) Codd, R.; Irwin, J. A.; Lay, P. A. Sialoglycoprotein and carbohydrate complexes in chromium toxicity. *Curr Opin Chem Biol* **2003**, *7* (2), 213-219. DOI: 10.1016/s1367-5931(03)00017-6.
(217) Cohen, M. D.; Kargacin, B.; Klein, C. B.; Costa, M. Mechanisms of chromium carcinogenicity and toxicity. *Crit Rev Toxicol* **1993**, *23* (3), 255-281. DOI: 10.3109/10408449309105012.
(218) Cohen, S. M.; Chowdhury, A.; Arnold, L. L. Inorganic arsenic: A non-genotoxic carcinogen. *J Environ Sci (China)* **2016**, *49*, 28-37. DOI: 10.1016/j.jes.2016.04.015.
(219) Collery, P.; Keppler, B.; Madoulet, C.; Desoize, B. Gallium in cancer treatment. *Crit Rev Oncol Hematol* **2002**, *42* (3), 283-296. DOI: 10.1016/s1040-8428(01)00225-6.
(220) Collins, R. N.; Kinsela, A. S. The aqueous phase speciation and chemistry of cobalt in terrestrial environments. *Chemosphere* **2010**, *79* (8), 763-771. DOI: 10.1016/j.chemosphere.2010.03.003.
(221) Collins, R. N.; Kinsela, A. S. Pedogenic factors and measurements of the plant uptake of cobalt. *Plant and Soil* **2011**, *339* (1-2), 499-512. DOI: 10.1007/s11104-010-0584-y.
(222) Coman, V.; Robotin, B.; Ilea, P. Nickel recovery/removal from industrial wastes: A review. *Resour Conserv Recy* **2013**, *73*, 229-238. DOI: 10.1016/j.resconrec.2013.01.019.
(223) Copetti, D.; Finsterle, K.; Marziali, L.; Stefani, F.; Tartari, G.; Douglas, G.; Reitzel, K.; Spears, B. M.; Winfield, I. J.; Crosa, G.; D'Haese, P.; Yasseri, S.; Lurling, M. Eutrophication management in surface waters using lanthanum modified bentonite: A review. *Water Res* **2016**, *97*, 162-174. DOI: 10.1016/j.watres.2015.11.056.
(224) Corain, B.; Nicolini, M.; Zatta, P. Aspects of the bioinorganic chemistry of aluminium (III) relevant to the metal toxicity. *Coordination chemistry reviews* **1992**, *112*.
(225) Corin, K. C.; McFadzean, B. J.; Shackleton, N. J.; O'Connor, C. T. Challenges Related to the Processing of Fines in the Recovery of Platinum Group Minerals (PGMs). *Minerals* **2021**, *11* (5), 533. DOI: 10.3390/min11050533.
(226) Cornell, R. M. Adsorption of Cesium on Minerals - a Review. *Journal of Radioanalytical and Nuclear Chemistry-Articles* **1993**, *171* (2), 483-500. DOI: Doi 10.1007/Bf02219872.
(227) López Correa, M.; Montagna, P.; Joseph, N.; Ruggeberg, A.; Fietzke, J.; Flogel, S.; Dorschel, B.; Goldstein, S.; Wheeler, A.; Freiwald, A. Preboreal onset of cold-water coral growth beyond the Arctic Circle revealed by coupled radiocarbon and U-series dating and neodymium isotopes. *Quaternary Science Reviews* **2012**, *34*.
(228) Costa, H. P. D.; da Silva, M. G. C.; Vieira, M. G. A. Biosorption of aluminum ions from aqueous solutions using non-conventional low-cost materials: A review. *Journal of Water Process Engineering* **2021**, *40,* 101925. DOI: 10.1016/j.jwpe.2021.101925.
(229) Costa, M.; Klein, C. B. Toxicity and carcinogenicity of chromium compounds in humans. *Crit Rev Toxicol* **2006**, *36* (2), 155-163. DOI: 10.1080/10408440500534032.
(230) Cotton, S. A. Recent advances in the chemistry of scandium. *Polyhedron* **1999**, *18* (12), 1691-1715. DOI: Doi 10.1016/S0277-5387(99)00039-X.
(231) Cotton, S. A. The Scandium Aqua Ion Revisited. *Comments on Inorganic Chemistry* **2018**, *38* (3), 110-125. DOI: 10.1080/02603594.2018.1486303.

(232) Cox, A.; Venkatachalam, P.; Sahi, S.; Sharma, N. Silver and titanium dioxide nanoparticle toxicity in plants: A review of current research. *Plant Physiology and Biochemistry* **2016**, *107*, 147-163. DOI: 10.1016/j.plaphy.2016.05.022.
(233) Craft, E.; Abu-Qare, A.; Flaherty, M.; Garofolo, M.; Rincavage, H.; Abou-Donia, M. Depleted and natural uranium: chemistry and toxicological effects. *J Toxicol Environ Health B Crit Rev* **2004**, *7* (4), 297-317. DOI: 10.1080/10937400490452714.
(234) Crawley, R. A.; Sandstone uranium deposits in the United States: a review of the history, distribution, genesis, mining areas, and outlook. *Energy Exploration & Exploitation,* **1983**, *1(3).*
(235) Creus, J.; De Tovar, J.; Romero, N.; Garcia-Anton, J.; Philippot, K.; Bofill, R.; Sala, X. Ruthenium Nanoparticles for Catalytic Water Splitting. *ChemSusChem* **2019**, *12* (12), 2493-2514. DOI: 10.1002/cssc.201900393.
(236) Crognale, S.; Amalfitano, S.; Casentini, B.; Fazi, S.; Petruccioli, M.; Rossetti, S. Arsenic-related microorganisms in groundwater: a review on distribution, metabolic activities and potential use in arsenic removal processes. *Reviews in Environmental Science and Bio-Technology* **2017**, *16* (4), 647-665. DOI: 10.1007/s11157-017-9448-8.
(237) Cronan, C. S.; Grigal, D. F. Use of Calcium Aluminum Ratios as Indicators of Stress in Forest Ecosystems. *Journal of Environmental Quality* **1995**, *24* (2), 209-226. DOI: DOI 10.2134/jeq1995.00472425002400020002x.
(238) Cronin, S. J.; Manoharan, V.; Hedley, M. J.; Loganathan, P. Fluoride: A review of its fate, bioavailability, and risks of fluorosis in grazed-pasture systems in New Zealand. *New Zealand Journal of Agricultural Research* **2000**, *43(3)*, 295-321.
(239) Crossgrove, J.; Zheng, W. Manganese toxicity upon overexposure. *NMR in Biomedicine* **2004**, *17* (8), 544-553. DOI: 10.1002/nbm.931.
(240) Cui, J.; Jing, C. A review of arsenic interfacial geochemistry in groundwater and the role of organic matter. *Ecotoxicol Environ Saf* **2019**, *183*, 109550. DOI: 10.1016/j.ecoenv.2019.109550.
(241) Cui, J. J. State of the Art in Germanium-Containing Aromatic Systems. *Chinese Journal of Organic Chemistry* **2018**, *38* (11), 2888-2895. DOI: 10.6023/cjoc201805049.
(242) Cuney, M.; Kyser, K. Recent and not-so-recent developments in uranium deposits and implications for exploration. *Mineralogical Association of Canada Short Course Series* **2009**, Volume 39.
(243) Cuney, M. The extreme diversity of uranium deposits. *Mineralium Deposita* **2009**, *44* (1), 3-9. DOI: 10.1007/s00126-008-0223-1.
(244) Curti, E.; Degueldre, C. Solubility and hydrolysis of Zr oxides: a review and supplemental data. *Radiochimica Acta* **2002**, *90* (9-11), 801-804. DOI: DOI 10.1524/ract.2002.90.9-11_2002.801.
(245) Czajka, M.; Sawicki, K.; Sikorska, K.; Popek, S.; Kruszewski, M.; Kapka-Skrzypczak, L. Toxicity of titanium dioxide nanoparticles in central nervous system. *Toxicol In Vitro* **2015**, *29* (5), 1042-1052. DOI: 10.1016/j.tiv.2015.04.004.
(246) D'Haese, P. C.; Douglas, G.; Verhulst, A.; Neven, E.; Behets, G. J.; Vervaet, B. A.; Finsterle, K.; Lurling, M.; Spears, B. Human health risk associated with the management of phosphorus in freshwaters using lanthanum and aluminium. *Chemosphere* **2019**, *220*, 286-299. DOI: 10.1016/j.chemosphere.2018.12.093.
(247) Dadwal, A.; Mishra, V. Review on Biosorption of Arsenic From Contaminated Water. *Clean-Soil Air Water* **2017**, *45* (7), 1600364. DOI: 10.1002/clen.201600364.

(248) Dahle, J. T.; Arai, Y. Environmental geochemistry of cerium: applications and toxicology of cerium oxide nanoparticles. *Int J Environ Res Public Health* **2015**, *12* (2), 1253-1278. DOI: 10.3390/ijerph120201253.
(249) Dai, S. F.; Graham, I. T.; Ward, C. R. A review of anomalous rare earth elements and yttrium in coal. *International Journal of Coal Geology* **2016**, *159*, 82-95. DOI: 10.1016/j.coal.2016.04.005.
(250) Dalapati, G. K.; Sharma, H.; Guchhait, A.; Chakrabarty, N.; Bamola, P.; Liu, Q.; Saianand, G.; Krishna, A. M. S.; Mukhopadhyay, S.; Dey, A.; Wong, T. K. S.; Zhuk, S.; Ghosh, S.; Chakrabortty, S.; Mahata, C.; Biring, S.; Kumar, A.; Ribeiro, C. S.; Ramakrishna, S.; Chakraborty, A. K.; Krishnamurthy, S.; Sonar, P.; Sharma, M. Tin oxide for optoelectronic, photovoltaic and energy storage devices: a review. *Journal of Materials Chemistry A* **2021**, *9* (31), 16621-16684. DOI: 10.1039/d1ta01291f.
(251) Daltry, V. D. C.; Wilson, A. H. Review of platinum-group mineralogy: compositions and elemental associations of the PG-minerals and unidentified PGE-phases. *Mineralogy and Petrology* **1997**, *60* (3-4), 185-229. DOI: 10.1007/Bf01173709.
(252) Darab, J. G.; Smith, P. A. Chemistry of technetium and rhenium species during low-level radioactive waste vitrification. *Chemistry of Materials* **1996**, *8* (5), 1004-1021. DOI: 10.1021/cm950418+.
(253) Das, P.; Das, B; Dash., P. Chromite mining pollution, environmental impact, toxicity and phytoremediation: a review. *Environmental Chemistry Letters* **2021**, *19*.
(254) Das, A. K. Micellar effect on the kinetics and mechanism of chromium(VI) oxidation of organic substrates. *Coordination Chemistry Reviews* **2004**, *248* (1-2), 81-99. DOI: 10.1016/j.cct.2003.10.012.
(255) Das, A. P.; Sukla, L. B.; Pradhan, N.; Nayak, S. Manganese biomining: A review. *Bioresour Technol* **2011**, *102* (16), 7381-7387. DOI: 10.1016/j.biortech.2011.05.018.
(256) Das, S.; Dowding, J. M.; Klump, K. E.; McGinnis, J. F.; Self, W.; Seal, S. Cerium oxide nanoparticles: applications and prospects in nanomedicine. *Nanomedicine (Lond)* **2013**, *8* (9), 1483-1508. DOI: 10.2217/nnm.13.133.
(257) Das, T.; Sharma, A.; Talukder, G. Effects of lanthanum in cellular systems. A review. *Biol Trace Elem Res* **1988**, *18*, 201-228. DOI: 10.1007/BF02917504.
(258) Daus, B.; Hansen, H. R. Analysis of antimony species - lessons learnt from more than two decades of environmental research. *Environmental Chemistry* **2016**, *13* (6), 913-918. DOI: 10.1071/En16028.
(259) Davison, W. Iron and Manganese in Lakes. *Earth-Science Reviews* **1993**, *34* (2), 119-163. DOI: 10.1016/0012-8252(93)90029-7.
(260) Dayan, A. D.; Paine, A. J. Mechanisms of chromium toxicity, carcinogenicity and allergenicity: review of the literature from 1985 to 2000. *Hum Exp Toxicol* **2001**, *20* (9), 439-451. DOI: 10.1191/096032701682693062.
(261) De Corte, S.; Hennebel, T.; De Gusseme, B.; Verstraete, W.; Boon, N. Bio-palladium: from metal recovery to catalytic applications. *Microb Biotechnol* **2012**, *5* (1), 5-17. DOI: 10.1111/j.1751-7915.2011.00265.x.
(262) de Vries, W.; Lofts, S.; Tipping, E.; Meili, M.; Groenenberg, J. E.; Schutze, G. Impact of soil properties on critical concentrations of cadmium, lead, copper, zinc, and mercury in soil and soil solution in view of ecotoxicological effects. *Rev Environ Contam Toxicol* **2007**, *191*, 47-89. DOI: 10.1007/978-0-387-69163-3_3.

(263) Dehaine, Q.; Tijsseling, L. T.; Glass, H. J.; Tormanen, T.; Butcher, A. R. Geometallurgy of cobalt ores: A review. *Minerals Engineering* **2021**, *160,* 106656. DOI: 10.1016/j.mineng.2020.106656.
(264) Delaval, A.; Duffa, C.; Radakovitch, O. A review on cesium desorption at the freshwater-seawater interface. *Journal of Environmental Radioactivity* **2020**, *218,* 106255. DOI: 10.1016/j.jenvrad.2020.106255.
(265) Deng, R.; Chen, Y.; Deng, X.; Huang, Z.; Zhou, S.; Ren, B.; Jin, G.; Hursthouse, A. A Critical Review of Resistance and Oxidation Mechanisms of Sb-Oxidizing Bacteria for the Bioremediation of Sb(III) Pollution. *Front Microbiol* **2021**, *12*, 738596. DOI: 10.3389/fmicb.2021.738596.
(266) Deng, R. J.; Jin, C. S.; Ren, B. Z.; Hou, B. L.; Hursthouse, A. S. The Potential for the Treatment of Antimony-Containing Wastewater by Iron-Based Adsorbents. *Water* **2017**, *9* (10), 794. DOI: 10.3390/w9100794.
(267) Deng, T. H. B.; van der Ent, A.; Tang, Y. T.; Sterckeman, T.; Echevarria, G.; Morel, J. L.; Qiu, R. L. Nickel hyperaccumulation mechanisms: a review on the current state of knowledge. *Plant and Soil* **2018**, *423* (1-2), 1-11. DOI: 10.1007/s11104-017-3539-8.
(268) National Food Institute-Technical University of Denmark; Doulgeridou, A.; Amlund, H.; Sloth, J.J.; Hansen, M. Review of Potentially Toxic Rare Earth Elements, Thallium and Tellurium in Plant-based Foods. *EFSA Journal* **2020**, *18(S1), e181101.*
(269) DePaolo, D. J.; Daley, E. E. Neodymium isotopes in basalts of the southwest basin and range and lithospheric thinning during continental extension. *Chemical Geology* **2000**, *169* (1-2), 157-185. DOI: 10.1016/S0009-2541(00)00261-8.
(270) Deqian, L. A review on yttrium solvent extraction chemistry and separation process. *Journal of Rare Earths* **2017**, *35*.
(271) Dessemond, C.; Lajoie-Leroux, F.; Soucy, G.; Laroche, N.; Magnan, J. F. Spodumene: The Lithium Market, Resources and Processes. *Minerals* **2019**, *9* (6), 334. DOI: 10.3390/min9060334.
(272) Deval, G.; Boland, S.; Fournier, T.; Ferecatu, I. On Placental Toxicology Studies and Cerium Dioxide Nanoparticles. *Int J Mol Sci* **2021**, *22* (22). DOI: 10.3390/ijms222212266.
(273) Dhal, B.; Thatoi, H. N.; Das, N. N.; Pandey, B. D. Chemical and microbial remediation of hexavalent chromium from contaminated soil and mining/metallurgical solid waste: a review. *J Hazard Mater* **2013**, *250-251*, 272-291. DOI: 10.1016/j.jhazmat.2013.01.048.
(274) Dhall, A.; Self, W. Cerium Oxide Nanoparticles: A Brief Review of Their Synthesis Methods and Biomedical Applications. *Antioxidants* **2018**, *7* (8), 97. DOI: 10.3390/antiox7080097.
(275) Di, X.; Beesley, L.; Zhang, Z.; Zhi, S.; Jia, Y.; Ding, Y. Microbial Arsenic Methylation in Soil and Uptake and Metabolism of Methylated Arsenic in Plants: A Review. *Int J Environ Res Public Health* **2019**, *16* (24). DOI: 10.3390/ijerph16245012.
(276) Dimapilis, E. A. S.; Hsu, C. S.; Mendoza, R. M. O.; Lu, M. C. Zinc oxide nanoparticles for water disinfection. *Sustainable Environment Research* **2018**, *28* (2), 47-56. DOI: 10.1016/j.serj.2017.10.001.
(277) Ding, Y. R.; Zeng, M. Q.; Fu, L. Surface Chemistry of Gallium-Based Liquid Metals. *Matter* **2020**, *3* (5), 1477-1506. DOI: 10.1016/j.matt.2020.08.012.
(278) Domingo, J. L. Cobalt in the environment and its toxicological implications. *Rev Environ Contam Toxicol* **1989**, *108*, 105-132. DOI: 10.1007/978-1-4613-8850-0_3.

(279) Domingo, J. L. Vanadium: a review of the reproductive and developmental toxicity. *Reprod Toxicol* **1996**, *10* (3), 175-182. DOI: 10.1016/0890-6238(96)00019-6.
(280) Domingo, J. L. Reproductive and developmental toxicity of natural and depleted uranium: a review. *Reprod Toxicol* **2001**, *15* (6), 603-609. DOI: 10.1016/s0890-6238(01)00181-2.
(281) Domingo, J. L. Vanadium and tungsten derivatives as antidiabetic agents - A review of their toxic effects. *Biological Trace Element Research* **2002**, *88* (2), 97-112. DOI: 10.1385/Bter:88:2:097.
(282) Domingo, J. L.; Gomez, M. Vanadium compounds for the treatment of human diabetes mellitus: A scientific curiosity? A review of thirty years of research. *Food Chem Toxicol* **2016**, *95*, 137-141. DOI: 10.1016/j.fct.2016.07.005.
(283) Dong-sheng, M. Progress in research on geochemistry of tungsten. *Geological Journal of China Universities* **2009**, *15*.
(284) Tan, D.; Li, D.; Xiao, Y. Geochemical Characteristics of Niobium and Tantalum: A Review of Twin Elements. *Earth Science* **2018**, 43.
(285) Dorea, J. G. Neurotoxic effects of combined exposures to aluminum and mercury in early life (infancy). *Environ Res* **2020**, *188*, 109734. DOI: 10.1016/j.envres.2020.109734.
(286) Downs, A. J. *Chemistry of aluminium, gallium, indium and thallium*; Springer Science & Business Media, 1993.
(287) Drahota, P.; Filippi, M. Secondary arsenic minerals in the environment: a review. *Environ Int* **2009**, *35* (8), 1243-1255. DOI: 10.1016/j.envint.2009.07.004.
(288) Du, J.; Xu, S. D.; Zhou, Q. W.; Li, H. X.; Fu, L.; Tang, J. H.; Jin, M. Q. The ecotoxicology of titanium dioxide nanoparticles, an important engineering nanomaterial. *Toxicological and Environmental Chemistry* **2019**, *101* (3-6), 165-189. DOI: 10.1080/02772248.2019.1693572.
(289) Dubiella-Jackowska, A.; Polkowska, Z.; Namieńnik, J. Platinum group elements in the environment: emissions and exposure. *Reviews of Environmental Contamination and Toxicology Volume* **2008**.
(290) Dubiella-Jackowska, A.; Kudak, B.; Polkowska, Z.; Namisenik, J. Environmental Fate of Traffic-Derived Platinum Group Metals. *Critical Reviews in Analytical Chemistry* **2009**, *39* (4), 251-271.
(291) Dubiella-Jackowska, A.; Polkowska, Z.; Namiesnik, J. Platinum group elements: A challenge for environmental analytics. *Polish Journal of Environmental Studies* **2007**, *16* (3), 329-345.
(292) Duda-Chodak, A.; Blaszczyk, U. The Impact of Nickel on Human Health. *Journal of Elementology* **2008**, *13* (4), 685-696.
(293) Duker, A. A.; Carranza, E. J.; Hale, M. Arsenic geochemistry and health. *Environ Int* **2005**, *31* (5), 631-641. DOI: 10.1016/j.envint.2004.10.020.
(294) Dumas, A.; Couvreur, P. Palladium: a future key player in the nanomedical field? *Chem Sci* **2015**, *6* (4), 2153-2157. DOI: 10.1039/c5sc00070j.
(295) Durakovic, A. Medical effects of internal contamination with actinides: further controversy on depleted uranium and radioactive warfare. *Environmental Health and Preventive Medicine* **2016**, *21* (3), 111-117. DOI: 10.1007/s12199-016-0524-4.
(296) Durán, N.; Favaro, W. Biogenic synthesis of important environmental minerals: magnesium phosphate compounds and perspectives. *Química Nova* **2018**, *41*.
(297) Dusengemungu, L.; Kasali, G.; Gwanama, C.; Ouma, K. O. Recent Advances in Biosorption of Copper and Cobalt by Filamentous Fungi. *Front Microbiol* **2020**, *11*, 582016. DOI: 10.3389/fmicb.2020.582016.

(298) Economou-Eliopoulos, M. Platinum-group element distribution in chromite ores from ophiolite complexes: implications for their exploration. *Ore Geology Reviews* **1996**, *11*.
(299) Edahbi, M.; Plante, B.; Benzaazoua, M. Environmental challenges and identification of the knowledge gaps associated with REE mine wastes management. *J Clean Prod* **2019**, *212*, 1232-1241. DOI: 10.1016/j.jclepro.2018.11.228.
(300) Eggert, R.; Wadia, C.; Anderson, C.; Bauer, D.; Fields, F.; Meinert, L.; Taylor, P. Rare Earths: Market Disruption, Innovation, and Global Supply Chains. *Annual Review of Environment and Resources, Vol 41* **2016**, *41*, 199-222. DOI: 10.1146/annurev-environ-110615-085700.
(301) Ek, K. H.; Morrison, G. M.; Rauch, S. Environmental routes for platinum group elements to biological materials--a review. *Sci Total Environ* **2004**, *334-335*, 21-38. DOI: 10.1016/j.scitotenv.2004.04.027.
(302) el Bahri, L.; Ben Romdane, S. Arsenic poisoning in livestock. *Vet Hum Toxicol* **1991**, *33* (3), 259-264.
(303) El-Jaoual, T.; Cox, D. A. Manganese toxicity in plants. *Journal of Plant Nutrition* **1998**, *21* (2), 353-386. DOI: 10.1080/01904169809365409.
(304) Elahi, A.; Arooj, I.; Bukhari, D. A.; Rehman, A. Successive use of microorganisms to remove chromium from wastewater. *Appl Microbiol Biotechnol* **2020**, *104* (9), 3729-3743. DOI: 10.1007/s00253-020-10533-y.
(305) Elbashier, E.; Mussa, A.; Hafiz, M.; Hawari, A. H. Recovery of rare earth elements from waste streams using membrane processes: An overview. *Hydrometallurgy* **2021**, *204,* 105706. DOI: 10.1016/j.hydromet.2021.105706.
(306) Elkina, V.; Kurushkin, M. Promethium: To Strive, to Seek, to Find and Not to Yield. *Front Chem* **2020**, *8*, 588. DOI: 10.3389/fchem.2020.00588.
(307) Elliott, T.; Steele, R. C. J. The Isotope Geochemistry of Ni. *Non-Traditional Stable Isotopes* **2017**, *82*, 511-541. DOI: 10.2138/rmg.2017.82.12.
(308) Emsbo, P.; McLaughlin, P.; Breit, G.; duBray, E.; Koenig, A. Rare earth elements in sedimentary phosphate deposits: solution to the global REE crisis?. *Gondwana Research* **2015**, *27*.
(309) Ertani, A.; Mietto, A.; Borin, M.; Nardi, S. Chromium in Agricultural Soils and Crops: A Review. *Water Air and Soil Pollution* **2017**, *228* (5), 190. DOI: 10.1007/s11270-017-3356-y.
(310) Evangelou, A. M. Vanadium in cancer treatment. *Crit Rev Oncol Hematol* **2002**, *42* (3), 249-265. DOI: 10.1016/s1040-8428(01)00221-9.
(311) Faa, A.; Gerosa, C.; Fanni, D.; Floris, G.; Eyken, P. V.; Lachowicz, J. I.; Nurchi, V. M. Depleted Uranium and Human Health. *Curr Med Chem* **2018**, *25* (1), 49-64. DOI: 10.2174/0929867324666170426102343.
(312) Fang, G. Z.; Zhou, J.; Pan, A. Q.; Liang, S. Q. Recent Advances in Aqueous Zinc-Ion Batteries. *Acs Energy Letters* **2018**, *3* (10), 2480-2501. DOI: 10.1021/acsenergylett.8b01426.
(313) Farkasovska, I.; Zavadska, M.; Zemberyova, M. Determination and speciation of antimony in environmental samples by AAS techniques. *Chemicke Listy* **1999**, *93* (3), 173-180.
(314) Farooq, M. A.; Islam, F.; Ali, B.; Najeeb, U.; Mao, B. Z.; Gill, R. A.; Yan, G. J.; Siddique, K. H. M.; Zhou, W. J. Arsenic toxicity in plants: Cellular and molecular mechanisms of its transport and metabolism. *Environmental and Experimental Botany* **2016**, *132*, 42-52. DOI: 10.1016/j.envexpbot.2016.08.004.
(315) Faure, G.; Powell, J. The geochemistry of rubidium and strontium. In *Strontium isotope geology;* Springer, Berlin: Heidelberg **1972**.

(316) Feng, R. W.; Wei, C. Y.; Tu, S. X.; Ding, Y. Z.; Wang, R. G.; Guo, J. K. The uptake and detoxification of antimony by plants: A review. *Environmental and Experimental Botany* **2013**, *96*, 28-34. DOI: 10.1016/j.envexpbot.2013.08.006.
(317) Ferguson, J.; Gavis, J. A review of the arsenic cycle in natural waters. *Water research* **1972**, *6*.
(318) Fernandez-Ibanez, M. A.; Macia, B.; Alonso, D. A.; Pastor, I. M. Palladium and organocatalysis: an excellent recipe for asymmetric synthesis. *Molecules* **2013**, *18* (9), 10108-10121. DOI: 10.3390/molecules180910108.
(319) Fernando, D. R.; Lynch, J. P. Manganese phytotoxicity: new light on an old problem. *Ann Bot* **2015**, *116* (3), 313-319. DOI: 10.1093/aob/mcv111.
(320) Ferreira, L. M. R.; Cunha-Oliveira, T.; Sobral, M. C.; Abreu, P. L.; Alpoim, M. C.; Urbano, A. M. Impact of Carcinogenic Chromium on the Cellular Response to Proteotoxic Stress. *Int J Mol Sci* **2019**, *20* (19). DOI: 10.3390/ijms20194901.
(321) Filella, M. How reliable are environmental data on 'orphan'elements? The case of bismuth concentrations in surface waters. *Journal of Environmental Monitoring* **2010**, *12*.
(322) Filella, M. Tantalum in the environment. *Earth-Science Reviews* **2017**, *173*, 122-140. DOI: 10.1016/j.earscirev.2017.07.002.
(323) Filella, M.; Belzile, N.; Chen, Y. W. Antimony in the environment: a review focused on natural waters I. Occurrence. *Earth-Science Reviews* **2002**, *57* (1-2), 125-176. DOI: 10.1016/S0012-8252(01)00070-8.
(324) Filella, M.; Belzile, N.; Chen, Y. W. Antimony in the environment: a review focused on natural waters II. Relevant solution chemistry. *Earth-Science Reviews* **2002**, *59* (1-4), 265-285. DOI: 10.1016/S0012-8252(02)00089-2.
(325) Filella, M.; Belzile, N.; Chen, Y. W.; Elleouet, C.; May, P. M.; Mavrocordatos, D.; Nirel, P.; Porquet, A.; Quentel, F.; Silver, S. Antimony in aquatic systems. *Journal De Physique IV* **2003**, *107*, 475-478. DOI: 10.1051/jp4:20030344.
(326) Filella, M.; Belzile, N.; Lett, M. C. Antimony in the environment: A review focused on natural waters. III. Microbiota relevant interactions. *Earth-Science Reviews* **2007**, *80* (3-4), 195-217. DOI: 10.1016/j.earscirev.2006.09.003.
(327) Filella, M.; Hennebert, P.; Okkenhaug, G.; Turner, A. Occurrence and fate of antimony in plastics. *J Hazard Mater* **2020**, *390*, 121764. DOI: 10.1016/j.jhazmat.2019.121764.
(328) Filella, M.; May, P. M. Critical appraisal of available thermodynamic data for the complexation of antimony(III) and antimony(V) by low molecular mass organic ligands. *J Environ Monit* **2005**, *7* (12), 1226-1237. DOI: 10.1039/b511453e.
(329) Filella, M.; May, P. M. The aqueous solution thermodynamics of tantalum under conditions of environmental and biological interest. *Applied Geochemistry* **2019**, *109,* 104402. DOI: 10.1016/j.apgeochem.2019.104402.
(330) Filella, M.; May, P. M. The aqueous chemistry of tellurium: critically-selected equilibrium constants for the low-molecular-weight inorganic species. *Environmental Chemistry* **2019**, *16* (4), 289-295. DOI: 10.1071/En19017.
(331) Filella, M.; May, P. M. The aqueous solution thermodynamics of niobium under conditions of environmental and biological interest. *Applied Geochemistry* **2020**, *122,* 104729. DOI: 10.1016/j.apgeochem.2020.104729.
(332) Filella, M.; May, P. M. The aqueous solution chemistry of germanium under conditions of environmental and biological interest: Inorganic ligands. *Applied Geochemistry* **2023**, *155,* 105631. DOI: 10.1016/j.apgeochem.2023.105631.

(333) Filella, M.; Reimann, C.; Biver, M.; Rodushkin, I.; Rodushkina, K. Tellurium in the environment: current knowledge and identification of gaps. *Environmental Chemistry* **2019**, *16* (4), 215-228. DOI: 10.1071/En18229.
(334) Filella, M.; Williams, P. A. Antimony interactions with heterogeneous complexants in waters, sediments and soils: A review of binding data for homologous compounds. *Chemie Der Erde-Geochemistry* **2012**, *72*, 49-65. DOI: 10.1016/j.chemer.2012.01.006.
(335) Filella, M.; Williams, P. A.; Belzile, N. Antimony in the environment: knowns and unknowns. *Environmental Chemistry* **2009**, *6* (2), 95-105. DOI: 10.1071/En09007.
(336) Finch; Robert; Murakami., T. 3. Systematics and Paragenesis of Uranium Minerals. *Uranium* **2018**.
(337) Finley, J. W. Does environmental exposure to manganese pose a health risk to healthy adults? *Nutrition Reviews* **2004**, *62* (4), 148-153. DOI: 10.1301/nr.2004.apr.148-153.
(338) Finnegan, P. M.; Chen, W. Arsenic toxicity: the effects on plant metabolism. *Front Physiol* **2012**, *3*, 182. DOI: 10.3389/fphys.2012.00182.
(339) Fischer-Gödde, M.; Becker, H.; Wombacher, F. Rhodium, gold and other highly siderophile element abundances in chondritic meteorites. *Geochimica et Cosmochimica Acta* **2010**, *74*.
(340) Flamigni, L.; Barbieri, A.; Sabatini, C.; Ventura, B.; Barigelletti, F. Photochemistry and photophysics of coordination compounds: Iridium. *Photochemistry and Photophysics of Coordination Compounds Ii* **2007**, *281*, 143-203. DOI: 10.1007/128_2007_131.
(341) Fleet, A. Chapter 10 - Aqueous and sedimentary geochemistry of the rare earth elements. *Developments in Geochemistry,* Henderson, P. (Ed.) **1984**, *2*, 343-373.
(342) Flora, S. J. Arsenic-induced oxidative stress and its reversibility. *Free Radic Biol Med* **2011**, *51* (2), 257-281. DOI: 10.1016/j.freeradbiomed.2011.04.008.
(343) Flora, S. J. S.; Dasgupta, S. Toxicology of Gallium-Arsenide - an Appraisal. *Defence Science Journal* **1994**, *44* (1), 5-10. DOI: DOI 10.14429/dsj.44.4144.
(344) Flora, S. J. S.; Dwivedi, N. A Toxicochemical Review of Gallium Arsenide. *Defence Science Journal* **2012**, *62* (2), 95-104. DOI: 10.14429/dsj.62.1014.
(345) Forgacs, Z.; Massanyi, P.; Lukac, N.; Somosy, Z. Reproductive toxicology of nickel - Review. *Journal of Environmental Science and Health Part a-Toxic/Hazardous Substances & Environmental Engineering* **2012**, *47* (9), 1249-1260. DOI: 10.1080/10934529.2012.672114.
(346) Fortin, C.; Wang, F.; Pitre, D. Critical Review of Platinum Group Elements (Pd, Pt, Rh) in Aquatic Ecosystem **2011**.
(348) Fortoul, T. I.; Rodriguez-Lara, V.; Gonzalez-Villalva, A.; Rojas-Lemus, M.; Cano-Gutierrez, G.; Ustarroz-Cano, M.; Colin-Barenque, L.; Bizarro-Nevares, P.; Garcia-Pealez, I.; Montano, L. F.; Jimenez-Martinez, R. S.; Lopez-Valdez, N.; Ruiz-Guerrero, M. L.; Melendez-Garcia, N. A.; Garcia-Ibarra, F. A.; Martinez-Baez, V.; Alfaro, D. Z.; Muniz-Rivera-Cambas, A.; Lopez-Zepeda, L. S.; Quezada-Maldonado, E. M.; Cervantes-Yepez, S. Inhalation of vanadium pentoxide and its toxic effects in a mouse model. *Inorganica Chimica Acta* **2014**, *420*, 8-15. DOI: 10.1016/j.ica.2014.03.027.
(349) Frassinetti, S.; Bronzetti, G.; Caltavuturo, L.; Cini, M.; Croce, C. D. The role of zinc in life: a review. *J Environ Pathol Toxicol Oncol* **2006**, *25* (3), 597-610. DOI: 10.1615/jenvironpatholtoxicoloncol.v25.i3.40.
(350) Freda, J. The effects of aluminum and other metals on amphibians. *Environ Pollut* **1991**, *71* (2-4), 305-328. DOI: 10.1016/0269-7491(91)90035-u.

(351) Frenzel, M.; Hirsch, T.; Gutzmer, J. Gallium, germanium, indium, and other trace and minor elements in sphalerite as a function of deposit type - A meta-analysis. *Ore Geology Reviews* **2016**, *76*, 52-78. DOI: 10.1016/j.oregeorev.2015.12.017.
(352) Frenzel, M.; Ketris, M. P.; Gutzmer, J. On the geological availability of germanium. *Mineralium Deposita* **2014**, *49* (4), 471-486. DOI: 10.1007/s00126-013-0506-z.
(353) Fruchter; Jonathan. Peer reviewed: In-situ treatment of chromium-contaminated groundwater. *Environmental science & technology AA* **2002**, *36*.
(354) Fürstner, A. From understanding to prediction: Gold-and platinum-based π-acid catalysis for target oriented synthesis. *Accounts of chemical research* **2014**, *47*.
(355) Fuente-Martínez, D. l.; Manuel, J.; Herrera-Estrella., L. Advances in the understanding of aluminum toxicity and the development of aluminum-tolerant transgenic plants. *Advances in Agronomy* **1999**, *66*.
(356) Fuge, R. Fluorine in the environment, a review of its sources and geochemistry. *Applied Geochemistry* **2019**, *100*, 393-406. DOI: 10.1016/j.apgeochem.2018.12.016.
(357) Fuhrs, H.; Behrens, C.; Gallien, S.; Heintz, D.; Van Dorsselaer, A.; Braun, H. P.; Horst, W. J. Physiological and proteomic characterization of manganese sensitivity and tolerance in rice (Oryza sativa) in comparison with barley (Hordeum vulgare). *Ann Bot* **2010**, *105* (7), 1129-1140. DOI: 10.1093/aob/mcq046.
(358) Furstner, A. Gold and platinum catalysis--a convenient tool for generating molecular complexity. *Chem Soc Rev* **2009**, *38* (11), 3208-3221. DOI: 10.1039/b816696j.
(359) Gal, J.; Hursthouse, A.; Tatner, P.; Stewart, F.; Welton, R. Cobalt and secondary poisoning in the terrestrial food chain: data review and research gaps to support risk assessment. *Environ Int* **2008**, *34* (6), 821-838. DOI: 10.1016/j.envint.2007.10.006.
(360) Gao, F.; Olayiwola, A. U.; Liu, B.; Wang, S.; Du, H.; Li, J. Z.; Wang, X. D.; Chen, D. H.; Zhang, Y. Review of Vanadium Production Part I: Primary Resources. *Mineral Processing and Extractive Metallurgy Review* **2022**, *43* (4), 466-488. DOI: 10.1080/08827508.2021.1883013.
(361) Gao, N.; Huang, Z.; Liu, H.; Hou, J.; Liu, X. Advances on the toxicity of uranium to different organisms. *Chemosphere* **2019**, *237*, 124548. DOI: 10.1016/j.chemosphere.2019.124548.
(362) Gao, P.; Yang, Y. N.; Yin, Z.; Kang, F. X.; Fan, W. E.; Sheng, J. Y.; Feng, L.; Liu, Y. Z.; Du, Z. W.; Zhang, L. Q. A critical review on bismuth oxyhalide based photocatalysis for pharmaceutical active compounds degradation: Modifications, reactive sites, and challenges. *Journal of Hazardous Materials* **2021**, *412,* 125186. DOI: 10.1016/j.jhazmat.2021.125186.
(363) Garcia, J.; Liu, S. Z.; Louie, A. Y. Biological effects of MRI contrast agents: gadolinium retention, potential mechanisms and a role for phosphorus. *Philos Trans A Math Phys Eng Sci* **2017**, *375* (2107). DOI: 10.1098/rsta.2017.0180.
(364) Garg, N.; Singla, P. Arsenic toxicity in crop plants: physiological effects and tolerance mechanisms. *Environmental Chemistry Letters* **2011**, *9* (3), 303-321. DOI: 10.1007/s10311-011-0313-7.
(365) Gaustad, G.; Williams, E.; Leader, A. Rare earth metals from secondary sources: Review of potential supply from waste and byproducts. *Resources, Conservation and Recycling* **2020**, 167.
(366) Gauthier, P. T.; Blewett, T. A.; Garman, E. R.; Schlekat, C. E.; Middleton, E. T.; Suominen, E.; Cremazy, A. Environmental risk of nickel in aquatic Arctic ecosystems. *Sci Total Environ* **2021**, *797*, 148921. DOI: 10.1016/j.scitotenv.2021.148921.

(367) Gautneb, H.; Ronning, J. S.; Engvik, A. K.; Henderson, I. H. C.; Larsen, B. E.; Solberg, J. K.; Ofstad, F.; Gellein, J.; Elvebakk, H.; Davidsen, B. The Graphite Occurrences of Northern Norway, a Review of Geology, Geophysics, and Resources. *Minerals* **2020**, *10* (7), 626. DOI: 10.3390/min10070626.
(368) Gavrilescu, M.; Pavel, L. V.; Cretescu, I. Characterization and remediation of soils contaminated with uranium. *J Hazard Mater* **2009**, *163* (2-3), 475-510. DOI: 10.1016/j.jhazmat.2008.07.103.
(369) Gbaruko, B. C.; Ana, G. R. E. E.; Nwachukwu, J. K. Ecotoxicology of arsenic in the hydrosphere: Implications for public health. *African Journal of Biotechnology* **2008**, *7* (25), 4737-4742.
(370) Gebel, T. Toxicology of platinum, palladium, rhodium, and their compounds. *Anthropogenic Platinum Group Element Emissions;* Springer: Berlin, Heidelberg, **2000**.
(371) Gebel, T. Arsenic and antimony: comparative approach on mechanistic toxicology. *Chem Biol Interact* **1997**, *107* (3), 131-144. DOI: 10.1016/s0009-2797(97)00087-2.
(372) Genchi, G.; Carocci, A.; Lauria, G.; Sinicropi, M. S.; Catalano, A. Nickel: Human Health and Environmental Toxicology. *Int J Environ Res Public Health* **2020**, *17* (3). DOI: 10.3390/ijerph17030679.
(373) Genchi, G.; Lauria, G.; Catalano, A.; Carocci, A.; Sinicropi, M. S. The Double Face of Metals: The Intriguing Case of Chromium. *Applied Sciences-Basel* **2021**, *11* (2), 638. DOI: 10.3390/app11020638.
(374) Gensemer, R. W.; Playle, R. C. The bioavailability and toxicity of aluminum in aquatic environments. *Critical Reviews in Environmental Science and Technology* **1999**, *29* (4), 315-450. DOI: 10.1080/10643389991259245.
(375) Gerber, G. B.; Leonard, A.; Hantson, P. Carcinogenicity, mutagenicity and teratogenicity of manganese compounds. *Crit Rev Oncol Hematol* **2002**, *42* (1), 25-34. DOI: 10.1016/s1040-8428(01)00178-0.
(376) Ghosh; Sujita; Sharma, A.; Talukder., G. Zirconium. *Biological trace element research* **1992**, *35*.
(377) Ghosh, S.; Mohanty, S.; Akcil, A.; Sukla, L. B.; Das, A. P. A greener approach for resource recycling: Manganese bioleaching. *Chemosphere* **2016**, *154*, 628-639. DOI: 10.1016/j.chemosphere.2016.04.028.
(378) Giaccone, G. Clinical perspectives on platinum resistance. *Drugs* **2000**, *59 Suppl 4*, 9-17; discussion 37-18. DOI: 10.2165/00003495-200059004-00002.
(379) Giggenbach, W. F.; Sano, Y.; Wakita, H. Isotopic Composition of Helium, and Co2 and Ch4 Contents in Gases Produced Along the New-Zealand Part of a Convergent Plate Boundary. *Geochimica Et Cosmochimica Acta* **1993**, *57* (14), 3427-3455. DOI: 10.1016/0016-7037(93)90549-C.
(380) Gikas, P. Single and combined effects of nickel (Ni(II)) and cobalt (Co(II)) ions on activated sludge and on other aerobic microorganisms: a review. *J Hazard Mater* **2008**, *159* (2-3), 187-203. DOI: 10.1016/j.jhazmat.2008.02.048.
(381) Gilkes, R.; McKenzie, R. Geochemistry and mineralogy of manganese in soils. *Manganese in soils and plants;* Springer, Dordrecht, **1988**.
(382) Gilkes, R.; McKenzie, R. Geochemistry and mineralogy of manganese in soils. In *Manganese in Soils and Plants: Proceedings of the International Symposium on 'Manganese in Soils and Plants' held at the Waite Agricultural Research Institute, The University of*

Adelaide, Glen Osmond, South Australia, August 22–26, 1988 as an Australian Bicentennial Event, 1988; Springer: pp 23-35.
(383) Gilligan, R.; Nikoloski, A. N. The extraction of uranium from brannerite - A literature review. *Minerals Engineering* **2015**, *71*, 34-48. DOI: 10.1016/j.mineng.2014.10.007.
(384) Gilligan, R.; Nikoloski, A. N. The extraction of vanadium from titanomagnetites and other sources. *Minerals Engineering* **2020**, *146,* 106106. DOI: 10.1016/j.mineng.2019.106106.
(385) Ginzburg, A.; Ovchinnikov, L.; Solodov, N. Genetic types of tantalum ore deposits and their economic importance. *International Geology Review* **1972**, *14*.
(386) Gissi, F.; Stauber, J. L.; Binet, M. T.; Golding, L. A.; Adams, M. S.; Schlekat, C. E.; Garman, E. R.; Jolley, D. F. A review of nickel toxicity to marine and estuarine tropical biota with particular reference to the South East Asian and Melanesian region. *Environ Pollut* **2016**, *218*, 1308-1323. DOI: 10.1016/j.envpol.2016.08.089.
(387) Gitlin, M. Lithium and the kidney: an updated review. *Drug Saf* **1999**, *20* (3), 231-243. DOI: 10.2165/00002018-199920030-00004.
(388) Goldstein, S. J.; Jacobsen, S. B. Rare-Earth Elements in River Waters. *Earth and Planetary Science Letters* **1988**, *89* (1), 35-47. DOI: Doi 10.1016/0012-821x(88)90031-3.
(389) Golesorkhi, B.; Nozary, H.; Furstenberg, A.; Piguet, C. Erbium complexes as pioneers for implementing linear light-upconversion in molecules. *Materials Horizons* **2020**, *7* (5), 1279-1296. DOI: 10.1039/c9mh01899a.
(390) Golmohammadzadeh, R.; Faraji, F.; Rashchi, F. Recovery of lithium and cobalt from spent lithium ion batteries (LIBs) using organic acids as leaching reagents: A review. *Resour Conserv Recy* **2018**, *136*, 418-435. DOI: 10.1016/j.resconrec.2018.04.024.
(391) Gomez, V.; Callao, M. P. Chromium determination and speciation since 2000. *Trac-Trends in Analytical Chemistry* **2006**, *25* (10), 1006-1015. DOI: 10.1016/j.trac.2006.06.010.
(392) Gonnelli, C.; Renella, G. Chromium and nickel. *Heavy metals in soils: trace metals and metalloids in soils and their bioavailability* **2013**, 22.
(393) Gonzalez; Veronica. Environmental fate and ecotoxicity of lanthanides: are they a uniform group beyond chemistry?. *Environment international* **2014**, *71*.
(394) González; Yessica. Hydrometallurgical processing of magnesium minerals–A review. *Hydrometallurgy* **2021**.
(395) Gonzalez, N.; Esplugas, R.; Marques, M.; Domingo, J. L. Concentrations of arsenic and vanadium in environmental and biological samples collected in the neighborhood of petrochemical industries: A review of the scientific literature. *Sci Total Environ* **2021**, *771*, 145149. DOI: 10.1016/j.scitotenv.2021.145149.
(396) Goodman, J. E.; Prueitt, R. L.; Thakali, S.; Oller, A. R. The nickel ion bioavailability model of the carcinogenic potential of nickel-containing substances in the lung. *Crit Rev Toxicol* **2011**, *41* (2), 142-174. DOI: 10.3109/10408444.2010.531460.
(397) Gorny, J.; Billon, G.; Lesven, L.; Dumoulin, D.; Made, B.; Noiriel, C. Arsenic behavior in river sediments under redox gradient: a review. *Sci Total Environ* **2015**, *505*, 423-434. DOI: 10.1016/j.scitotenv.2014.10.011.
(398) Gorny, J.; Billon, G.; Noiriel, C.; Dumoulin, D.; Lesven, L.; Made, B. Chromium behavior in aquatic environments: a review. *Environmental Reviews* **2016**, *24* (4), 503-516. DOI: 10.1139/er-2016-0012.
(399) Gorostiza, S.; Sauri, D. Naturalizing pollution: a critical social science view on the link between potash mining and salinization in the Llobregat river basin, northeast Spain.

Philosophical Transactions of the Royal Society B-Biological Sciences **2019**, *374* (1764), 20180006. DOI: 10.1098/rstb.2018.0006.
(400) Gourcerol, B.; Gloaguen, E.; Melleton, J.; Tuduri, J.; Galiegue, X. Re-assessing the European lithium resource potential - A review of hard-rock resources and metallogeny. *Ore Geology Reviews* **2019**, *109*, 494-519. DOI: 10.1016/j.oregeorev.2019.04.015.
(401) Gräfe, M.; Power, G.; Klauber, C. Review of bauxite residue alkalinity and associated chemistry. *Clay Miner: Karawara,* WA, Australia **2009**.
(402) Gräfe, M.; Power, G.; Klauber, C. Review of bauxite residue alkalinity and associated chemistry. *Clay Miner. Karawara, WA, Australia* **2009**.
(403) Grew, E. S. Mineralogy, petrology and geochemistry of beryllium: An introduction and list of beryllium minerals. *Beryllium: Mineralogy, Petrology, and Geochemistry* **2002**, *50*, 1-76. DOI: DOI 10.2138/rmg.2202.50.01.
(404) Grew, E. S. Beryllium in metamorphic environments (emphasis on aluminous compositions). *Beryllium: Mineralogy, Petrology, and Geochemistry* **2002**, *50*, 487-549. DOI: DOI 10.2138/rmg.2002.50.12.
(405) Griffith, E. M.; Paytan, A. Barite in the ocean - occurrence, geochemistry and palaeoceanographic applications. *Sedimentology* **2012**, *59* (6), 1817-1835. DOI: 10.1111/j.1365-3091.2012.01327.x.
(406) Gritsenko, V. A.; Perevalov, T. V.; Islamov, D. R. Electronic properties of hafnium oxide: A contribution from defects and traps. *Physics Reports-Review Section of Physics Letters* **2016**, *613*, 1-20. DOI: 10.1016/j.physrep.2015.11.002.
(407) Gromada, J.; Mortreux, A.; Chenal, T.; Ziller, J. W.; Leising, F.; Carpentier, J. F. Neodymium alkoxides: Synthesis, characterization and their combinations with dialkylmagnesiums as unique systems for polymerization and block copolymerization of ethylene and methyl methacrylate. *Chemistry-a European Journal* **2002**, *8* (16), 3773-3788. DOI: 10.1002/1521-3765(20020816)8:16<3773::Aid-Chem3773>3.0.Co;2-S.
(408) Groshev, N. Y.; Rundkvist, T. V.; Karykowski, B. T.; Maier, W. D.; Korchagin, A. U.; Ivanov, A. N.; Junge, M. Low-Sulfide Platinum-Palladium Deposits of the Paleoproterozoic Fedorova-Pana Layered Complex, Kola Region, Russia. *Minerals* **2019**, *9* (12), 764. DOI: 10.3390/min9120764.
(409) Grosjean, C.; Miranda, P. H.; Perrin, M.; Poggi, P. Assessment of world lithium resources and consequences of their geographic distribution on the expected development of the electric vehicle industry. *Renewable & Sustainable Energy Reviews* **2012**, *16* (3), 1735-1744. DOI: 10.1016/j.rser.2011.11.023.
(410) Grzebisz, W.; Przygocka-Cyna, K.; Szczepaniak, W.; Diatta, J.; Potarzycki, J. Magnesium as a Nutritional Tool of Nitrogen Efficient Management - Plant Production and Environment. *Journal of Elementology* **2010**, *15* (4), 771-788.
(411) Gschwind, F.; Rodriguez-Garcia, G.; Sandbeck, D. J. S.; Gross, A.; Weil, M.; Fichtner, M.; Hormann, N. Fluoride ion batteries: Theoretical performance, safety, toxicity, and a combinatorial screening of new electrodes. *Journal of Fluorine Chemistry* **2016**, *182*, 76-90. DOI: 10.1016/j.jfluchem.2015.12.002.
(412) Gu, F.; Zhao, Z.; Wang, C.; Rao, H.; Zhao, B.; Liu, Z.; Bian, Z.; Huang, C. Lead-free tin-based perovskite solar cells: strategies toward high performance. *Solar RRL* **2019**.
(413) Gu, F. Q.; Zhang, Y. B.; Su, Z. J.; Tu, Y. K.; Liu, S.; Jiang, T. Recovery of chromium from chromium-bearing slags produced in the stainless-steel smelting: A review. *J Clean Prod* **2021**, *296, 126467*. DOI: 10.1016/j.jclepro.2021.126467.

(414) Guo, S.; Xiao, C.; Zhou, N.; Chi, R. Speciation, toxicity, microbial remediation and phytoremediation of soil chromium contamination. *Environmental Chemistry Letters* **2021**, *19, 1413-1431.*
(415) Guo, W. L.; Nazim, H.; Liang, Z. S.; Yang, D. F. Magnesium deficiency in plants: An urgent problem. *Crop Journal* **2016**, *4* (2), 83-91. DOI: 10.1016/j.cj.2015.11.003.
(416) Gupta, D. K.; Schulz, W.; Steinhauser, G.; Walther, C. Radiostrontium transport in plants and phytoremediation. *Environ Sci Pollut Res Int* **2018**, *25* (30), 29996-30008. DOI: 10.1007/s11356-018-3088-6.
(417) Gürel; Levent. Applications of the biosorption process for nickel removal from aqueous solutions–A review. *Chemical Engineering Communications* **2017**, *204*.
(418) Gurvich, L. V. "Thermodynamic properties of alkali metal hydroxides. Part II. Potassium, rubidium, and cesium hydroxides.". *Journal of Physical and Chemical Reference Data* **1997**, *26*.
(419) Gustafsson, J. P. Vanadium geochemistry in the biogeosphere -speciation, solid-solution interactions, and ecotoxicity. *Applied Geochemistry* **2019**, *102*, 1-25. DOI: 10.1016/j.apgeochem.2018.12.027.
(420) Gwenzi, W.; Mangori, L.; Danha, C.; Chaukura, N.; Dunjana, N.; Sanganyado, E. Sources, behaviour, and environmental and human health risks of high-technology rare earth elements as emerging contaminants. *Science of the Total Environment* **2018**, *636*, 299-313. DOI: 10.1016/j.scitotenv.2018.04.235.
(421) Gwiazda, R.; Lucchini, R.; Smith, D. Adequacy and consistency of animal studies to evaluate the neurotoxicity of chronic low-level manganese exposure in humans. *J Toxicol Environ Health A* **2007**, *70* (7), 594-605. DOI: 10.1080/10937400600882897.
(422) Hamasaki, T.; Nagase, H.; Yoshioka, Y.; Sato, T. Formation, Distribution, and Ecotoxicity of Methylmetals of Tin, Mercury, and Arsenic in the Environment. *Critical Reviews in Environmental Science and Technology* **1995**, *25* (1), 45-91. DOI: 10.1080/10643389509388474.
(423) Hamilton, E. I. The geobiochemistry of cobalt. *Sci Total Environ* **1994**, *150* (1-3), 7-39. DOI: 10.1016/0048-9697(94)90126-0.
(424) Hamilton, E. M.; Young, S. D.; Bailey, E. H.; Watts, M. J. Chromium speciation in foodstuffs: A review. *Food Chem* **2018**, *250*, 105-112. DOI: 10.1016/j.foodchem.2018.01.016.
(425) Han, Z. D.; Golev, A.; Edraki, M. A Review of Tungsten Resources and Potential Extraction from Mine Waste. *Minerals* **2021**, *11* (7), 701. DOI: 10.3390/min11070701.
(426) Handschuh-Wang, S.; Stadler, F. J.; Zhou, X. C. Critical Review on the Physical Properties of Gallium-Based Liquid Metals and Selected Pathways for Their Alteration. *Journal of Physical Chemistry C* **2021**, *125* (37), 20113-20142. DOI: 10.1021/acs.jpcc.1c05859.
(427) Hanley; J., J. "The aqueous geochemistry of the platinum-group elements (PGE) in surficial, low-T hydrothermal and high-T magmatic-hydrothermal environments.". *Exploration for Platinum group element deposits* **2005**, *35*.
(428) Hanor, J. S. Barite-celestine geochemistry and environments of formation. *Sulfate Minerals - Crystallography, Geochemistry and Environmental Significance* **2000**, *40*, 193-275. DOI: 10.2138/rmg.2000.40.4.
(429) Hansel, C. M. Manganese in Marine Microbiology. *Adv Microb Physiol* **2017**, *70*, 37-83. DOI: 10.1016/bs.ampbs.2017.01.005.
(430) Hanus-Fajerska, E.; Wiszniewska, A.; Kaminska, I. A Dual Role of Vanadium in Environmental Systems-Beneficial and Detrimental Effects on Terrestrial Plants and Humans. *Plants (Basel)* **2021**, *10* (6). DOI: 10.3390/plants10061110.

(431) Haque, N.; Hughes, A.; Lim, S.; Vernon, C. Rare Earth Elements: Overview of Mining, Mineralogy, Uses, Sustainability and Environmental Impact. *Resources-Basel* **2014**, *3* (4), 614-635. DOI: 10.3390/resources3040614.
(432) Harasim, P.; Filipek, T. Nickel in the Environment. *Journal of Elementology* **2015**, *20* (2), 525-534. DOI: 10.5601/jelem.2014.19.3.651.
(433) Hartwig, I.; de Oliveira, A. C.; de Carvalho, F. I. F.; Bertan, I.; da Silva, J. A. G.; Schmidt, D. A. M.; Valerio, I. P.; Maia, L. C.; Fonseca, D. A. R.; dos Reis, C. E. S. Associated mechanisms of aluminum tolerance in plants. *Semina-Ciencias Agrarias* **2007**, *28* (2), 219-228.
(434) Hassan, M. U.; Chattha, M. U.; Khan, I.; Chattha, M. B.; Aamer, M.; Nawaz, M.; Ali, A.; Khan, M. A. U.; Khan, T. A. Nickel toxicity in plants: reasons, toxic effects, tolerance mechanisms, and remediation possibilities-a review. *Environ Sci Pollut Res Int* **2019**, *26* (13), 12673-12688. DOI: 10.1007/s11356-019-04892-x.
(435) Hawthorne, F. C.; Huminicki, D. M. C. The crystal chemistry of beryllium. *Beryllium: Mineralogy, Petrology, and Geochemistry* **2002**, *50*, 333-403. DOI: DOI 10.2138/rmg.2002.50.9.
(436) Haynes, V. N.; Ward, J. E.; Russell, B. J.; Agrios, A. G. Photocatalytic effects of titanium dioxide nanoparticles on aquatic organisms-Current knowledge and suggestions for future research. *Aquat Toxicol* **2017**, *185*, 138-148. DOI: 10.1016/j.aquatox.2017.02.012.
(437) He, C. W.; Gu, L. P.; Xu, Z. X.; He, H.; Fu, G.; Han, F. X.; Huang, B.; Pan, X. J. Cleaning chromium pollution in aquatic environments by bioremediation, photocatalytic remediation, electrochemical remediation and coupled remediation systems. *Environmental Chemistry Letters* **2020**, *18* (3), 561-576. DOI: 10.1007/s10311-019-00960-3.
(438) He, H.; He, L.; Gu, M. Role of microRNAs in aluminum stress in plants. *Plant Cell Rep* **2014**, *33* (6), 831-836. DOI: 10.1007/s00299-014-1565-z.
(439) He, H.; Li, Y.; He, L. F. Aluminum toxicity and tolerance in Solanaceae plants. *South African Journal of Botany* **2019**, *123*, 23-29. DOI: 10.1016/j.sajb.2019.02.008.
(440) He, H.; Li, Y.; He, L. F. Role of nitric oxide and hydrogen sulfide in plant aluminum tolerance. *Biometals* **2019**, *32* (1), 1-9. DOI: 10.1007/s10534-018-0156-9.
(441) He, M.; Wang, N.; Long, X.; Zhang, C.; Ma, C.; Zhong, Q.; Wang, A.; Wang, Y.; Pervaiz, A.; Shan, J. Antimony speciation in the environment: Recent advances in understanding the biogeochemical processes and ecological effects. *J Environ Sci (China)* **2019**, *75*, 14-39. DOI: 10.1016/j.jes.2018.05.023.
(442) Heidari, M. Plant responses to arsenic: uptake, metabolism and resistance to arsenic toxicity - a review. *Zemdirbyste-Agriculture* **2017**, *104* (4), 369-376. DOI: 10.13080/z-a.2017.104.047.
(443) Heilgeist, S.; Sekine, R.; Sahin, O.; Stewart, R. A. Finding Nano: Challenges Involved in Monitoring the Presence and Fate of Engineered Titanium Dioxide Nanoparticles in Aquatic Environments. *Water* **2021**, *13* (5), 734. DOI: 10.3390/w13050734.
(444) Helmer, E. H.; Urban, N. R.; Eisenreich, S. J. Aluminum Geochemistry in Peatland Waters. *Biogeochemistry* **1990**, *9* (3), 247-276.
(445) Herath, I.; Vithanage, M.; Bundschuh, J. Antimony as a global dilemma: Geochemistry, mobility, fate and transport. *Environmental Pollution* **2017**, *223*, 545-559. DOI: 10.1016/j.envpol.2017.01.057.
(446) Herrmann, H.; Nolde, J.; Berger, S.; Heise, S. Aquatic ecotoxicity of lanthanum - A review and an attempt to derive water and sediment quality criteria. *Ecotoxicology and Environmental Safety* **2016**, *124*, 213-238. DOI: 10.1016/j.ecoenv.2015.09.033.

(447) Herrmann, W. A.; Anwander, R.; Kleine, M.; Scherer, W. Complexes of the Lanthanides .1. Solvent-Free Alkoxide Complexes of Neodymium and Dysprosium - Crystal and Molecular-Structure of Trans-Bis(Acetonitrile)Tris(Tri-Tert-Butylmethoxy)Neodymium. *Chemische Berichte-Recueil* **1992**, *125* (9), 1971-1979. DOI: DOI 10.1002/cber.19921250902.
(448) Hettick, B. E.; Canas-Carrell, J. E.; French, A. D.; Klein, D. M. Arsenic: A Review of the Element's Toxicity, Plant Interactions, and Potential Methods of Remediation. *J Agric Food Chem* **2015**, *63* (32), 7097-7107. DOI: 10.1021/acs.jafc.5b02487.
(449) Hille, R. Molybdenum and tungsten in biology. *Trends Biochem Sci* **2002**, *27* (7), 360-367. DOI: 10.1016/s0968-0004(02)02107-2.
(450) Hiradate; Syuntaro. Speciation of aluminum in soil environments: application of NMR technique. *Soil science and plant nutrition* **2004**, *50*.
(451) Hirano, S.; Suzuki, K. T. Exposure, metabolism, and toxicity of rare earths and related compounds. *Environ Health Perspect* **1996**, *104 Suppl 1* (Suppl 1), 85-95. DOI: 10.1289/ehp.96104s185.
(452) Hitzman, M. W.; Reynolds, N. A.; Sangster, D. F.; Allen, C. R.; Carman, C. E. Classification, genesis, and exploration guides for nonsulfide zinc deposits. *Economic Geology and the Bulletin of the Society of Economic Geologists* **2003**, *98* (4), 685-714. DOI: 10.2113/98.4.685.
(453) Hoatson, D. M.; Jaireth, S.; Jaques, A. L. Nickel sulfide deposits in Australia: Characteristics, resources, and potential. *Ore Geology Reviews* **2006**, *29* (3-4), 177-241. DOI: 10.1016/j.oregeorev.2006.05.002.
(454) Hodson; J., M.; Evans., D. E. Aluminium/silicon interactions in higher plants. *Journal of Experimental Botany* **1995**, *46*.
(455) Höll; R.; Kling, M.; Schroll., E. Metallogenesis of germanium—A review. *Ore Geology Reviews* **2007**, *30*.
(456) Holland, S. L.; Avery, S. V. Chromate toxicity and the role of sulfur. *Metallomics* **2011**, *3* (11), 1119-1123. DOI: 10.1039/c1mt00059d.
(457) Hon, Z.; Osterreicher, J.; Navratil, L. Depleted Uranium and Its Effects on Humans. *Sustainability* **2015**, *7* (4), 4063-4077. DOI: 10.3390/su7044063.
(458) Horovitz, C.T., Ed. *Scandium its occurrence, chemistry physics, metallurgy, biology and technology*. Academic Press: London, **1975**.
(459) Hossain, M. K.; Chanda, R.; El-Denglawey, A.; Emrose, T.; Rahman, M. T.; Biswas, M. C.; Hashizume, K. Recent progress of barium zirconate proton conductor in electrochemical hydrogen devices: A review. *Ceramics International* **2021**, *47(17)*, 23725-23748.
(460) Hou, J.; Wang, L.; Wang, C.; Zhang, S.; Liu, H.; Li, S.; Wang, X. Toxicity and mechanisms of action of titanium dioxide nanoparticles in living organisms. *J Environ Sci (China)* **2019**, *75*, 40-53. DOI: 10.1016/j.jes.2018.06.010.
(461) Hou, J.; Wu, Y.; Li, X.; Wei, B.; Li, S.; Wang, X. Toxic effects of different types of zinc oxide nanoparticles on algae, plants, invertebrates, vertebrates and microorganisms. *Chemosphere* **2018**, *193*, 852-860. DOI: 10.1016/j.chemosphere.2017.11.077.
(462) Housecroft, C. Scandium. *Coordination chemistry reviews* **1993**, *124*.
(463) Hoz, S. Samarium Iodide Showcase: Unraveling the Mechanistic Puzzle. *Acc Chem Res* **2020**, *53* (11), 2680-2691. DOI: 10.1021/acs.accounts.0c00497.
(464) Hu, X. M.; You, S. J.; Li, F.; Liu, Y. B. Recent advances in antimony removal using carbon-based nanomaterials: A review. *Frontiers of Environmental Science & Engineering* **2022**, *16* (4), 48. DOI: 10.1007/s11783-021-1482-7.

(465) Hu, Y.; Li, J.; Lou, B.; Wu, R.; Wang, G.; Lu, C.; Wang, H.; Pi, J.; Xu, Y. The Role of Reactive Oxygen Species in Arsenic Toxicity. *Biomolecules* **2020**, *10* (2). DOI: 10.3390/biom10020240.
(466) Hu, Z. Y.; Richter, H.; Sparovek, G.; Schnug, E. Physiological and biochemical effects of rare earth elements on plants and their agricultural significance: A review. *Journal of Plant Nutrition* **2004**, *27* (1), 183-220. DOI: 10.1081/Pln-120027555.
(467) Huang, B.; Pan, Z. F.; Su, X. Y.; An, L. Recycling of lithium-ion batteries: Recent advances and perspectives. *Journal of Power Sources* **2018**, *399*, 274-286. DOI: 10.1016/j.jpowsour.2018.07.116.
(468) Huang, B.; Pan, Z. F.; Su, X. Y.; An, L. Tin-based materials as versatile anodes for alkali (earth)-ion batteries. *Journal of Power Sources* **2018**, *395*, 41-59. DOI: 10.1016/j.jpowsour.2018.05.063.
(469) Huang, J. H. Impact of Microorganisms on Arsenic Biogeochemistry: A Review. *Water Air and Soil Pollution* **2014**, *225* (2), 1848. DOI: 10.1007/s11270-013-1848-y.
(470) Huang, J. H.; Huang, F.; Evans, L.; Glasauer, S. Vanadium: Global (bio)geochemistry. *Chemical Geology* **2015**, *417*, 68-89. DOI: 10.1016/j.chemgeo.2015.09.019.
(471) Huang, J. Z.; Zhang, H. C. Redox reactions of iron and manganese oxides in complex systems. *Frontiers of Environmental Science & Engineering* **2020**, *14* (5), 76. DOI: 10.1007/s11783-020-1255-8.
(472) Huang, S.; Zhu, J.; Tian, J.; Niu, Z. Recent Progress in the Electrolytes of Aqueous Zinc-Ion Batteries. *Chemistry* **2019**, *25* (64), 14480-14494. DOI: 10.1002/chem.201902660.
(473) Huaqing, L.; Shuisheng, X. Research on development of dolomite-ferrosilicon thermal reduction process of magnesium production. *Journal of rare earths* **2005**, *23*.
(474) Huda, N.; Khanom, A.; Rahman, M. M.; Huq, M. A.; Rahman, M. M.; Banu, N. A. Biochemical process and functional genes of arsenic accumulation in bioremediation: agricultural soil. *International Journal of Environmental Science and Technology* **2022**, *19* (9), 9189-9208. DOI: 10.1007/s13762-021-03655-x.
(475) Hudson-Edwards, K. A.; Santini, J. M. Arsenic-Microbe-Mineral Interactions in Mining-Affected Environments. *Minerals* **2013**, *3* (4), 337-351. DOI: 10.3390/min3040337.
(476) Hughes, K.; Meek, M. E.; Chan, P. K. L.; Shedden, J.; Bartlett, S.; Seed, L. J. Nickel and Its Compounds - Evaluation of Risks to Health from Environmental Exposure in Canada. *Journal of Environmental Science and Health Part C-Environmental Carcinogenesis & Ecotoxicology Reviews* **1994**, *12* (2), 417-433. DOI: 10.1080/10590509409373457.
(477) Humayun; Muhammad. "Perovskite-type lanthanum ferrite based photocatalysts: preparation, properties, and applications.". *Journal of Energy Chemistry* **2022**, *66*.
(478) Hummel, W.; Curti, E. Nickel aqueous speciation and solubility at ambient conditions: A thermodynamic elegy. *Monatshefte Fur Chemie* **2003**, *134* (7), 941-973. DOI: 10.1007/s00706-003-0010-8.
(479) Idee, J. M.; Fretellier, N.; Robic, C.; Corot, C. The role of gadolinium chelates in the mechanism of nephrogenic systemic fibrosis: A critical update. *Crit Rev Toxicol* **2014**, *44* (10), 895-913. DOI: 10.3109/10408444.2014.955568.
(480) Iftekhar, S.; Ramasamy, D. L.; Srivastava, V.; Asif, M. B.; Sillanpaa, M. Understanding the factors affecting the adsorption of Lanthanum using different adsorbents: A critical review. *Chemosphere* **2018**, *204*, 413-430. DOI: 10.1016/j.chemosphere.2018.04.053.

(481) Imtiaz, M.; Rizwan, M. S.; Xiong, S.; Li, H.; Ashraf, M.; Shahzad, S. M.; Shahzad, M.; Rizwan, M.; Tu, S. Vanadium, recent advancements and research prospects: A review. *Environ Int* **2015**, *80*, 79-88. DOI: 10.1016/j.envint.2015.03.018.
(482) Indelli, M. T.; Chiorboli, C.; Scandola, F. Photochemistry and photophysics of coordination compounds: Rhodium. *Photochemistry and Photophysics of Coordination Compounds I* **2007**, *280*, 215-255. DOI: 10.1007/128_2007_137.
(483) Innocenzi, V.; De Michelis, I.; Kopacek, B.; Veglio, F. Yttrium recovery from primary and secondary sources: a review of main hydrometallurgical processes. *Waste Manag* **2014**, *34* (7), 1237-1250. DOI: 10.1016/j.wasman.2014.02.010.
(484) Inostroza-Blancheteau, C.; Soto, B.; Ibanez, C.; Ulloa, P.; Aquea, F.; Arce-Johnson, P.; Reyes-Diaz, M. Mapping aluminum tolerance loci in cereals: A tool available for crop breeding. *Electronic Journal of Biotechnology* **2010**, *13* (4), 4. DOI: 10.2225/vol13-issue4-fulltext-4.
(485) Iqbal, S.; Jabeen, F.; Chaudhry, A. S.; Shah, M. A.; Batiha, G. E. Toxicity assessment of metallic nickel nanoparticles in various biological models: An interplay of reactive oxygen species, oxidative stress, and apoptosis. *Toxicology and Industrial Health* **2021**, *37* (10), 635-651. DOI:10.1177/07482337211011008.
(486) Iravani, S.; Varma, R. S. Sustainable synthesis of cobalt and cobalt oxide nanoparticles and their catalytic and biomedical applications. *Green Chemistry* **2020**, *22* (9), 2643-2661. DOI: 10.1039/d0gc00885k.
(487) Iyare, P. U. The effects of manganese exposure from drinking water on school-age children: A systematic review. *Neurotoxicology* **2019**, *73*, 1-7. DOI: 10.1016/j.neuro.2019.02.013.
(488) Jablonska-Czapla, M. Arsenic, Antimony, Chromium, and Thallium Speciation in Water and Sediment Samples with the LC-ICP-MS Technique. *International Journal of Analytical Chemistry* **2015**, *2015,* 171478. DOI: 10.1155/2015/171478.
(489) Jackson, M.; Hart, S.; Saal, A.; Shimizu, N.; Kurz, M.; Blusztajn, J.; Skovgaard, A. Globally elevated titanium, tantalum, and niobium (TITAN) in ocean island basalts with high 3He/4He. *Geochemistry, Geophysics, Geosystems* **2008**, 9.
(490) Jain, C. K.; Ali, I. Arsenic: Occurrence, toxicity and speciation techniques. *Water Research* **2000**, *34* (17), 4304-4312. DOI: 10.1016/S0043-1354(00)00182-2.
(491) Jain, J. K.; Nidhi, G. Arsenic Toxicity in Potable Water: Hazards and Management. *Research Journal of Chemistry and Environment* **2011**, *15* (1), 97-103.
(492) Jang; C., Y.; Somanna, Y.; Kim., H. J. I. J. "Source, distribution, toxicity and remediation of arsenic in the environment–a review.". *Int J Appl Environ Sci* **2016**, *11*.
(493) Jansen, S.; Broadley, M. R.; Robbrecht, E.; Smets, E. Aluminum hyperaccumulation in angiosperms: A review of its phylogenetic significance. *Botanical Review* **2002**, *68* (2), 235-269. DOI: 10.1663/0006-8101(2002)068[0235:Ahiaar]2.0.Co;2.
(494) Jara, A. D.; Betemariam, A.; Woldetinsae, G.; Kim, J. Y. Purification, application and current market trend of natural graphite: A review. *International Journal of Mining Science and Technology* **2019**, *29* (5), 671-689. DOI: 10.1016/j.ijmst.2019.04.003.
(495) Jasiński, A. W. The formation of copper-bismuth sulphosalts in sedimentary copper deposits: The Fore-Sudetic Copper Deposit, Poland as an example. *Ore Geology Reviews* **1994**, *9(3)*, 209-217.
(496) Jena, S. A Review on Potash Recovery from Different Rock and Mineral Sources. *Mining, Metallurgy & Exploration* **2021**, *38*.

(497) Jewett, S. A.; Ivanisevic, A. Wet-chemical passivation of InAs: toward surfaces with high stability and low toxicity. *Acc Chem Res* **2012**, *45* (9), 1451-1459. DOI: 10.1021/ar200282f.
(498) Jiang, Y.; Zheng, W. Cardiovascular toxicities upon managanese exposure. *Cardiovascular toxicology* **2005**, *5(4)*.
(499) Jiang, S. P. Development of lanthanum strontium cobalt ferrite perovskite electrodes of solid oxide fuel cells - A review. *International Journal of Hydrogen Energy* **2019**, *44* (14), 7448-7493. DOI: 10.1016/j.ijhydene.2019.01.212.
(500) Jin, X. L.; Ye, L. Q.; Xie, H. Q.; Chen, G. Bismuth-rich bismuth oxyhalides for environmental and energy photocatalysis. *Coordination Chemistry Reviews* **2017**, *349*, 84-101. DOI: 10.1016/j.ccr.2017.08.010.
(501) Jin, Z. X.; Zhang, Z.; Xiu, J. W.; Song, H. S.; Gatti, T.; He, Z. B. A critical review on bismuth and antimony halide based perovskites and their derivatives for photovoltaic applications: recent advances and challenges. *Journal of Materials Chemistry A* **2020**, *8* (32), 16166-16188. DOI: 10.1039/d0ta05433j.
(502) Jing, C.; Li, Y. L.; Landsberger, S. Review of soluble uranium removal by nanoscale zero valent iron. *J Environ Radioact* **2016**, *164*, 65-72. DOI: 10.1016/j.jenvrad.2016.06.027.
(503) Jobby, R.; Jha, P.; Yadav, A. K.; Desai, N. Biosorption and biotransformation of hexavalent chromium [Cr(VI)]: A comprehensive review. *Chemosphere* **2018**, *207*, 255-266. DOI: 10.1016/j.chemosphere.2018.05.050.
(504) Johnston, N. R.; Strobel, S. A. Principles of fluoride toxicity and the cellular response: a review. *Arch Toxicol* **2020**, *94* (4), 1051-1069. DOI: 10.1007/s00204-020-02687-5.
(505) Jokinen, R. Magnesium in the Environment. *Metal Ions in Biological Systems* **1990**, *26*, 15-32.
(506) Joksic, A. S.; Katz, S. A. Chelation therapy for treatment of systemic intoxication with uranium: A review. *Journal of Environmental Science and Health Part a-Toxic/Hazardous Substances & Environmental Engineering* **2015**, *50* (14), 1479-1488. DOI: 10.1080/10934529.2015.1071154.
(507) Jovanovic, B. Critical Review of Public Health Regulations of Titanium Dioxide, a Human Food Additive. *Integrated Environmental Assessment and Management* **2015**, *11* (1), 10-20. DOI: 10.1002/ieam.1571.
(508) Junaid, M.; Hashmi, M. Z.; Malik, R. N.; Pei, D. S. Toxicity and oxidative stress induced by chromium in workers exposed from different occupational settings around the globe: A review. *Environ Sci Pollut Res Int* **2016**, *23* (20), 20151-20167. DOI: 10.1007/s11356-016-7463-x.
(509) Jung, Y.; Lippard, S. J. Direct cellular responses to platinum-induced DNA damage. *Chem Rev* **2007**, *107* (5), 1387-1407. DOI: 10.1021/cr068207j.
(510) Botelho Junior, A.; Espinosa, D.; Vaughan, J.; Tenorio, J. Recovery of scandium from various sources: A critical review of the state of the art and future prospects. *Minerals Engineering* **2021**, *172*.
(511) Jyothi, N.; Farook, N.; Cho, M., Shim, J. Analysis and speciation of chromium in environmental matrices by various analytical techniques-a review. *Asian Journal of Chemistry* **2013**, *25*.
(512) Jyothi, R. K.; Lee, J. Y.; Kim, J. S.; Sohn, J. S. Liquid-liquid extraction of platinum from acidic solutions - A review. *Solvent Extraction Research and Development-Japan* **2009**, *16*, 13-22.

(513) Kalin, M.; Wheeler, W. N.; Meinrath, G. The removal of uranium from mining waste water using algal/microbial biomass. *J Environ Radioact* **2005**, *78* (2), 151-177. DOI: 10.1016/j.jenvrad.2004.05.002.
(514) Kamber, B. S.; Collerson, K. D. Origin of ocean island basalts: A new model based on lead and helium isotope systematics. *Journal of Geophysical Research-Solid Earth* **1999**, *104* (B11), 25479-25491. DOI: 10.1029/1999jb000258.
(515) Kan, X.; Dong, Y.; Feng, L.; Zhou, M.; Hou, H. Contamination and health risk assessment of heavy metals in China's lead-zinc mine tailings: A meta-analysis. *Chemosphere* **2021**, *267*, 128909. DOI: 10.1016/j.chemosphere.2020.128909.
(516) Kane, J. J.; Contescu, C. I.; Smith, R. E.; Strydom, G.; Windes, W. E. Understanding the reaction of nuclear graphite with molecular oxygen: Kinetics, transport, and structural evolution. *Journal of Nuclear Materials* **2017**, *493*, 343-367. DOI: 10.1016/j.jnucmat.2017.06.001.
(517) Kanmani, P.; Aravind, J.; Preston, D. Remediation of chromium contaminants using bacteria. *International Journal of Environmental Science and Technology* **2012**, *9* (1), 183-193. DOI: 10.1007/s13762-011-0013-7.
(518) Kanu, S. A.; Okonkwo, J. O.; Dakora, F. D. Aspalathus linearis (Rooibos tea) as potential phytoremediation agent: a review on tolerance mechanisms for aluminum uptake. *Environmental Reviews* **2013**, *21* (2), 85-92. DOI: 10.1139/er-2012-0055.
(519) Karim, S.; Ting, Y. P. Recycling pathways for platinum group metals from spent automotive catalyst: A review on conventional approaches and bio-processes. *Resour Conserv Recy* **2021**, *170*, 105588. DOI: 10.1016/j.resconrec.2021.105588.
(520) Kasprzak, K.; Sunderman Junior, F.; Salnikow, K. Nickel carcinogenesis. *Mutation Research/Fundamen tal and Molecular Mechanisms of Mutagenesis* **2003**, 533.
(521) Kaste, J. M.; Norton, S. A.; Hess, C. T. Environmental chemistry of beryllium-7. *Beryllium: Mineralogy, Petrology, and Geochemistry* **2002**, *50*, 271-289. DOI: 10.2138/rmg.2002.50.6.
(522) Kato, M.; Ohgami, N.; Ohnuma, S.; Hashimoto, K.; Tazaki, A.; Xu, H.; Kondo-Ida, L.; Yuan, T.; Tsuchiyama, T.; He, T.; Kurniasari, F.; Gu, Y.; Chen, W.; Deng, Y.; Komuro, K.; Tong, K.; Yajima, I. Multidisciplinary approach to assess the toxicities of arsenic and barium in drinking water. *Environ Health Prev Med* **2020**, *25* (1), 16. DOI: 10.1186/s12199-020-00855-8.
(523) Katoh, K.; Isshiki, H.; Komeda, T.; Yamashita, M. Molecular spintronics based on single-molecule magnets composed of multiple-decker phthalocyaninato terbium(III) complex. *Chem Asian J* **2012**, *7* (6), 1154-1169. DOI: 10.1002/asia.201100992.
(524) Katz, S. A.; Salem, H. The toxicology of chromium with respect to its chemical speciation: a review. *J Appl Toxicol* **1993**, *13* (3), 217-224. DOI: 10.1002/jat.2550130314.
(525) Kaur, H.; Garg, N. Zinc toxicity in plants: a review. *Planta* **2021**, *253* (6), 129. DOI: 10.1007/s00425-021-03642-z.
(526) Kaur, S.; Kamli, M. R.; Ali, A. Role of arsenic and its resistance in nature. *Can J Microbiol* **2011**, *57* (10), 769-774. DOI: 10.1139/w11-062.
(527) Kausar, A.; Bhatti, H. N. Adsorptive Removal of Uranium from Wastewater: A Review. *Journal of the Chemical Society of Pakistan* **2013**, *35* (3), 1041-1052.
(528) Kavanagh, L.; Keohane, J.; Cabellos, G. G.; Lloyd, A.; Cleary, J. Global Lithium Sources-Industrial Use and Future in the Electric Vehicle Industry: A Review. *Resources-Basel* **2018**, *7* (3), 57. DOI: 10.3390/resources7030057.

(529) Kawi, S.; Kathiraser, Y.; Ni, J.; Oemar, U.; Li, Z.; Saw, E. T. Progress in Synthesis of Highly Active and Stable Nickel-Based Catalysts for Carbon Dioxide Reforming of Methane. *ChemSusChem* **2015**, *8* (21), 3556-3575. DOI: 10.1002/cssc.201500390.
(530) Ke, W.; Stoumpos, C. C.; Kanatzidis, M. G. "Unleaded" Perovskites: Status Quo and Future Prospects of Tin-Based Perovskite Solar Cells. *Adv Mater* **2019**, *31* (47), e1803230. DOI: 10.1002/adma.201803230.
(531) Keen, C. L.; Ensunsa, J. L.; Clegg, M. S. Manganese metabolism in animals and humans including the toxicity of manganese. *Met Ions Biol Syst* **2000**, *37*, 89-121.
(532) Keen, C. L.; Zidenbergcherr, S.; Lonnerdal, B. Dietary Manganese Toxicity and Deficiency - Effects on Cellular Manganese Metabolism. *Acs Symposium Series* **1987**, *354*, 21-34.
(533) Keith, L. S.; Moffett, D. B.; Rosemond, Z. A.; Wohlers, D. W.; Agency for Toxic, S.; Disease, R. ATSDR evaluation of health effects of tungsten and relevance to public health. *Toxicol Ind Health* **2007**, *23* (5-6), 347-387. DOI: 10.1177/0748233707076767.
(534) Keith, L. S.; Wohlers, D. W.; Moffett, D. B.; Rosemond, Z. A.; Agency for Toxic, S.; Disease, R. ATSDR evaluation of potential for human exposure to tungsten. *Toxicol Ind Health* **2007**, *23* (5-6), 309-345. DOI: 10.1177/0748233707081906.
(535) Kempton, H.; Lindberg, R.; Runnells, D. Numerical modeling of platinum Eh measurements by using heterogeneous electron-transfer kinetics. **1990**.
(536) Kennedy, G. L., Jr. Toxicology of fluorine-containing monomers. *Crit Rev Toxicol* **1990**, *21* (2), 149-170. DOI: 10.3109/10408449009089877.
(537) Kenny, R. G.; Marmion, C. J. Toward Multi-Targeted Platinum and Ruthenium Drugs-A New Paradigm in Cancer Drug Treatment Regimens? *Chem Rev* **2019**, *119* (2), 1058-1137. DOI: 10.1021/acs.chemrev.8b00271.
(538) Kesler, S. E.; Gruber, P. W.; Medina, P. A.; Keoleian, G. A.; Everson, M. P.; Wallington, T. J. Global lithium resources: Relative importance of pegmatite, brine and other deposits. *Ore Geology Reviews* **2012**, *48*, 55-69. DOI: 10.1016/j.oregeorev.2012.05.006.
(539) Khairinisa, M. A.; Ariyani, W.; Tsushima, Y.; Koibuchi, N. Effects of Gadolinium Deposits in the Cerebellum: Reviewing the Literature from In Vitro Laboratory Studies to In Vivo Human Investigations. *Int J Environ Res Public Health* **2021**, *18* (14). DOI: 10.3390/ijerph18147214.
(540) Khan, A. M.; Bakar, N. K. A.; Bakar, A. F. A.; Ashraf, M. A. Chemical speciation and bioavailability of rare earth elements (REEs) in the ecosystem: a review. *Environ Sci Pollut Res Int* **2017**, *24* (29), 22764-22789. DOI: 10.1007/s11356-016-7427-1.
(541) Khan, I.; Awan, S. A.; Rizwan, M.; Ali, S.; Zhang, X.; Huang, L. Arsenic behavior in soil-plant system and its detoxification mechanisms in plants: A review. *Environ Pollut* **2021**, *286*, 117389. DOI: 10.1016/j.envpol.2021.117389.
(542) Khan, M. A.; Ho, Y. S. Arsenic in Drinking Water: A Review on Toxicological Effects, Mechanism of Accumulation and Remediation. *Asian Journal of Chemistry* **2011**, *23* (5), 1889-1901.
(543) Khan, N. I.; Owens, G.; Bruce, D.; Naidu, R. Human arsenic exposure and risk assessment at the landscape level: a review. *Environmental Geochemistry and Health* **2009**, *31*, 143-166. DOI: 10.1007/s10653-008-9240-3.
(544) Kidd, P. S.; Bani, A.; Benizri, E.; Gonnelli, C.; Hazotte, C.; Kisser, J.; Konstantinou, M.; Kuppens, T.; Kyrkas, D.; Laubie, B.; Malina, R.; Morel, J. L.; Olcay, H.; Pardo, T.; Pons, M. N.; Prieto-Fernandez, A.; Puschenreiter, M.; Quintela-Sabaris, C.; Ridard, C.; Rodriguez-Garrido,

B.; Rosenkranz, T.; Rozpadek, P.; Saad, R.; Selvi, F.; Simonnot, M. O.; Tognacchini, A.; Turnau, K.; Wazny, R.; Witters, N.; Echevarria, G. Developing Sustainable Agromining Systems in Agricultural Ultramafic Soils for Nickel Recovery. *Frontiers in Environmental Science* **2018**, *6,* 44. DOI: 10.3389/fenvs.2018.00044.
(545) Kielhorn, J.; Melber, C.; Keller, D.; Mangelsdorf, I. Palladium--a review of exposure and effects to human health. *Int J Hyg Environ Health* **2002**, *205* (6), 417-432. DOI: 10.1078/1438-4639-00180.
(546) Kilgore, C. C.; Pelham, L. The Worldwide Availability of Fluorspar. *Natural Resources Forum* **1987**, *11* (2), 127-140. DOI: 10.1111/j.1477-8947.1987.tb00300.x.
(547) Kim, D.-M.; Kim, D.-K.; Lee., S.-H. Manganese coprecipitation/adsorption behaviour and sludge volume ratios in chemical treatment systems for mine drainage: a review of the literature and a pilot-scale experiment. *Water and Environment Journal* **2018**, *32*.
(548) Kim, J. H.; Anwer, H.; Kim, Y. S.; Park, J. W. Decontamination of radioactive cesium-contaminated soil/concrete with washing and washing supernatant- critical review. *Chemosphere* **2021**, *280,* 130419. DOI: 10.1016/j.chemosphere.2021.130419.
(549) Kimbrough, D. E.; Cohen, Y.; Winer, A. M.; Creelman, L.; Mabuni, C. A critical assessment of chromium in the environment. *Critical Reviews in Environmental Science and Technology* **1999**, *29* (1), 1-46. DOI: 10.1080/10643389991259164.
(550) Kirklin, D. R. Properties of materials and systems of importance to environmental fates and remediation. III. Review of previous thermodynamic property values for chromium and some of its compounds. *Journal of Physical and Chemical Reference Data* **1999**, *28* (6), 1675-1704. DOI: 10.1063/1.556053.
(551) Kitao, M.; Lei, T. T.; Nakamura, T.; Koike, T. Manganese toxicity as indicated by visible foliar symptoms of Japanese white birch (Betula platyphylla var. japonica). *Environmental Pollution* **2001**, *111* (1), 89-94. DOI: 10.1016/S0269-7491(99)00332-2.
(552) Kletzin, A.; Adams, M. W. Tungsten in biological systems. *FEMS Microbiol Rev* **1996**, *18* (1), 5-63. DOI: 10.1016/0168-6445(95)00025-9.
(553) Knappe, A.; Moller, P.; Dulski, P.; Pekdeger, A. Positive gadolinium anomaly in surface water and ground water of the urban area Berlin, Germany. *Chemie Der Erde-Geochemistry* **2005**, *65* (2), 167-189. DOI: 10.1016/j.chemer.2004.08.004.
(554) Kochian, L. V. Cellular Mechanisms of Aluminum Toxicity and Resistance in Plants. *Annual Review of Plant Physiology and Plant Molecular Biology* **1995**, *46*, 237-260. DOI: 10.1146/annurev.pp.46.060195.001321.
(555) Kochian, L. V.; Pineros, M. A.; Hoekenga, O. A. The physiology, genetics and molecular biology of plant aluminum resistance and toxicity. *Plant and Soil* **2005**, *274* (1-2), 175-195. DOI: 10.1007/s11104-004-1158-7.
(556) Kochian, L. V.; Pineros, M. A.; Liu, J.; Magalhaes, J. V. Plant Adaptation to Acid Soils: The Molecular Basis for Crop Aluminum Resistance. *Annu Rev Plant Biol* **2015**, *66*, 571-598. DOI: 10.1146/annurev-arplant-043014-114822.
(557) Koh, K. Y.; Yang, Y.; Chen, J. P. Critical review on lanthanum-based materials used for water purification through adsorption of inorganic contaminants. *Critical Reviews in Environmental Science and Technology* **2022**, *52* (10), 1773-1823. DOI: 10.1080/10643389.2020.1864958.
(558) Komarek, M.; Vanek, A.; Ettler, V. Chemical stabilization of metals and arsenic in contaminated soils using oxides--a review. *Environ Pollut* **2013**, *172*, 9-22. DOI: 10.1016/j.envpol.2012.07.045.

(559) Komorowicz, I.; Baralkiewicz, D. Arsenic and its speciation in water samples by high performance liquid chromatography inductively coupled plasma mass spectrometry--last decade review. *Talanta* **2011**, *84* (2), 247-261. DOI: 10.1016/j.talanta.2010.10.065.
(560) Konietzka, R. Gastrointestinal absorption of uranium compounds--a review. *Regul Toxicol Pharmacol* **2015**, *71* (1), 125-133. DOI: 10.1016/j.yrtph.2014.08.012.
(561) Korte, N. E.; Fernando, Q. A Review of Arsenic(Iii) in Groundwater. *Critical Reviews in Environmental Control* **1991**, *21* (1), 1-39. DOI: Doi 10.1080/10643389109388408.
(562) Koshevaya, E.; Krivoshapkina, E.; Krivoshapkin, P. Tantalum oxide nanoparticles as an advanced platform for cancer diagnostics: a review and perspective. *Journal of Materials Chemistry B* **2021**, *9* (25), 5008-5024. DOI: 10.1039/d1tb00570g.
(563) Kosiorek, M.; Wyszkowski, M. Effect of Cobalt on the Environment and Living Organisms - a Review. *Applied Ecology and Environmental Research* **2019**, *17* (5), 11419-11449. DOI: 10.15666/aeer/1705_1141911449.
(564) Kostova, I. Ruthenium complexes as anticancer agents. *Current Medicinal Chemistry* **2006**, *13* (9), 1085-1107. DOI: 10.2174/092986706776360941.
(565) Koutsospyros, A.; Braida, W.; Christodoulatos, C.; Dermatas, D.; Strigul, N. A review of tungsten: from environmental obscurity to scrutiny. *J Hazard Mater* **2006**, *136* (1), 1-19. DOI: 10.1016/j.jhazmat.2005.11.007.
(566) Kravchenko, J.; Darrah, T. H.; Miller, R. K.; Lyerly, H. K.; Vengosh, A. A review of the health impacts of barium from natural and anthropogenic exposure. *Environ Geochem Health* **2014**, *36* (4), 797-814. DOI: 10.1007/s10653-014-9622-7.
(567) Kreiss, K.; Day, G. A.; Schuler, C. R. Beryllium: a modern industrial hazard. *Annu Rev Public Health* **2007**, *28*, 259-277. DOI: 10.1146/annurev.publhealth.28.021406.144011.
(568) Krivovichev, V. G.; Krivovichev, S. V.; Charykova, M. V. Tellurium Minerals: Structural and Chemical Diversity and Complexity. *Minerals* **2020**, *10* (7), 623. DOI: 10.3390/min10070623.
(569) Kruger, M. C.; Bertin, P. N.; Heipieper, H. J.; Arsene-Ploetze, F. Bacterial metabolism of environmental arsenic--mechanisms and biotechnological applications. *Appl Microbiol Biotechnol* **2013**, *97* (9), 3827-3841. DOI: 10.1007/s00253-013-4838-5.
(570) Kszos, L. A.; Stewart, A. J. Review of lithium in the aquatic environment: distribution in the United States, toxicity and case example of groundwater contamination. *Ecotoxicology* **2003**, *12* (5), 439-447. DOI: 10.1023/a:1026112507664.
(571) Kumar, A.; Jigyasu, D. K.; Kumar, A.; Subrahmanyam, G.; Mondal, R.; Shabnam, A. A.; Cabral-Pinto, M. M. S.; Malyan, S. K.; Chaturvedi, A. K.; Gupta, D. K.; Fagodiya, R. K.; Khan, S. A.; Bhatia, A. Nickel in terrestrial biota: Comprehensive review on contamination, toxicity, tolerance and its remediation approaches. *Chemosphere* **2021**, *275*, 129996. DOI: 10.1016/j.chemosphere.2021.129996.
(572) Kumar, M.; Rani, S.; Singh, Y.; Gour, K. S.; Singh, V. N. Tin-selenide as a futuristic material: properties and applications. *Rsc Advances* **2021**, *11* (12), 6477-6503. DOI: 10.1039/d0ra09807h.
(573) Kumaresan, D.; Shankar, K.; Vaidya, S.; Schmehl, R. H. Photochemistry and photophysics of coordination compounds: Osmium. *Photochemistry and Photophysics of Coordination Compounds II* **2007**, *281*, 101-142. DOI: 10.1007/128_2007_140.
(574) Kumari, A.; Jha, M. K.; Pathak, D. D.; Chakravarty, S.; Lee, J. C. Processes developed for the separation of europium (Eu) from various resources. *Separation and Purification Reviews* **2019**, *48* (2), 91-121. DOI: 10.1080/15422119.2018.1454959.

(575) Kumari, B.; Kumar, V.; Sinha, A. K.; Ahsan, J.; Ghosh, A. K.; Wang, H. P.; DeBoeck, G. Toxicology of arsenic in fish and aquatic systems. *Environmental Chemistry Letters* **2017**, *15* (1), 43-64. DOI: 10.1007/s10311-016-0588-9.
(576) Kustin, K. Aqueous vanadium ion dynamics relevant to bioinorganic chemistry: A review. *J Inorg Biochem* **2015**, *147*, 32-38. DOI: 10.1016/j.jinorgbio.2014.12.009.
(577) Kuzmenko; V., M. The geochemistry of tantalum and niobium. *International Geology Review* **1961**, *3*.
(578) Kwon, Y.; Huang, P.; Patel, M. V.; Blume, D.; Whaley, K. B. Quantum solvation and molecular rotations in superfluid helium clusters. *Journal of Chemical Physics* **2000**, *113* (16), 6469-6501. DOI: 10.1063/1.1310608.
(579) Lacson, C. F. Z.; Lu, M. C.; Huang, Y. H. Fluoride network and circular economy as potential model for sustainable development-A review. *Chemosphere* **2020**, *239,* 124662. DOI: 10.1016/j.chemosphere.2019.124662.
(580) Lagarde, F.; Leroy, M. Metabolism and toxicity of tungsten in humans and animals. *Met Ions Biol Syst* **2002**, *39*, 741-759..
(581) Landa, E. R. Uranium mill tailings: nuclear waste and natural laboratory for geochemical and radioecological investigations. *J Environ Radioact* **2004**, *77* (1), 1-27. DOI: 10.1016/j.jenvrad.2004.01.030.
(582) Landais, P. Organic geochemistry of sedimentary uranium ore deposits. *Ore Geology Reviews* **1996**, *11* (1-3), 33-51. DOI: 10.1016/0169-1368(95)00014-3.
(583) Langdon, C. J.; Piearce, T. G.; Meharg, A. A.; Semple, K. T. Interactions between earthworms and arsenic in the soil environment: a review. *Environmental Pollution* **2003**, *124* (3), 361-373. DOI: 10.1016/S0269-7491(03)00047-2.
(584) Lange, B.; van der Ent, A.; Baker, A. J.; Echevarria, G.; Mahy, G.; Malaisse, F.; Meerts, P.; Pourret, O.; Verbruggen, N.; Faucon, M. P. Copper and cobalt accumulation in plants: a critical assessment of the current state of knowledge. *New Phytol* **2017**, *213* (2), 537-551. DOI: 10.1111/nph.14175.
(585) Langmuir, D. Uranium: Mineralogy, Geochemistry, and the Environment. Peter C. Burns and Robert Finch, Editors, Reviews in Mineralogy, 1999, Vol. 38, 679 p. Mineralogical Society of America, Washington, DC 32(24 for MSA members). **2001**.
(586) LaZerte, B.; VanLoon, G; Anderson, B. Aluminum in water. *Research issues in aluminum toxicity* **1997**.
(587) Le Fur, M.; Caravan, P. The biological fate of gadolinium-based MRI contrast agents: a call to action for bioinorganic chemists. *Metallomics* **2019**, *11* (2), 240-254. DOI: 10.1039/c8mt00302e.
(588) Lee, S. A review on types of vanadium deposits and process mineralogical characteristics. *Journal of the Korean Society of Mineral and Energy Resource Engineers* **2020**, 57.
(589) Lee, H.; Trevors, J. T.; Van Dyke, M. I. Microbial interactions with germanium. *Biotechnol Adv* **1990**, *8* (3), 539-546. DOI: 10.1016/0734-9750(90)90647-t.
(590) Lee, J. C.; Kurniawan; Hong, H. J.; Chung, K. W.; Kim, S. Separation of platinum, palladium and rhodium from aqueous solutions using ion exchange resin: A review. *Separation and Purification Technology* **2020**, *246,* 116896. DOI: 10.1016/j.seppur.2020.116896.
(591) Lee, K. M.; Lai, C. W.; Ngai, K. S.; Juan, J. C. Recent developments of zinc oxide based photocatalyst in water treatment technology: A review. *Water Res* **2016**, *88*, 428-448. DOI: 10.1016/j.watres.2015.09.045.

(592) Lee, P. H. Indium and gallium-mediated addition reactions. *Bulletin of the Korean Chemical Society* **2007**, *28* (1), 17-28.
(593) Lemus, R.; Venezia, C. F. An update to the toxicological profile for water-soluble and sparingly soluble tungsten substances. *Crit Rev Toxicol* **2015**, *45* (5), 388-411. DOI: 10.3109/10408444.2014.1003422.
(594) Leonhard, M. J.; Chang, E. T.; Loccisano, A. E.; Garry, M. R. A systematic literature review of epidemiologic studies of developmental manganese exposure and neurodevelopmental outcomes. *Toxicology* **2019**, *420*, 46-65. DOI: 10.1016/j.tox.2019.03.004.
(595) Leso, V.; Iavicoli, I. Palladium Nanoparticles: Toxicological Effects and Potential Implications for Occupational Risk Assessment. *Int J Mol Sci* **2018**, *19* (2). DOI: 10.3390/ijms19020503.
(596) Lestaevel, P.; Racine, R.; Bensoussan, H.; Rouas, C.; Gueguen, Y.; Dublineau, I.; Bertho, J. M.; Gourmelon, P.; Jourdain, J. R.; Souidi, M. Caesium 137: Properties and biological effects resulting of an internal contamination. *Medecine Nucleaire-Imagerie Fonctionnelle Et Metabolique* **2010**, *34* (2), 108-118. DOI: 10.1016/j.mednuc.2009.12.003.
(597) Levina; Aviva. Chromium in biology: toxicology and nutritional aspects. *Progress in inorganic chemistry* **2002**, *51*.
(598) Levina, A.; Lay, P. A. Mechanistic studies of relevance to the biological activities of chromium. *Coordination Chemistry Reviews* **2005**, *249* (3-4), 281-298. DOI: 10.1016/j.ccr.2004.02.017.
(599) Lewis, B. L.; Andreae, M. O.; Froelich, P. N.; Mortlock, R. A. A Review of the Biogeochemistry of Germanium in Natural-Waters. *Science of the Total Environment* **1988**, *73* (1-2), 107-120. DOI: 10.1016/0048-9697(88)90191-X.
(600) Leyssens, L.; Vinck, B.; Van Der Straeten, C.; Wuyts, F.; Maes, L. Cobalt toxicity in humans-A review of the potential sources and systemic health effects. *Toxicology* **2017**, *387*, 43-56. DOI: 10.1016/j.tox.2017.05.015.
(601) Li, C. J.; Wang, J. H.; Yan, B.; Miao, A. J.; Zhong, H.; Zhang, W.; Ma, L. Q. Progresses and emerging trends of arsenic research in the past 120 years. *Critical Reviews in Environmental Science and Technology* **2021**, *51* (13), 1306-1353. DOI: 10.1080/10643389.2020.1752611.
(602) Li, F. F.; Collins, J. G.; Keene, F. R. Ruthenium complexes as antimicrobial agents. *Chemical Society Reviews* **2015**, *44* (8), 2529-2542. DOI: 10.1039/c4cs00343h.
(603) Li, H.; Fan, J.; Peng, X. Colourimetric and fluorescent probes for the optical detection of palladium ions. *Chem Soc Rev* **2013**, *42* (19), 7943-7962. DOI: 10.1039/c3cs60123d.
(604) Li, H. M.; Dong, H.; Li, J. R.; Wu, Z. X. Recent Advances in Tin-Based Perovskite Solar Cells. *Acta Physico-Chimica Sinica* **2021**, *37* (4), 2007006. DOI: 10.3866/Pku.Whxb202007006.
(605) Li, J.; Cen, Y. Y.; Li, Y. The research advances in the mechanism of manganese-induced neurotoxicity. *Toxin Reviews* **2019**, *38* (1), 54-60. DOI: 10.1080/15569543.2018.1486859.
(606) Li, J.; Jia, Y.; Dong, R.; Huang, R.; Liu, P.; Li, X.; Wang, Z.; Liu, G.; Chen, Z. Advances in the Mechanisms of Plant Tolerance to Manganese Toxicity. *Int J Mol Sci* **2019**, *20* (20). DOI: 10.3390/ijms20205096.
(607) Li, J.; Li, H.; Zhan, G.; Zhang, L. Solar Water Splitting and Nitrogen Fixation with Layered Bismuth Oxyhalides. *Acc Chem Res* **2017**, *50* (1), 112-121. DOI: 10.1021/acs.accounts.6b00523.
(608) Li, J.; Pang, S. Y.; Wang, Z.; Guo, Q.; Duan, J. B.; Sun, S. F.; Wang, L. H.; Cao, Y.; Jiang, J. Oxidative transformation of emerging organic contaminants by aqueous permanganate:

Kinetics, products, toxicity changes, and effects of manganese products. *Water Research* **2021**, *203,* 117513. DOI: 10.1016/j.watres.2021.117513.
(609) Li, J.; Wang, Q.; Oremland, R. S.; Kulp, T. R.; Rensing, C.; Wang, G. Microbial Antimony Biogeochemistry: Enzymes, Regulation, and Related Metabolic Pathways. *Appl Environ Microbiol* **2016**, *82* (18), 5482-5495. DOI: 10.1128/AEM.01375-16.
(610) Li, J.; Zheng, B.; He, Y.; Zhou, Y.; Chen, X.; Ruan, S.; Yang, Y.; Dai, C.; Tang, L. Antimony contamination, consequences and removal techniques: A review. *Ecotoxicol Environ Saf* **2018**, *156*, 125-134. DOI: 10.1016/j.ecoenv.2018.03.024.
(611) Li, M.; Yin, J. J.; Wamer, W. G.; Lo, Y. M. Mechanistic characterization of titanium dioxide nanoparticle-induced toxicity using electron spin resonance. *J Food Drug Anal* **2014**, *22* (1), 76-85. DOI: 10.1016/j.jfda.2014.01.006.
(612) Li, Y.; Lu, Y.; Adelhelm, P.; Titirici, M. M.; Hu, Y. S. Intercalation chemistry of graphite: alkali metal ions and beyond. *Chem Soc Rev* **2019**, *48* (17), 4655-4687. DOI: 10.1039/c9cs00162j.
(613) Li, Y. C.; Xu, Z.; Ma, H. Q.; Hursthouse, A. S. Removal of Manganese(II) from Acid Mine Wastewater: A Review of the Challenges and Opportunities with Special Emphasis on Mn-Oxidizing Bacteria and Microalgae. *Water* **2019**, *11* (12), 2493. DOI: 10.3390/w11122493.
(614) Liang, T.; Ding, S. M.; Song, W. C.; Chong, Z. Y.; Zhang, C. S.; Li, H. T. A review of fractionations of rare earth elements in plants. *Journal of Rare Earths* **2008**, *26* (1), 7-15. DOI: 10.1016/S1002-0721(08)60027-7.
(615) Liao, C.; Jin, Y.; Li, Y.; Tjong, S. C. Interactions of Zinc Oxide Nanostructures with Mammalian Cells: Cytotoxicity and Photocatalytic Toxicity. *Int J Mol Sci* **2020**, *21* (17). DOI: 10.3390/ijms21176305.
(616) Lima, A. T.; Ottosen, L. Recovering rare earth elements from contaminated soils: Critical overview of current remediation technologies. *Chemosphere* **2021**, *265*, 129163. DOI: 10.1016/j.chemosphere.2020.129163.
(617) Lin, Y.; Genzer, J.; Dickey, M. D. Attributes, Fabrication, and Applications of Gallium-Based Liquid Metal Particles. *Adv Sci (Weinh)* **2020**, *7* (12), 2000192. DOI: 10.1002/advs.202000192.
(618) Linde, S. J. L.; Franken, A.; du Plessis, J. L. Occupational Respiratory Exposure to Platinum Group Metals: A Review and Recommendations. *Chem Res Toxicol* **2017**, *30* (10), 1778-1790. DOI: 10.1021/acs.chemrestox.7b00184.
(619) Lison, D. Human toxicity of cobalt-containing dust and experimental studies on the mechanism of interstitial lung disease (hard metal disease). *Crit Rev Toxicol* **1996**, *26* (6), 585-616. DOI: 10.3109/10408449609037478.
(620) Lison, D.; van den Brule, S.; Van Maele-Fabry, G. Cobalt and its compounds: update on genotoxic and carcinogenic activities. *Crit Rev Toxicol* **2018**, *48* (7), 522-539. DOI: 10.1080/10408444.2018.1491023.
(621) Liu, C.; Qi, Y.; Liu, Y. J. Recent Development of Samarium Diiodide and Other Samarium Reagents in Organic Transformation. *Chinese Journal of Organic Chemistry* **2021**, *41* (6), 2202-2216. DOI: 10.6023/cjoc202011034.
(622) Liu, C. W.; Lin, J.; Cao, H. B.; Zhang, Y.; Sun, Z. Recycling of spent lithium-ion batteries in view of lithium recovery: A critical review. *J Clean Prod* **2019**, *228*, 801-813. DOI: 10.1016/j.jclepro.2019.04.304.
(623) Liu, J.; Pineros, M. A.; Kochian, L. V. The role of aluminum sensing and signaling in plant aluminum resistance. *J Integr Plant Biol* **2014**, *56* (3), 221-230. DOI: 10.1111/jipb.12162.

(624) Liu, K.; Lin, X.; Zhao, J. Toxic effects of the interaction of titanium dioxide nanoparticles with chemicals or physical factors. *Int J Nanomedicine* **2013**, *8*, 2509-2520. DOI: 10.2147/IJN.S46919.
(625) Liu, Q. Q.; Lu, X. F.; Peng, H. Y.; Popowich, A.; Tao, J.; Uppal, J. S.; Yan, X. W.; Boe, D.; Le, X. C. Speciation of arsenic - A review of phenylarsenicals and related arsenic metabolites. *Trac-Trends in Analytical Chemistry* **2018**, *104*, 171-182. DOI: 10.1016/j.trac.2017.10.006.
(626) Liu, W. J.; Huang, L.; Liang, X.; Liu, L. J.; Sun, C. L.; Lin, X. Y. Heat shock induces cross adaptation to aluminum stress through enhancing ascorbate-glutathione cycle in wheat seedlings. *Chemosphere* **2021**, *278,* 130397. DOI: 10.1016/j.chemosphere.2021.130397.
(627) Liu, Y.; Shen, C.; Zhang, X.; Yu, H.; Wang, F.; Wang, Y.; Zhang, L. W. Exposure and nephrotoxicity concern of bismuth with the occurrence of autophagy. *Toxicol Ind Health* **2018**, *34* (3), 188-199. DOI: 10.1177/0748233717746810.
(628) Liu, Y.; Yang, B.; He, H.; Yang, S.; Duan, X.; Wang, S. Bismuth-based complex oxides for photocatalytic applications in environmental remediation and water splitting: A review. *Sci Total Environ* **2022**, *804*, 150215. DOI: 10.1016/j.scitotenv.2021.150215.
(629) Liu, Y. J.; Zhang, Y. M. New progress in the application of samarium reagent to organic synthesis. *Acta Chimica Sinica* **2005**, *63* (5), 341-351.
(630) Liying, H.; Yumin, S.; Lanhong, J.; Shikao, S. Recent advances of cerium oxide nanoparticles in synthesis, luminescence and biomedical studies: a review. *Journal of rare earths* **2015**, *33*.
(631) Lizama, A. K.; Fletcher, T. D.; Sun, G. Z. Removal processes for arsenic in constructed wetlands. *Chemosphere* **2011**, *84* (8), 1032-1043. DOI: 10.1016/j.chemosphere.2011.04.022.
(632) Long, X.; Wang, X.; Guo, X.; He, M. A review of removal technology for antimony in aqueous solution. *J Environ Sci (China)* **2020**, *90*, 189-204. DOI: 10.1016/j.jes.2019.12.008.
(633) Lorand, J. P.; Luguet, A.; Alard, O. Platinum-group element systematics and petrogenetic processing of the continental upper mantle: A review. *Lithos* **2013**, *164*, 2-21. DOI: 10.1016/j.lithos.2012.08.017.
(634) Lord, M. S.; Berret, J. F.; Singh, S.; Vinu, A.; Karakoti, A. S. Redox Active Cerium Oxide Nanoparticles: Current Status and Burning Issues. *Small* **2021**, *17* (51), e2102342. DOI: 10.1002/smll.202102342.
(635) Losi, M. E.; Amrhein, C.; Frankenberger, W. T., Jr. Environmental biochemistry of chromium. *Rev Environ Contam Toxicol* **1994**, *136*, 91-121. DOI: 10.1007/978-1-4612-2656-7_3.
(636) Lou, S. F.; Cheng, X. Q.; Ma, Y. L.; Du, C. Y.; Gao, Y. Z.; Yin, G. P. Nb-Based Oxides as Anode Materials for Lithium Ion Batteries. *Progress in Chemistry* **2015**, *27* (2-3), 297-309. DOI: 10.7536/Pc140932.
(637) Lu, F. H.; Xiao, T. F.; Lin, J.; Ning, Z. P.; Long, Q.; Xiao, L. H.; Huang, F.; Wang, W. K.; Xiao, Q. X.; Lan, X. L.; Chen, H.Y. Resources and extraction of gallium: A review. *Hydrometallurgy* **2017**, *174*, 105-115. DOI: 10.1016/j.hydromet.2017.10.010.
(638) Lu, G.; Haes, A. J.; Forbes, T. Z. Detection and identification of solids, surfaces, and solutions of uranium using vibrational spectroscopy. *Coord Chem Rev* **2018**, *374*, 314-344. DOI: 10.1016/j.ccr.2018.07.010.
(639) Lu, J. M.; Dreisinger, D.; Gluck, T. Manganese electrodeposition - A literature review. *Hydrometallurgy* **2014**, *141*, 105-116. DOI: 10.1016/j.hydromet.2013.11.002.

(640) Lu, Y. J.; Zhu, L. Y.; Agrafiotis, C.; Vieten, J.; Roeb, M.; Sattler, C. Solar fuels production: Two-step thermochemical cycles with cerium-based oxides. *Progress in Energy and Combustion Science* **2019**, *75,* 100785. DOI: 10.1016/j.pecs.2019.100785.
(641) Luo, Z.; Li, Z.; Xie, Z.; Sokolova, I.; Peijnenburg, W.; Hu, M.; Wang, Y. Rethinking nano-TiO2 safety: overview of toxic effects in humans and aquatic animals. *Small* **2020**, 16.
(642) Luong, V. T.; Canas Kurz, E. E.; Hellriegel, U.; Luu, T. L.; Hoinkis, J.; Bundschuh, J. Iron-based subsurface arsenic removal technologies by aeration: A review of the current state and future prospects. *Water Res* **2018**, *133*, 110-122. DOI: 10.1016/j.watres.2018.01.007.
(643) Luvonga, C.; Rimmer, C. A.; Yu, L. L.; Lee, S. B. Organoarsenicals in Seafood: Occurrence, Dietary Exposure, Toxicity, and Risk Assessment Considerations - A Review. *Journal of Agricultural and Food Chemistry* **2020**, *68* (4), 943-960. DOI: 10.1021/acs.jafc.9b07532.
(644) Lyu, F.; Hu, Y.; Wang, L.; Sun, W. Dealkalization processes of bauxite residue: A comprehensive review. *J Hazard Mater* **2021**, *403*, 123671. DOI: 10.1016/j.jhazmat.2020.123671.
(645) Ma, J.; Sengupta, M. K.; Yuan, D.; Dasgupta, P. K. Speciation and detection of arsenic in aqueous samples: a review of recent progress in non-atomic spectrometric methods. *Anal Chim Acta* **2014**, *831*, 1-23. DOI: 10.1016/j.aca.2014.04.029.
(646) Ma, J. F. Syndrome of aluminum toxicity and diversity of aluminum resistance in higher plants. *Int Rev Cytol* **2007**, *264*, 225-252. DOI: 10.1016/S0074-7696(07)64005-4.
(647) Ma, J. Y.; Guo, X. T.; Xue, H. G.; Pan, K. M.; Liu, C. S.; Pang, H. Niobium/tantalum-based materials: Synthesis and applications in electrochemical energy storage. *Chemical Engineering Journal* **2020**, *380,* 122428. DOI: 10.1016/j.cej.2019.122428.
(648) Ma, M.; Wang, R.; Xu, L.; Xu, M.; Liu, S. Emerging health risks and underlying toxicological mechanisms of uranium contamination: Lessons from the past two decades. *Environ Int* **2020**, *145*, 106107. DOI: 10.1016/j.envint.2020.106107.
(649) Ma, Z. L.; Lin, L. D.; Wu, M. J.; Yu, H. G.; Shang, T. G.; Zhang, T. T.; Zhao, M. Total and inorganic arsenic contents in seaweeds: Absorption, accumulation, transformation and toxicity. *Aquaculture* **2018**, *497*, 49-55. DOI: 10.1016/j.aquaculture.2018.07.040.
(650) Maachou, A.; Amrani, B.; Driz, M. Structural and electronic properties of III-V scandium compounds. *Physica B-Condensed Matter* **2007**, *388* (1-2), 384-389. DOI: 10.1016/j.physb.2006.06.145.
(651) Mackay, D. A. R.; Simandl, G. J. Geology, market and supply chain of niobium and tantalum-a review. *Mineralium Deposita* **2014**, *49* (8), 1025-1047. DOI: 10.1007/s00126-014-0551-2.
(652) Macomber, L.; Hausinger, R. P. Mechanisms of nickel toxicity in microorganisms. *Metallomics* **2011**, *3* (11), 1153-1162. DOI: 10.1039/c1mt00063b.
(653) Magalhaes, J. V.; Pineros, M. A.; Maciel, L. S.; Kochian, L. V. Emerging Pleiotropic Mechanisms Underlying Aluminum Resistance and Phosphorus Acquisition on Acidic Soils. *Front Plant Sci* **2018**, *9*, 1420. DOI: 10.3389/fpls.2018.01420.
(654) Magotra; Rajni. A new classification scheme of fluorite deposits. *International Journal of Geosciences* **2017**, *8*.
(655) Mahey, S.; Kumar, R.; Sharma, M.; Kumar, V.; Bhardwaj, R. A critical review on toxicity of cobalt and its bioremediation strategies. *Sn Applied Sciences* **2020**, *2* (7), 1279. DOI: 10.1007/s42452-020-3020-9.

(656) Mahmoudi, A.; Shakibania, S.; Mokmeli, M.; Rashchi, F. Tellurium, from Copper Anode Slime to High Purity Product: A Review Paper. *Metallurgical and Materials Transactions B-Process Metallurgy and Materials Processing Science* **2020**, *51* (6), 2555-2575. DOI: 10.1007/s11663-020-01974-x.
(657) Maia, L. C.; Soares, L. C.; Alves Gurgel, L. V. A review on the use of lignocellulosic materials for arsenic adsorption. *J Environ Manage* **2021**, *288*, 112397. DOI: 10.1016/j.jenvman.2021.112397.
(658) Makuei, F. M.; Senanayake, G. Extraction of tellurium from lead and copper bearing feed materials and interim metallurgical products - A short review. *Minerals Engineering* **2018**, *115*, 79-87. DOI: 10.1016/j.mineng.2017.10.013.
(659) Malaviya, P.; Singh, A. Physicochemical Technologies for Remediation of Chromium-Containing Waters and Wastewaters. *Critical Reviews in Environmental Science and Technology* **2011**, *41* (12), 1111-1172. DOI: 10.1080/10643380903392817.
(660) Malvandi, A.; Shahba, S.; Mohammadipour, A.; Rastegar-Moghaddam, S.; Abudayyak, M. Cell and molecular toxicity of lanthanum nanoparticles: are there possible risks to humans? *Nanotoxicology* **2021**, *15*, 951–972. DOI: 10.1080/17435390.2021.1940340
(661) Mandal, B. K.; Suzuki, K. T. Arsenic round the world: a review. *Talanta* **2002**, *58* (1), 201-235.
(662) Manning, D. A. C. Mineral sources of potassium for plant nutrition. A review. *Agronomy for Sustainable Development* **2010**, *30* (2), 281-294. DOI: 10.1051/agro/2009023.
(663) Maqbool, Z.; Asghar, H. N.; Shahzad, T.; Hussain, S.; Riaz, M.; Ali, S.; Arif, M. S.; Maqsood, M. Isolating, screening and applying chromium reducing bacteria to promote growth and yield of okra (Hibiscus esculentus L.) in chromium contaminated soils. *Ecotoxicol Environ Saf* **2015**, *114*, 343-349. DOI: 10.1016/j.ecoenv.2014.07.007.
(664) Markich, S. J. Uranium speciation and bioavailability in aquatic systems: an overview. *ScientificWorldJournal* **2002**, *2*, 707-729. DOI: 10.1100/tsw.2002.130.
(665) Markowska-Szczupak, A.; Endo-Kimura, M.; Paszkiewicz, O.; Kowalska, E. Are Titania Photocatalysts and Titanium Implants Safe? Review on the Toxicity of Titanium Compounds. *Nanomaterials (Basel)* **2020**, *10* (10). DOI: 10.3390/nano10102065.
(666) Martin, K.; Huggins, T.; King, C.; Carroll, M. A.; Catapane, E. J. The neurotoxic effects of manganese on the dopaminergic innervation of the gill of the bivalve mollusc, Crassostrea virginica. *Comparative Biochemistry and Physiology C-Toxicology & Pharmacology* **2008**, *148* (2), 152-159. DOI: 10.1016/j.cbpc.2008.05.004.
(667) Martins, A. C.; Krum, B. N.; Queiros, L.; Tinkov, A. A.; Skalny, A. V.; Bowman, A. B.; Aschner, M. Manganese in the Diet: Bioaccessibility, Adequate Intake, and Neurotoxicological Effects. *J Agric Food Chem* **2020**, *68* (46), 12893-12903. DOI: 10.1021/acs.jafc.0c00641.
(668) Mäser, P,; Gierth, M.; Schroeder, J. Molecular mechanisms of potassium and sodium uptake in plants, in Horst, W. et al. *Progress in Plant Nutrition: Plenary Lectures of the XIV International Plant Nutrition Colloquium. Developments in Plant and Soil Sciences 98*, Springer, Dordrecht **2002**. DOI: 10.1007/978-94-017-2789-1_3
(669) Masuda, H. Arsenic cycling in the Earth's crust and hydrosphere: interaction between naturally occurring arsenic and human activities. *Progress in Earth and Planetary Science* **2018**, *5,* 68. DOI: 10.1186/s40645-018-0224-3.
(670) Matschullat, J. Arsenic in the geosphere--a review. *Sci Total Environ* **2000**, *249* (1-3), 297-312. DOI: 10.1016/s0048-9697(99)00524-0.

(671) Matsumoto, H. Cell biology of aluminum toxicity and tolerance in higher plants. *Int Rev Cytol* **2000**, *200*, 1-46. DOI: 10.1016/s0074-7696(00)00001-2.
(672) Matsumoto, H.; Motoda, H. Aluminum toxicity recovery processes in root apices. Possible association with oxidative stress. *Plant Sci* **2012**, *185-186*, 1-8. DOI: 10.1016/j.plantsci.2011.07.019.
(673) Matsumura, Y.; Sugiyama, S.; Moffat, J. Oxidative coupling of methane over praseodymium oxide in the presence and absence of tetrachloromethane. In Oyama, S.; Hightower, J. eds. *Catalytic Selective Oxidation, ACS Symposium Series 523*, **1993**. DOI: 10.1021/bk-1993-0523.ch025
(674) Mawia, A. M.; Hui, S.; Zhou, L.; Li, H.; Tabassum, J.; Lai, C.; Wang, J.; Shao, G.; Wei, X.; Tang, S.; Luo, J.; Hu, S.; Hu, P. Inorganic arsenic toxicity and alleviation strategies in rice. *J Hazard Mater* **2021**, *408*, 124751. DOI: 10.1016/j.jhazmat.2020.124751.
(675) Mays, D. E.; Hussam, A. Voltammetric methods for determination and speciation of inorganic arsenic in the environment--a review. *Anal Chim Acta* **2009**, *646* (1-2), 6-16. DOI: 10.1016/j.aca.2009.05.006.
(676) McMahon, J.; Snodgrass, W. Chemical equilibrium data bases and tools for calculating zinc sulfide geochemistry in freshwater sediments and its toxicity. *Water Quality Research Journal* **1996**, *31*, 577-592. DOI: 10.2166/wqrj.1996.032
(677) McNeill, L.; McLean, J.; Edwards, M.; Parks, J. State of the science of hexavalent chromium in drinking water. *Water Research Foundation* **2012**, *6666*, 1-35.
(678) McPhail, C.; Summerhayes, E.; Welch, S.; Brugger, J. The geochemistry and mobility of zinc in the regolith. In Roach I. ed. *Advances in Regolith CRC LEME* **2003**, 287-291.
(679) McVey, B. F. P.; Prabakar, S.; Gooding, J. J.; Tilley, R. D. Solution Synthesis, Surface Passivation, Optical Properties, Biomedical Applications, and Cytotoxicity of Silicon and Germanium Nanocrystals. *Chempluschem* **2017**, *82* (1), 60-73. DOI: 10.1002/cplu.201600207.
(680) Mederos, A.; Dominguez, S.; Chinea, E.; Brito, F.; Cecconi, F. Review: new advances in the coordination chemistry of the beryllium(II). *Journal of Coordination Chemistry* **2001**, *53* (3), 191-222. DOI: 10.1080/00958970108022906.
(681) Meehan, P. R.; Aris, D. R.; Willey, G. R. Structural chemistry of Sc(III): an overview. *Coordination Chemistry Reviews* **1999**, *181*, 121-145. DOI: 10.1016/S0010-8545(98)00214-8.
(682) Meharg, A. A.; Hartley-Whitaker, J. Arsenic uptake and metabolism in arsenic resistant and nonresistant plant species. *New Phytologist* **2002**, *154* (1), 29-43. DOI: 10.1046/j.1469-8137.2002.00363.x.
(683) Meharg, A. A.; Meharg, C. The Pedosphere as a Sink, Source, and Record of Anthropogenic and Natural Arsenic Atmospheric Deposition. *Environmental Science & Technology* **2021**, *55* (12), 7757-7769. DOI: 10.1021/acs.est.1c00460.
(684) Mehdi, S. E. H.; Amen, R.; Ali, A.; Anjum, H.; Mahmood, A.; Mubashir, M.; Mukhtar, A.; Ullah, S.; Al-Sehemi, A. G.; Ibrahim, M.; Khan, M.S.; Qyyum, M.A.; Show, P.L. Sources, chemistry, bioremediation and social aspects of arsenic-contaminated waters: a review. *Environmental Chemistry Letters* **2021**, *19* (5), 3859-3886. DOI: 10.1007/s10311-021-01254-3.
(685) Meisch, H. U.; Bielig, H. J. Chemistry and biochemistry of vanadium. *Basic Res Cardiol* **1980**, *75* (3), 413-417. DOI: 10.1007/BF01908403.
(686) Melson, G.; Stotz, R. The coordination chemistry of scandium. *Coordination Chemistry Reviews* **1971**, *7*, 133-160. DOI: 10.1016/S0010-8545(00)80215-5

(687) Meng, F.; McNeice, J.; Zadeh, S.; Ghahreman, A. Review of lithium production and recovery from minerals, brines, and lithium-ion batteries. *Mineral Processing and Extractive Metallurgy Review* **2019**, *42*, 123-141. DOI: 10.1080/08827508.2019.1668387
(688) Meng, Y. M.; Hu, R. Z.; Huang, X. W.; Gao, J. F. Germanium in Magnetite: A Preliminary Review. *Acta Geologica Sinica-English Edition* **2017**, *91* (2), 711-726. DOI: 10.1111/1755-6724.13127.
(689) Meshram; Pratima; Abhilash. Recovery and recycling of cerium from primary and secondary resources-a critical review. *Mineral Processing and Extractive Metallurgy Review* **2020**, *41*.
(690) Meshram, P.; Pandey, B. Advanced review on extraction of nickel from primary and secondary sources. *Mineral Processing and Extractive Metallurgy Review* **2018**, *40*, 157-193. DOI: 10.1080/08827508.2018.1514300
(691) Meyer, J. S.; Lyons-Darden, T.; Garman, E. R.; Middleton, E. T.; Schlekat, C. E. Toxicity of Nanoparticulate Nickel to Aquatic Organisms: Review and Recommendations for Improvement of Toxicity Tests. *Environ Toxicol Chem* **2020**, *39* (10), 1861-1883. DOI: 10.1002/etc.4812.
(692) Miao, W.; Zhu, B.; Xiao, X.; Li, Y.; Dirbaba, N. B.; Zhou, B.; Wu, H. Effects of titanium dioxide nanoparticles on lead bioconcentration and toxicity on thyroid endocrine system and neuronal development in zebrafish larvae. *Aquat Toxicol* **2015**, *161*, 117-126. DOI: 10.1016/j.aquatox.2015.02.002.
(693) Michalke, B.; Fernsebner, K. New insights into manganese toxicity and speciation. *Journal of Trace Elements in Medicine and Biology* **2014**, *28* (2), 106-116. DOI: 10.1016/j.jtemb.2013.08.005.
(694) Michalke, B.; Halbach, S.; Nischwitz, V. Speciation and toxicological relevance of manganese in humans. *J Environ Monit* **2007**, *9* (7), 650-656. DOI: 10.1039/b704173j.
(695) Middlesworth, P.; Wood, S. The aqueous geochemistry of the rare earth elements and yttrium. Part 7. REE, Th and U contents in thermal springs associated with the Idaho batholith. *Applied Geochemistry* **1998**, *13*, 861-884. DOI: 10.1016/S0883-2927(98)00019-5
(696) Migaszewski, Z. M.; Galuszka, A. The Characteristics, Occurrence, and Geochemical Behavior of Rare Earth Elements in the Environment: A Review. *Critical Reviews in Environmental Science and Technology* **2015**, *45* (5), 429-471. DOI: 10.1080/10643389.2013.866622.
(697) Migdisov, A.; Williams-Jones, A. E.; Brugger, J.; Caporuscio, F. A. Hydrothermal transport, deposition, and fractionation of the REE: Experimental data and thermodynamic calculations. *Chemical Geology* **2016**, *439*, 13-42. DOI: 10.1016/j.chemgeo.2016.06.005.
(698) Mihaylov, I.; Distin, P. A. Gallium Solvent-Extraction in Hydrometallurgy - an Overview. *Hydrometallurgy* **1992**, *28* (1), 13-27. DOI: 10.1016/0304-386x(92)90062-5.
(699) Mihucz, V. G.; Zaray, G. Occurrence of antimony and phthalate esters in polyethylene terephthalate bottled drinking water. *Applied Spectroscopy Reviews* **2016**, *51* (3), 163-189. DOI: 10.1080/05704928.2015.1105243.
(700) Milinovic, J.; Rodrigues, F. J. L.; Barriga, F. J. A. S.; Murton, B. J. Ocean-Floor Sediments as a Resource of Rare Earth Elements: An Overview of Recently Studied Sites. *Minerals* **2021**, *11* (2), 142. DOI: 10.3390/min11020142.
(701) Mioduski, T.; Guminski, C.; Zeng, D. W. IUPAC-NIST Solubility Data Series. 87. Rare Earth Metal Chlorides in Water and Aqueous Systems. Part 1. Scandium Group (Sc, Y, La).

Journal of Physical and Chemical Reference Data **2008**, *37* (4), 1765-1853. DOI: 10.1063/1.2956740.
(702) Miretzky, P.; Cirelli, A. F. Remediation of Arsenic-Contaminated Soils by Iron Amendments: A Review. *Critical Reviews in Environmental Science and Technology* **2010**, *40* (2), 93-115. DOI: 10.1080/10643380802202059.
(703) Mishra, R. K.; Tiwari, S.; Patel, A.; Prasad, S. M. Arsenic contamination, speciation, toxicity and defense strategies in plants. *Brazilian Journal of Botany* **2021**, *44* (1), 1-10. DOI: 10.1007/s40415-020-00694-5.
(704) Missen, O. P.; Ram, R.; Mills, S. J.; Etschmann, B.; Reith, F.; Shuster, J.; Smith, D. J.; Brugger, J. Love is in the Earth: A review of tellurium (bio)geochemistry in surface environments. *Earth-Science Reviews* **2020**, *204*, 103150. DOI: 10.1016/j.earscirev.2020.103150.
(705) Mitchell, N.; Perez-Sanchez, D.; Thorne, M. C. A review of the behaviour of U-238 series radionuclides in soils and plants. *J Radiol Prot* **2013**, *33* (2), R17-48. DOI: 10.1088/0952-4746/33/2/R17.
(706) Mitchell, R. H. Primary and secondary niobium mineral deposits associated with carbonatites. *Ore Geology Reviews* **2015**, *64*, 626-641. DOI: 10.1016/j.oregeorev.2014.03.010.
(707) Mitra, A.; Chatterjee, S.; Moogouei, R.; Gupta, D. K. Arsenic Accumulation in Rice and Probable Mitigation Approaches: A Review. *Agronomy-Basel* **2017**, *7* (4), 67. DOI: 10.3390/agronomy7040067.
(708) Miyashita, S.I.; Murota, C.; Kondo, K.; Fujiwara, S.; Tsuzuki, M. Arsenic metabolism in cyanobacteria. *Environmental Chemistry* **2015**, *13*, 577-589.
(709) Miyata, N.; Tani, Y.; Sakata, M.; Iwahori, K. Microbial manganese oxide formation and interaction with toxic metal ions. *J Biosci Bioeng* **2007**, *104* (1), 1-8. DOI: 10.1263/jbb.104.1.
(710) Mmelesi, O. K.; Masunga, N.; Kuvarega, A.; Nkambule, T. T.; Mamba, B. B.; Kefeni, K. K. Cobalt ferrite nanoparticles and nanocomposites: Photocatalytic, antimicrobial activity and toxicity in water treatment. *Materials Science in Semiconductor Processing* **2021**, *123*, 105523.
(711) Mohan, D.; Pittman, C. U., Jr. Arsenic removal from water/wastewater using adsorbents--A critical review. *J Hazard Mater* **2007**, *142* (1-2), 1-53. DOI: 10.1016/j.jhazmat.2007.01.006.
(712) Mohanty, M.; Patra, H. K. Attenuation of chromium toxicity by bioremediation technology. *Rev Environ Contam Toxicol* **2011**, *210*, 1-34. DOI: 10.1007/978-1-4419-7615-4_1.
(713) Mohanty, S.; Ghosh, S.; Bal, B.; Das, A. P. A review of biotechnology processes applied for manganese recovery from wastes. *Reviews in Environmental Science and Bio-Technology* **2018**, *17* (4), 791-811. DOI: 10.1007/s11157-018-9482-1.
(714) Mompean, F.J.; Perrone, J.; Illemassène, M. Chemical thermodynamics of zirconium. **2005**, 544 pp.
(715) Mondal, P.; Chattopadhyay, A. Environmental exposure of arsenic and fluoride and their combined toxicity: a recent update. *Journal of Applied Toxicology* **2020**, *40*, 552-566.
(716) Mondal, M. K.; Garg, R. A comprehensive review on removal of arsenic using activated carbon prepared from easily available waste materials. *Environ Sci Pollut Res Int* **2017**, *24* (15), 13295-13306. DOI: 10.1007/s11356-017-8842-7.
(717) Morachevskii, A. G. Lithium-Tellurium System: Thermodynamic and Electrochemical Studies and Prospects for Use in Chemical Current Sources. *Russian Journal of Applied Chemistry* **2020**, *93* (3), 313-324. DOI: 10.1134/S1070427220030015.

(718) Moradi, B.; Botte, G. G. Recycling of graphite anodes for the next generation of lithium ion batteries. *Journal of Applied Electrochemistry* **2016**, *46* (2), 123-148. DOI: 10.1007/s10800-015-0914-0.
(719) Mori, T.; Ou, D. R.; Zou, J.; Drennan, J. Present status and future prospect of design of Pt-cerium oxide electrodes for fuel cell applications. *Progress in Natural Science-Materials International* **2012**, *22* (6), 561-571. DOI: 10.1016/j.pnsc.2012.11.010.
(720) Mossali, E.; Picone, N.; Gentilini, L.; Rodriguez, O.; Perez, J. M.; Colledani, M. Lithium-ion batteries towards circular economy: A literature review of opportunities and issues of recycling treatments. *J Environ Manage* **2020**, *264*, 110500. DOI: 10.1016/j.jenvman.2020.110500.
(721) Motoi, Y.; Shimada, K.; Ishiguro, K.; Hattori, N. Lithium and autophagy. *ACS Chem Neurosci* **2014**, *5* (6), 434-442. DOI: 10.1021/cn500056q.
(722) Moynier, F.; Vance, D.; Fujii, T.; Savage, P. The Isotope Geochemistry of Zinc and Copper. *Non-Traditional Stable Isotopes* **2017**, *82*, 543-+. DOI: 10.2138/rmg.2017.82.13.
(723) Mpinga, C. N.; Eksteen, J. J.; Aldrich, C.; Dyer, L. Direct leach approaches to Platinum Group Metal (PGM) ores and concentrates: A review. *Minerals Engineering* **2015**, *78*, 93-113. DOI: 10.1016/j.mineng.2015.04.015.
(724) Mubarak, H.; Chai, L. Y.; Mirza, N.; Yang, Z. H.; Pervez, A.; Tariq, M.; Shaheen, S.; Mahmood, Q. Antimony (Sb) - pollution and removal techniques - critical assessment of technologies. *Toxicological and Environmental Chemistry* **2015**, *97* (10), 1296-1318. DOI: 10.1080/02772248.2015.1095549.
(725) Mudd, G. M. Global trends and environmental issues in nickel mining: Sulfides versus laterites. *Ore Geology Reviews* **2010**, *38* (1-2), 9-26. DOI: 10.1016/j.oregeorev.2010.05.003.
(726) Mudd, G. M. Key trends in the resource sustainability of platinum group elements. *Ore Geology Reviews* **2012**, *46*, 106-117. DOI: 10.1016/j.oregeorev.2012.02.005.
(727) Mudd, G. M.; Weng, Z.; Jowitt, S. M.; Turnbull, I. D.; Graedel, T. E. Quantifying the recoverable resources of by-product metals: The case of cobalt. *Ore Geology Reviews* **2013**, *55*, 87-98. DOI: 10.1016/j.oregeorev.2013.04.010.
(728) Muhammad, N.; Zvobgo, G.; Zhang, G. P. A review: The beneficial effects and possible mechanisms of aluminum on plant growth in acidic soil. *Journal of Integrative Agriculture* **2019**, *18* (7), 1518-1528. DOI: 10.1016/S2095-3119(18)61991-4.
(729) Mukherjee, B.; Patra, B.; Mahapatra, S.; Banerjee, P.; Tiwari, A.; Chatterjee, M. Vanadium--an element of atypical biological significance. *Toxicol Lett* **2004**, *150* (2), 135-143. DOI: 10.1016/j.toxlet.2004.01.009.
(730) Mukhopadhyay, M. J.; Sharma, A. Manganese in Cell-Metabolism of Higher-Plants. *Botanical Review* **1991**, *57* (2), 117-149. DOI: 10.1007/bf02858767.
(731) Mulrooney, S. B.; Hausinger, R. P. Nickel uptake and utilization by microorganisms. *FEMS Microbiol Rev* **2003**, *27* (2-3), 239-261. DOI: 10.1016/S0168-6445(03)00042-1.
(732) Multani, R. S.; Feldmann, T.; Demopoulos, G. P. Antimony in the metallurgical industry: A review of its chemistry and environmental stabilization options. *Hydrometallurgy* **2016**, *164*, 141-153. DOI: 10.1016/j.hydromet.2016.06.014.
(733) München, D. D.; Veit, H. M. Neodymium as the main feature of permanent magnets from hard disk drives (HDDs). *Waste management* **2017**, *61*, 372-376.
(734) Munoz, A.; Costa, M. Elucidating the mechanisms of nickel compound uptake: a review of particulate and nano-nickel endocytosis and toxicity. *Toxicol Appl Pharmacol* **2012**, *260* (1), 1-16. DOI: 10.1016/j.taap.2011.12.014.

(735) Murao, S.; Furuno, M.; Uchida, A. C. Geology of indium deposits-a review. *Mining Geology* **1991**, *41*, 1-13.
(736) Murao, S.; Deb, M.; Furuno, M. Mineralogical evolution of indium in high grade tin-polymetallic hydrothermal veins - A comparative study from Tosham, Haryana state, India and Goka, Naegi district, Japan. *Ore Geology Reviews* **2008**, *33* (3-4), 490-504. DOI: 10.1016/j.oregeorev.2007.02.004.
(737) Murphy, P. D.; Thomas, D. J. The evolution of Late Cretaceous deep-ocean circulation in the Atlantic basins: Neodymium isotope evidence from South Atlantic drill sites for tectonic controls. *Geochemistry, Geophysics, Geosystems* **2013**, *14*, 5323-5340.
(738) Musial, J.; Krakowiak, R.; Mlynarczyk, D. T.; Goslinski, T.; Stanisz, B. J. Titanium dioxide nanoparticles in food and personal care products—What do we know about their safety? *Nanomaterials* **2020**, *10*, 1110.
(739) Nabi, A.; Naeem, M.; Aftab, T.; Khan, M. M. A.; Ahmad, P. A comprehensive review of adaptations in plants under arsenic toxicity: Physiological, metabolic and molecular interventions. *Environmental Pollution* **2021**, *290,* 118029. DOI: 10.1016/j.envpol.2021.118029.
(740) Nadeem, M.; Khan, R.; Afridi, K.; Nadhman, A.; Ullah, S.; Faisal, S.; Ul Mabood, Z.; Hano, C.; Abbasi, B. H. Green Synthesis of Cerium Oxide Nanoparticles (CeO2 NPs) and Their Antimicrobial Applications: A Review. *International Journal of Nanomedicine* **2020**, *15*, 5951-5961. DOI: 10.2147/Ijn.S255784.
(741) Naeem, A.; Aslam, M.; Saifullah; Muhling, K. H. Lithium: Perspectives of nutritional beneficence, dietary intake, biogeochemistry, and biofortification of vegetables and mushrooms. *Sci Total Environ* **2021**, *798*, 149249. DOI: 10.1016/j.scitotenv.2021.149249.
(742) Naglav, D.; Buchner, M. R.; Bendt, G.; Kraus, F.; Schulz, S. Off the Beaten Track-A Hitchhiker's Guide to Beryllium Chemistry. *Angew Chem Int Ed Engl* **2016**, *55* (36), 10562-10576. DOI: 10.1002/anie.201601809.
(743) Nakamaru, Y. M.; Altansuvd, J. Speciation and bioavailability of selenium and antimony in non-flooded and wetland soils: a review. *Chemosphere* **2014**, *111*, 366-371. DOI: 10.1016/j.chemosphere.2014.04.024.
(744) Nam, D. H.; Lee, B. C.; Eom, I. C.; Kim, P.; Yeo, M. K. Uptake and bioaccumulation of titanium- and silver-nanoparticles in aquatic ecosystems. *Molecular & Cellular Toxicology* **2014**, *10* (1), 9-17. DOI: 10.1007/s13273-014-0002-2.
(745) Namiesnik, J.; Rabajczyk, A. The Speciation of Aluminum in Environmental Samples. *Critical Reviews in Analytical Chemistry* **2010**, *40* (2), 68-88. DOI: 10.1080/10408340903153234.
(746) Namiesnik, J.; Rabajczyk, A. Speciation Analysis of Chromium in Environmental Samples. *Critical Reviews in Environmental Science and Technology* **2012**, *42* (4), 327-377. DOI: 10.1080/10643389.2010.518517.
(747) Narayani, M.; Shetty, K. V. Chromium-Resistant Bacteria and Their Environmental Condition for Hexavalent Chromium Removal: A Review. *Critical Reviews in Environmental Science and Technology* **2013**, *43* (9), 955-1009. DOI: 10.1080/10643389.2011.627022.
(748) Nash, J. T. Volcanogenic uranium deposits—Geology, geochemical processes, and criteria for resource assessment: U.S. Geological Survey Open-File Report 2010-1001, **2010**, 99 p.
(749) Nash, M. J.; Maskall, J. E.; Hill, S. J. Methodologies for determination of antimony in terrestrial environmental samples. *J Environ Monit* **2000**, *2* (2), 97-109. DOI: 10.1039/a907875d.

(750) Natasha; Shahid, M.; Khalid, S.; Dumat, C.; Pierart, A.; Niazi, N. K. Biogeochemistry of antimony in soil-plant system: Ecotoxicology and human health. *Applied Geochemistry* **2019**, *106*, 45-59. DOI: 10.1016/j.apgeochem.2019.04.006.
(751) Natasha, N.; Shahid, M.; Bibi, I.; Iqbal, J.; Khalid, S.; Murtaza, B.; Bakhat, H. F.; Farooq, A. B. U.; Amjad, M.; Hammad, H. M.; Niazi, N. K.; Arshad, M. Zinc in soil-plant-human system: A data-analysis review. *Sci Total Environ* **2022**, *808*, 152024. DOI: 10.1016/j.scitotenv.2021.152024.
(752) Navarro, J.; Zhao, F. Life-cycle assessment of the production of rare-earth elements for energy applications: a review. *Frontiers in Energy Research* **2014**, *2,* 45. DOI: 10.3389/fenrg.2014.00045.
(753) Nayak, A.; Jena, M. S.; Mandre, N. R. Beneficiation of Lead-Zinc Ores - A Review. *Mineral Processing and Extractive Metallurgy Review* **2022**, *43* (5), 564-583. DOI: 10.1080/08827508.2021.1903459.
(754) Nayak, P. Aluminum: impacts and disease. *Environ Res* **2002**, *89* (2), 101-115. DOI: 10.1006/enrs.2002.4352.
(755) Neculita, C. M.; Rosa, E. A review of the implications and challenges of manganese removal from mine drainage. *Chemosphere* **2019**, *214*, 491-510. DOI: 10.1016/j.chemosphere.2018.09.106.
(756) Neff, J. M. Ecotoxicology of arsenic in the marine environment. *Environmental Toxicology and Chemistry* **1997**, *16* (5), 917-927. DOI: 10.1002/etc.5620160511.
(757) Newsome, L.; Morris, K.; Lloyd, J. R. The biogeochemistry and bioremediation of uranium and other priority radionuclides. *Chemical Geology* **2014**, *363*, 164-184. DOI: 10.1016/j.chemgeo.2013.10.034.
(758) Ng, J. C. Environmental contamination of arsenic and its toxicological impact on humans. *Environmental Chemistry* **2005**, *2* (3), 146-160. DOI: 10.1071/En05062.
(759) Nguyen, T. H.; Lee, M. S. A review on separation of gallium and indium from leach liquors by solvent extraction and ion exchange. *Mineral Processing and Extractive Metallurgy Review* **2018**, *40*, 278-291.
(760) Nguyen, T. H.; Lee, M. S. A review on the separation of niobium and tantalum by solvent extraction. *Mineral Processing and Extractive Metallurgy Review* **2018**, *40*, 265-277.
(761) Nguyen, T. H.; Lee, M. S. A Review on the Recovery of Titanium Dioxide from Ilmenite Ores by Direct Leaching Technologies. *Mineral Processing and Extractive Metallurgy Review* **2019**, *40* (4), 231-247. DOI: 10.1080/08827508.2018.1502668.
(762) Nguyen, T. H.; Lee, M. S. A Review on Germanium Resources and its Extraction by Hydrometallurgical Method. *Mineral Processing and Extractive Metallurgy Review* **2021**, *42* (6), 406-426. DOI: 10.1080/08827508.2020.1756795.
(763) Nicholas, D. R.; Ramamoorthy, S.; Palace, V.; Spring, S.; Moore, J. N.; Rosenzweig, R. F. Biogeochemical transformations of arsenic in circumneutral freshwater sediments. *Biodegradation* **2003**, *14* (2), 123-137. DOI: 10.1023/a:1024031700533.
(764) Nicholson, K.; Hein, J. R.; Buhn, B.; Dasgupta, S. (Eds.) Manganese mineralization: Geochemistry and mineralogy of terrestrial and marine deposits. *Geological Society of London, Special Publication No. 19.* **1997**, 370 pp.
(765) Nielsen, F. H. Vanadium in mammalian physiology and nutrition. *Metal Ions in Biological Systems* **1995**, *31*, 543-573.

(766) Nishad, P. A.; Bhaskarapillai, A. Antimony, a pollutant of emerging concern: A review on industrial sources and remediation technologies. *Chemosphere* **2021**, *277*, 130252. DOI: 10.1016/j.chemosphere.2021.130252.
(767) Nordstrom, D. K.; Majzlan, J.; Konigsberger, E. Thermodynamic Properties for Arsenic Minerals and Aqueous Species. *Arsenic: Environmental Geochemistry, Mineralogy, and Microbiology* **2014**, *79*, 217-255. DOI: 10.2138/rmg.2014.79.4.
(768) Noulas, C.; Tziouvalekas, M.; Karyotis, T. Zinc in soils, water and food crops. *J Trace Elem Med Biol* **2018**, *49*, 252-260. DOI: 10.1016/j.jtemb.2018.02.009.
(769) Oberthür, T. The fate of platinum-group minerals in the exogenic environment—From sulfide ores via oxidized ores into placers: Case studies bushveld complex, South Africa, and Great Dyke, Zimbabwe. *Minerals* **2018**, *8*, 581.
(770) Obiakor, M. O.; Tighe, M.; Pereg, L.; Wilson, S. C. Bioaccumulation, trophodynamics and ecotoxicity of antimony in environmental freshwater food webs. *Critical Reviews in Environmental Science and Technology* **2017**, *47* (22), 2208-2258. DOI: 10.1080/10643389.2017.1419790.
(771) Ogra, Y. Biology and toxicology of tellurium explored by speciation analysis. *Metallomics* **2017**, *9* (5), 435-441. DOI: 10.1039/c7mt00022g.
(772) Ojima, K. Aluminum toxicity and tolerance in plant roots. *Seikagaku. The Journal of Japanese Biochemical Society* **1989**, *61*, 34-38. DOI: 10.1039/c1mt00056j
(774) Oliveira, d.; P., R.; Benvenuti, J.; Espinosa., D. C. R. A review of the current progress in recycling technologies for gallium and rare earth elements from light-emitting diodes. *Renewable and Sustainable Energy Reviews* **2021**, *145*.
(775) Oliverio, M.; Nardi, M.; Costanzo, P.; Di Gioia, M. L.; Procopio, A. Erbium Salts as Non-Toxic Catalysts Compatible with Alternative Reaction Media. *Sustainability* **2018**, *10* (3), 721. DOI: 10.3390/su10030721.
(776) Omodara, L.; Pitkaaho, S.; Turpeinen, E. M.; Saavalainen, P.; Oravisjarvi, K.; Keiski, R. L. Recycling and substitution of light rare earth elements, cerium, lanthanum, neodymium, and praseodymium from end-of-life applications - A review. *J Clean Prod* **2019**, *236,* 117573. DOI: 10.1016/j.jclepro.2019.07.048.
(777) Ovchinnikov, N.L.; Solodov, N. A. Genetic types of cesium deposits and some problems of their exploration. *International Geology Review* **1972**, *14*, 707-719.
(778) Oze, C.; Fendorf, S.; Bird, D. K.; Coleman, R. G. Chromium geochemistry in serpentinized ultramafic rocks and serpentine soils from the Franciscan Complex of California. *American Journal of Science* **2004**, *304* (1), 67-101. DOI: 10.2475/ajs.304.1.67.
(779) Ozsvath, D L. Fluoride and environmental health: a review. *Reviews in Environmental Science and Bio/Technology* **2009**, *8*, 59-79.
(780) Pagano, G.; Aliberti, F.; Guida, M.; Oral, R.; Siciliano, A.; Trifuoggi, M.; Tommasi, F. Rare earth elements in human and animal health: State of art and research priorities. *Environ Res* **2015**, *142*, 215-220. DOI: 10.1016/j.envres.2015.06.039.
(781) Pagano, G.; Thomas, P. J.; Di Nunzio, A.; Trifuoggi, M. Human exposures to rare earth elements: Present knowledge and research prospects. *Environ Res* **2019**, *171*, 493-500. DOI: 10.1016/j.envres.2019.02.004.
(782) Paikaray, S. Environmental hazards of arsenic associated with black shales: a review on geochemistry, enrichment and leaching mechanism. *Reviews in Environmental Science and Bio-Technology* **2012**, *11* (3), 289-303. DOI: 10.1007/s11157-012-9281-z.

(783) Paikaray, S. Arsenic Geochemistry of Acid Mine Drainage. *Mine Water and the Environment* **2015**, *34* (2), 181-196. DOI: 10.1007/s10230-014-0286-4.
(784) Panda, S.; Costa, R. B.; Shah, S. S.; Mishra, S.; Bevilaqua, D.; Akcil, A. Biotechnological trends and market impact on the recovery of rare earth elements from bauxite residue (red mud) - A review. *Resour Conserv Recy* **2021**, *171,* 105645. DOI: 10.1016/j.resconrec.2021.105645.
(785) Panda, S. K.; Matsumoto, H. Molecular physiology of aluminum toxicity and tolerance in plants. *Botanical Review* **2007**, *73* (4), 326-347. DOI: 10.1663/0006-8101(2007)73[326:Mpoata]2.0.Co;2.
(786) Pandurangan, M.; Kim, D. H. In vitro toxicity of zinc oxide nanoparticles: a review. *Journal of Nanoparticle Research* **2015**, *17* (3), 158. DOI: 10.1007/s11051-015-2958-9.
(787) Panichev; M., A. Rare earth elements: review of medical and biological properties and their abundance in the rock materials and mineralized spring waters in the context of animal and human geophagia reasons evaluation. *Achievements in the life sciences* **2015**, *9*.
(788) Papaiconomou, N.; Lee, J. M.; Salminen, J.; von Stosch, M.; Prausnitz, J. M. Selective extraction of copper, mercury, silver, and palladium ions from water using hydrophobic ionic liquids. *Industrial & Engineering Chemistry Research* **2008**, *47* (15), 5080-5086. DOI: 10.1021/ie0706562.
(789) Park, M. S.; Kim, J. G.; Kim, Y. J.; Choi, N. S.; Kim, J. S. Recent Advances in Rechargeable Magnesium Battery Technology: A Review of the Field's Current Status and Prospects. *Israel Journal of Chemistry* **2015**, *55* (5), 570-585. DOI: 10.1002/ijch.201400174.
(790) Park, S. M.; Alessi, D. S.; Baek, K. Selective adsorption and irreversible fixation behavior of cesium onto 2:1 layered clay mineral: A mini review. *J Hazard Mater* **2019**, *369*, 569-576. DOI: 10.1016/j.jhazmat.2019.02.061.
(791) Park, S. M.; Kim, J. G.; Kim, H. B.; Kim, Y. H.; Baek, K. Desorption technologies for remediation of cesium-contaminated soils: a short review. *Environ Geochem Health* **2021**, *43* (9), 3263-3272. DOI: 10.1007/s10653-020-00667-3.
(792) Park, Y. J.; Kim, M. K.; Kim, H. S.; Lee, B. M. Risk assessment of lithium-ion battery explosion: chemical leakages. *J Toxicol Environ Health B Crit Rev* **2018**, *21* (6-8), 370-381. DOI: 10.1080/10937404.2019.1601815.
(793) Parker, V. B. Thermodynamic Properties of the Aqueous Ba2+ Ion and the Key Compounds of Barium. *Journal of Physical and Chemical Reference Data* **1995**, *24* (2), 1023-1036. DOI: 10.1063/1.555973.
(794) Patel, M.; Karamalidis, A. K. Germanium: A review of its US demand, uses, resources, chemistry, and separation technologies. *Separation and Purification Technology* **2021**, *275,* 118981. DOI: 10.1016/j.seppur.2021.118981.
(795) Pathan, S.; Bose, S. Biopolymer based hydrogels for arsenic removal. *Current Science* **2020**, *118* (10), 1540-1546.
(796) Paustenbach, D. J.; Tvermoes, B. E.; Unice, K. M.; Finley, B. L.; Kerger, B. D. A review of the health hazards posed by cobalt. *Crit Rev Toxicol* **2013**, *43* (4), 316-362. DOI: 10.3109/10408444.2013.779633.
(797) Pavesi, T.; Moreira, J. C. Mechanisms and individuality in chromium toxicity in humans. *J Appl Toxicol* **2020**, *40* (9), 1183-1197. DOI: 10.1002/jat.3965.
(798) Pawlak, J.; Lodyga-Chruscinska, E.; Chrustowicz, J. Fate of platinum metals in the environment. *J Trace Elem Med Biol* **2014**, *28* (3), 247-254. DOI: 10.1016/j.jtemb.2014.03.005.

(799) Peana, M.; Medici, S.; Dadar, M.; Zoroddu, M. A.; Pelucelli, A.; Chasapis, C. T.; Bjorklund, G. Environmental barium: potential exposure and health-hazards. *Arch Toxicol* **2021**, *95* (8), 2605-2612. DOI: 10.1007/s00204-021-03049-5.
(800) Pearce, J. Studies of Any Toxicological Effects of Prussian-Blue Compounds in Mammals - a Review. *Food and Chemical Toxicology* **1994**, *32* (6), 577-582. DOI: 10.1016/0278-6915(94)90116-3.
(801) Peng, H. A literature review on leaching and recovery of vanadium. *Journal of Environmental Chemical Engineering* **2019**, *7* (5), 103313. DOI: 10.1016/j.jece.2019.103313.
(802) Peng, H.; Guo, J. Removal of chromium from wastewater by membrane filtration, chemical precipitation, ion exchange, adsorption electrocoagulation, electrochemical reduction, electrodialysis, electrodeionization, photocatalysis and nanotechnology: a review. *Environmental Chemistry Letters* **2020**, *18* (6), 2055-2068. DOI: 10.1007/s10311-020-01058-x.
(803) Peng, Z. M.; Yang, H. Designer platinum nanoparticles: Control of shape, composition in alloy, nanostructure and electrocatalytic property. *Nano Today* **2009**, *4* (2), 143-164. DOI: 10.1016/j.nantod.2008.10.010.
(804) Pereao, O.; Bode-Aluko, C.; Fatoba, O.; Laatikainen, K.; Petrik, L. Rare earth elements removal techniques from water/wastewater: a review. *Desalination and Water Treatment* **2018**, *130*, 71-86. DOI: 10.5004/dwt.2018.22844.
(805) Pereira-Pardo, L.; Korenberg, C. The use of erbium lasers for the conservation of cultural heritage. A review. *Journal of Cultural Heritage* **2018**, *31*, 236-247. DOI: 10.1016/j.culher.2017.10.007.
(806) Pereiro, I. R.; Diaz, A. C. Speciation of mercury, tin, and lead compounds by gas chromatography with microwave-induced plasma and atomic-emission detection (GC-MIP-AED). *Anal Bioanal Chem* **2002**, *372* (1), 74-90. DOI: 10.1007/s00216-001-1139-0.
(807) Perera, L. C.; Raymond, O.; Henderson, W.; Brothers, P. J.; Plieger, P. G. Advances in beryllium coordination chemistry. *Coordination Chemistry Reviews* **2017**, *352*, 264-290. DOI: 10.1016/j.ccr.2017.09.009.
(808) Peric, T. S.; Jankovic, S. M. Cardiotoxicity of Palladium Compounds. *Journal of Medical Biochemistry* **2013**, *32* (1), 20-25. DOI: 10.2478/v10011-012-0010-5.
(809) Perks, C.; Mudd, G. Titanium, zirconium resources and production: A state of the art literature review. *Ore Geology Reviews* **2019**, *107*, 629-646. DOI: 10.1016/j.oregeorev.2019.02.025.
(810) Perl, D. P.; Moalem, S. Aluminum, Alzheimer's disease and the geospatial occurrence of similar disorders. *Medical Mineraology and Geochemistry* **2006**, *64*, 115-134. DOI: 10.2138/rmg.2006.64.4.
(811) Peters, G. R.; McCurdy, R. F.; Hindmarsh, J. T. Environmental aspects of arsenic toxicity. *Crit Rev Clin Lab Sci* **1996**, *33* (6), 457-493. DOI: 10.3109/10408369609080055.
(812) Petersen, E. U. Tin in Volcanogenic Massive Sulfide Deposits - an Example from the Geco Mine, Manitouwadge District, Ontario, Canada. *Economic Geology* **1986**, *81* (2), 323-342. DOI: DOI 10.2113/gsecongeo.81.2.323.
(813) Peucker-Ehrenbrink, B.; Ravizza, G. The marine osmium isotope record. *Terra Nova* **2000**, *12* (5), 205-219. DOI: DOI 10.1046/j.1365-3121.2000.00295.x.
(814) Phillips, D. J. H. Arsenic in Aquatic Organisms - a Review, Emphasizing Chemical Speciation. *Aquatic Toxicology* **1990**, *16* (3), 151-186. DOI: 10.1016/0166-445x(90)90036-O.

(815) Pierart, A.; Shahid, M.; Sejalon-Delmas, N.; Dumat, C. Antimony bioavailability: knowledge and research perspectives for sustainable agricultures. *J Hazard Mater* **2015**, *289*, 219-234. DOI: 10.1016/j.jhazmat.2015.02.011.
(816) Plum, L. M.; Rink, L.; Haase, H. The essential toxin: impact of zinc on human health. *Int J Environ Res Public Health* **2010**, *7* (4), 1342-1365. DOI: 10.3390/ijerph7041342.
(817) Poljsak, B.; Pocsi, I.; Raspor, P.; Pesti, M. Interference of chromium with biological systems in yeasts and fungi: a review. *J Basic Microbiol* **2010**, *50* (1), 21-36. DOI: 10.1002/jobm.200900170.
(818) Porcelli, D.; Ballentine, C. J.; Wieler, R. An overview of noble gas - Geochemistry and cosmochemistry. *Noble Gases in Geochemistry and Cosmochemistry* **2002**, *47*, 1-19. DOI: 10.2138/rmg.2002.47.1.
(819) Port, M.; Idee, J. M.; Medina, C.; Robic, C.; Sabatou, M.; Corot, C. Efficiency, thermodynamic and kinetic stability of marketed gadolinium chelates and their possible clinical consequences: a critical review. *Biometals* **2008**, *21* (4), 469-490. DOI: 10.1007/s10534-008-9135-x.
(820) Poschenrieder, C.; Gunse, B.; Corrales, I.; Barcelo, J. A glance into aluminum toxicity and resistance in plants. *Science of the Total Environment* **2008**, *400* (1-3), 356-368. DOI: 10.1016/j.scitotenv.2008.06.003.
(821) Powell, K. J.; Brown, P. L.; Byrne, R. H.; Gajda, T.; Hefter, G.; Leuz, A. K.; Sjoberg, S.; Wanner, H. Chemical speciation of environmentally significant metals with inorganic ligands. Part 5: The Zn2+ + OH-, Cl-, CO32-, SO42-, and PO43-systems (IUPAC Technical Report). *Pure and Applied Chemistry* **2013**, *85* (12), 2249-2311. DOI: 10.1351/Pac-Rep-13-06-03.
(822) Pradhan, D.; Panda, S.; Sukla, L. B. Recent advances in indium metallurgy: A review. *Mineral Processing and Extractive Metallurgy Review* **2018**, *39* (3), 167-180. DOI: 10.1080/08827508.2017.1399887.
(823) Presentato, A.; Turner, R. J.; Vasquez, C. C.; Yurkov, V.; Zannoni, D. Tellurite-dependent blackening of bacteria emerges from the dark ages. *Environmental Chemistry* **2019**, *16* (4), 266-288. DOI: 10.1071/En18238.
(824) Prystupa, J. Fluorine--a current literature review. An NRC and ATSDR based review of safety standards for exposure to fluorine and fluorides. *Toxicol Mech Methods* **2011**, *21* (2), 103-170. DOI: 10.3109/15376516.2010.542931.
(825) Pushkar, B.; Sevak, P.; Parab, S.; Nilkanth, N. Chromium pollution and its bioremediation mechanisms in bacteria: A review. *J Environ Manage* **2021**, *287*, 112279. DOI: 10.1016/j.jenvman.2021.112279.
(826) Pyrzyñska, K. Selected problems in speciation analysis of vanadium in water samples. *Chemia analityczna* **2006**, *51*, 339-350.
(827) Pyrzynska, K.; Kilian, K.; Pegier, M. Separation and purification of scandium: From industry to medicine. *Separation and Purification Reviews* **2019**, *48* (1), 65-77. DOI: 10.1080/15422119.2018.1430589.
(828) Pyrzynska, K.; Wierzbicki, T. Determination of vanadium species in environmental samples. *Talanta* **2004**, *64* (4), 823-829. DOI: 10.1016/j.talanta.2004.05.007.
(829) Qadir, M.; Schubert, S.; Oster, J. D.; Sposito, G.; Minhas, P. S.; Cheraghi, S. A. M.; Murtaza, G.; Mirzabaev, A.; Saqib, M. High‑magnesium waters and soils: Emerging environmental and food security constraints. *Sci Total Environ* **2018**, *642*, 1108-1117. DOI: 10.1016/j.scitotenv.2018.06.090.

(830) Qi, C.; Liu, G.; Chou, C. L.; Zheng, L. Environmental geochemistry of antimony in Chinese coals. *Sci Total Environ* **2008**, *389* (2-3), 225-234. DOI: 10.1016/j.scitotenv.2007.09.007.
(831) Qi, P. F.; Wang, Y.; Zeng, J. Q.; Sui, K. Y.; Zhao, J. A. Progress in antimony capturing by superior materials: Mechanisms, properties and perspectives. *Chemical Engineering Journal* **2021**, *419,* 130013. DOI: 10.1016/j.cej.2021.130013.
(832) Qi, S. H.; Wu, D. X.; Dong, Y.; Liao, J. Q.; Foster, C. W.; O'Dwyer, C.; Feng, Y. Z.; Liu, C. T.; Ma, J. M. Cobalt-based electrode materials for sodium-ion batteries. *Chemical Engineering Journal* **2019**, *370*, 185-207. DOI: 10.1016/j.cej.2019.03.166.
(833) Qin, L. P.; Wang, X. L. Chromium Isotope Geochemistry. *Non-Traditional Stable Isotopes* **2017**, *82*, 379-414. DOI: 10.2138/rmg.2017.82.10.
(834) Qin, S. J.; Zhao, C. L.; Li, Y. H.; Zhang, Y. Review of coal as a promising source of lithium. *International Journal of Oil Gas and Coal Technology* **2015**, *9* (2), 215-229. DOI: 10.1504/Ijogct.2015.067490.
(835) Quast, K.; Addai-Mensah, J.; Skinner, W. Preconcentration strategies in the processing of nickel laterite ores Part 5: Effect of mineralogy. *Minerals Engineering* **2017**, *110*, 31-39. DOI: 10.1016/j.mineng.2017.03.012.
(836) Quast, K.; Connor, J. N.; Skinner, W.; Robinson, D. J.; Addai-Mensah, J. Preconcentration strategies in the processing of nickel laterite ores Part 1: Literature review. *Minerals Engineering* **2015**, *79*, 261-268. DOI: 10.1016/j.mineng.2015.03.017.
(837) Quast, K.; Connor, J. N.; Skinner, W.; Robinson, D. J.; Li, J.; Addai-Mensah, J. Preconcentration strategies in the processing of nickel laterite ores part 2: Laboratory experiments. *Minerals Engineering* **2015**, *79*, 269-278. DOI: 10.1016/j.mineng.2015.03.016.
(838) Quast, K.; Otsuki, A.; Fornasiero, D.; Robinson, D. J.; Addai-Mensah, J. Preconcentration strategies in the processing of nickel laterite ores part 3: Flotation testing. *Minerals Engineering* **2015**, *79*, 279-286. DOI: 10.1016/j.mineng.2015.03.018.
(839) Rahman, M. A.; Lee, S. H.; Ji, H. C.; Kabir, A. H.; Jones, C. S.; Lee, K. W. Importance of Mineral Nutrition for Mitigating Aluminum Toxicity in Plants on Acidic Soils: Current Status and Opportunities. *Int J Mol Sci* **2018**, *19* (10). DOI: 10.3390/ijms19103073.
(840) Rahman, M. M.; Ng, J. C.; Naidu, R. Chronic exposure of arsenic via drinking water and its adverse health impacts on humans. *Environ Geochem Health* **2009**, *31 Suppl 1*, 189-200. DOI: 10.1007/s10653-008-9235-0.
(841) Rajakumar, G.; Mao, L. B.; Bao, T.; Wen, W.; Wang, S. F.; Gomathi, T.; Gnanasundaram, N.; Rebezov, M.; Shariati, M. A.; Chung, I. M.; Thiruvengadam, M.; Zhang, X. H. Yttrium Oxide Nanoparticle Synthesis: An Overview of Methods of Preparation and Biomedical Applications. *Applied Sciences-Basel* **2021**, *11* (5), 2172. DOI: 10.3390/app11052172.
(842) Rajwanshi, P.; Singh, V.; Gupta, M. K.; Dass, S. Leaching of aluminium from cookwares - A review. *Environmental Geochemistry and Health* **1997**, *19* (1), 1-18.
(843) Rakhunde, R.; Deshpande, L.; Juneja, H. D. Chemical Speciation of Chromium in Water: A Review. *Critical Reviews in Environmental Science and Technology* **2012**, *42* (7), 776-810. DOI: 10.1080/10643389.2010.534029.
(844) Rao, C. R. M.; Reddi, G. S. Platinum group metals (PGM); occurrence, use and recent trends in their determination. *Trac-Trends in Analytical Chemistry* **2000**, *19* (9), 565-586. DOI: 10.1016/S0165-9936(00)00031-5.
(845) Rao, K.; N.; Sreenivas., T. Beryllium—Geochemistry, Mineralogy and Beneficiation. *Mineral Processing and Extractive Metullargy Review* **1994**, *13*.

(846) Rard, J. A. Chemistry and Thermodynamics of Europium and Some of Its Simpler Inorganic-Compounds and Aqueous Species. *Chemical Reviews* **1985**, *85* (6), 555-582. DOI: DOI 10.1021/cr00070a003.
(847) Rasheed; A. A review on aluminum toxicity and quantitative trait loci maping in rice (Oryza sativa L). *Applied Ecology and Environmental Research* **2020**, *18*.
(848) Rasheed, H.; Slack, R.; Kay, P. Human health risk assessment for arsenic: A critical review. *Critical Reviews in Environmental Science and Technology* **2016**, *46* (19-20), 1529-1583. DOI: 10.1080/10643389.2016.1245551.
(849) Rashid, M. M.; Forte Tavcer, P.; Tomsic, B. Influence of Titanium Dioxide Nanoparticles on Human Health and the Environment. *Nanomaterials (Basel)* **2021**, *11* (9). DOI: 10.3390/nano11092354.
(850) Rasoulnia, P.; Barthen, R.; Lakaniemi, A. A critical review of bioleaching of rare earth elements: The mechanisms and effect of process parameters. *Critical Reviews in Environmental Science and Technology* **2021**, *51*, 378-427. DOI: 10.1080/10643389.2020.1727718
(851) Rathi, B. S.; Kumar, P. S. A review on sources, identification and treatment strategies for the removal of toxic Arsenic from water system. *Journal of Hazardous Materials* **2021**, *418,* 126299. DOI: 10.1016/j.jhazmat.2021.126299.
(852) Rauwel, P.; Rauwel, E. Towards the Extraction of Radioactive Cesium-137 from Water via Graphene/CNT and Nanostructured Prussian Blue Hybrid Nanocomposites: A Review. *Nanomaterials (Basel)* **2019**, *9* (5). DOI: 10.3390/nano9050682.
(853) Raval, N. P.; Shah, P. U.; Shah, N. K. Adsorptive removal of nickel(II) ions from aqueous environment: A review. *J Environ Manage* **2016**, *179*, 1-20. DOI: 10.1016/j.jenvman.2016.04.045.
(854) Ravindra, K.; Bencs, L.; Van Grieken, R. Platinum group elements in the environment and their health risk. *Science of the Total Environment* **2004**, *318* (1-3), 1-43. DOI: 10.1016/S0048-9697(03)00372-3.
(855) Rawashdeh, a.; Rami; Maxwell., P. Analysing the world potash industry. *Resources Policy* **2014**, *41*.
(856) Rawashdeh, A.; Rami; Xavier-Oliveira, E.; Maxwell., P. The potash market and its future prospects. *Resources Policy* **2016**, *47*.
(857) Razanajatovo, M. R.; Gao, W. Y.; Song, Y. R.; Zhao, X.; Sun, Q. N.; Zhang, Q. R. Selective adsorption of phosphate in water using lanthanum-based nanomaterials: A critical review. *Chinese Chemical Letters* **2021**, *32* (9), 2637-2647. DOI: 10.1016/j.cclet.2021.01.046.
(858) Reddy, N. K.; Devika, M.; Gopal, E. S. R. Review on Tin (II) Sulfide (SnS) Material: Synthesis, Properties, and Applications. *Critical Reviews in Solid State and Materials Sciences* **2015**, *40* (6), 359-398. DOI: 10.1080/10408436.2015.1053601.
(859) Rehder, D. Structure and function of vanadium compounds in living organisms. *Biometals* **1992**, *5* (1), 3-12. DOI: 10.1007/BF01079691.
(860) Reid, M. S.; Hoy, K. S.; Schofield, J. R. M.; Uppal, J. S.; Lin, Y. W.; Lu, X. F.; Peng, H. Y.; Le, X. C. Arsenic speciation analysis: A review with an emphasis on chromatographic separations. *Trac-Trends in Analytical Chemistry* **2020**, *123,* 115770. DOI: 10.1016/j.trac.2019.115770.
(861) Reif-Acherman, S. Several motivations, improved procedures, and different contexts: The first liquefactions of helium around the world. *International Journal of Refrigeration-Revue Internationale Du Froid* **2009**, *32* (5), 738-762. DOI: 10.1016/j.ijrefrig.2009.02.019.

(862) Reimann, C.; Matschullat, J.; Birke, M.; Salminen, R. Antimony in the environment: Lessons from geochemical mapping. *Applied Geochemistry* **2010**, *25* (2), 175-198. DOI: 10.1016/j.apgeochem.2009.11.011.
(863) Reis, V. A. T.; Duarte, A. C. Analytical methodologies for arsenic speciation in macroalgae: A critical review. *Trac-Trends in Analytical Chemistry* **2018**, *102*, 170-184. DOI: 10.1016/j.trac.2018.02.003.
(864) Reisdörfer, G.; Bertuol, D.; Tanabe, E. Extraction of neodymium from hard disk drives using supercritical CO2 with organic acids solutions acting as cosolvents. *Journal of CO2 Utilization* **2020**, *35*, 277-287. DOI: 10.1016/j.jcou.2019.10.008
(865) Remucal, C. K.; Ginder-Vogel, M. A critical review of the reactivity of manganese oxides with organic contaminants. *Environ Sci Process Impacts* **2014**, *16* (6), 1247-1266. DOI: 10.1039/c3em00703k.
(866) Renfrew, A. K.; O'Neill, E. S.; Hambley, T. W.; New, E. J. Harnessing the properties of cobalt coordination complexes for biological application. *Coordination Chemistry Reviews* **2018**, *375*, 221-233. DOI: 10.1016/j.ccr.2017.11.027.
(867) Rengel, Z. Role of calcium in aluminium toxicity. *New Phytologist* **1992**, *121*, 499-513. DOI: 10.1111/j.1469-8137.1992.tb01120.x
(868) Rengel, Z.; Bose, J.; Chen, Q.; Tripathi, B. N. Magnesium alleviates plant toxicity of aluminium and heavy metals. *Crop & Pasture Science* **2015**, *66* (12), 1298-1307. DOI: 10.1071/Cp15284.
(869) Riaz, M.; Yan, L.; Wu, X.; Hussain, S.; Aziz, O.; Jiang, C. Mechanisms of organic acids and boron induced tolerance of aluminum toxicity: A review. *Ecotoxicol Environ Saf* **2018**, *165*, 25-35. DOI: 10.1016/j.ecoenv.2018.08.087.
(870) Ribera, D.; Labrot, F.; Tisnerat, G.; Narbonne, J. F. Uranium in the environment: occurrence, transfer, and biological effects. *Rev Environ Contam Toxicol* **1996**, *146*, 53-89. DOI: 10.1007/978-1-4613-8478-6_3.
(871) Richard, F. C.; Bourg, A. C. M. Aqueous Geochemistry of Chromium - a Review. *Water Research* **1991**, *25* (7), 807-816. DOI: Doi 10.1016/0043-1354(91)90160-R.
(872) Rim, K. Effects of rare earth elements on the environment and human health: a literature review. *Toxicology and Environmental Health Sciences* **2016**, *8*, 189-200. DOI: 10.1007/s13530-016-0276-y
(873) Rinklebe, J.; Shaheen, S. M. Redox chemistry of nickel in soils and sediments: A review. *Chemosphere* **2017**, *179*, 265-278. DOI: 10.1016/j.chemosphere.2017.02.153.
(874) Rinkovec, J. Platinum, palladium, and rhodium in airborne particulate matter. *Arh Hig Rada Toksikol* **2019**, *70* (4), 224-231. DOI: 10.2478/aiht-2019-70-3293.
(875) Ritchie, G. S. P. Role of Dissolution and Precipitation of Minerals in Controlling Soluble Aluminum in Acidic Soils. *Advances in Agronomy, Vol 53* **1994**, *53*, 47-83. DOI: 10.1016/S0065-2113(08)60612-4.
(876) Riva, S.; Yusenko, K. V.; Lavery, N. P.; Jarvis, D. J.; Brown, S. G. R. The scandium effect in multicomponent alloys. *International Materials Reviews* **2016**, *61* (3), 203-228. DOI: 10.1080/09506608.2015.1137692.
(877) Rodríguez, F.; Pérez, N.; Padrón, E.; Melián, G.; Hernández, P.; Asensio-Ramos, M.; Dionis, S.; López, G.; Marrero, R.; Padilla, G.; Barrancos, J.; Hidalgo, R. Diffuse helium and hydrogen degassing to reveal hidden geothermal resources in oceanic volcanic islands: The Canarian archipelago case study. *Surveys in Geophysics* **2015**, *36*, 351-369. DOI: 10.1007/s10712-015-9320-8

(878) Roggenbeck, B. A.; Banerjee, M.; Leslie, E. M. Cellular arsenic transport pathways in mammals. *J Environ Sci (China)* **2016**, *49*, 38-58. DOI: 10.1016/j.jes.2016.10.001.
(879) Rogosnitzky, M.; Branch, S. Gadolinium-based contrast agent toxicity: a review of known and proposed mechanisms. *Biometals* **2016**, *29* (3), 365-376. DOI: 10.1007/s10534-016-9931-7.
(880) Rogowska, J.; Olkowska, E.; Ratajczyk, W.; Wolska, L. Gadolinium as a new emerging contaminant of aquatic environments. *Environmental Toxicology and Chemistry* **2018**, *37* (6), 1523-1534. DOI: 10.1002/etc.4116.
(881) Rojas-Lemus, M.; Lopez-Valdez, N.; Bizarro-Nevares, P.; Gonzalez-Villalva, A.; Ustarroz-Cano, M.; Zepeda-Rodriguez, A.; Pasos-Najera, F.; Garcia-Pelaez, I.; Rivera-Fernandez, N.; Fortoul, T. I. Toxic Effects of Inhaled Vanadium Attached to Particulate Matter: A Literature Review. *Int J Environ Res Public Health* **2021**, *18* (16). DOI: 10.3390/ijerph18168457.
(882) Román-Vázquez; Mabiel. Recent advances on synthesis and applications of lead-and tin-free perovskites. *Journal of Alloys and Compounds* **2020**, *835*.
(883) Romero-Freire, M.; Santos-Echeandia, J.; Neira, P.; Cobelo-Garcia, A. Less-Studied Technology-Critical Elements (Nb, Ta, Ga, In, Ge, Te) in the Marine Environment: Review on Their Concentrations in Water and Organisms. *Frontiers in Marine Science* **2019**, *6,* 532. DOI: 10.3389/fmars.2019.00532.
(884) Rosas-Castor, J. M.; Guzman-Mar, J. L.; Hernandez-Ramirez, A.; Garza-Gonzalez, M. T.; Hinojosa-Reyes, L. Arsenic accumulation in maize crop (Zea mays): a review. *Sci Total Environ* **2014**, *488-489*, 176-187. DOI: 10.1016/j.scitotenv.2014.04.075.
(885) Rosenberg, E. Germanium: environmental occurrence, importance and speciation. *Reviews in Environmental Science and Bio/Technology* **2009**, *8*, 29-57. DOI: 10.1007/s11157-008-9143-x
(886) Rouschias, G. Recent advances in the chemistry of rhenium. *Chemical Reviews* **1974**, *74*, 531-566.
(887) Rout, G. R.; Das, P. Effect of Metal Toxicity on Plant Growth and Metabolism: I. Zinc. *Sustainable Agriculture* **2009**, 873-884. DOI: 10.1051/agro:2002073.
(888) Rouxel, O. J.; Luais, B. Germanium Isotope Geochemistry. *Non-Traditional Stable Isotopes* **2017**, *82*, 601-656. DOI: 10.2138/rmg.2017.82.14.
(889) Roy, A. K.; Sharma, A.; Talukder, G. Some Aspects of Aluminum Toxicity in Plants. *Botanical Review* **1988**, *54* (2), 145-178. DOI: 10.1007/Bf02858527.
(890) Roy, P.; Saha, A. Metabolism and toxicity of arsenic: A human carcinogen. *Current Science* **2002**, *82* (1), 38-45.
(891) Roy, S. Late Archean initiation of manganese metallogenesis: its significance and environmental controls. *Ore Geology Reviews* **2000**, *17* (3), 179-198. DOI: 10.1016/S0169-1368(00)00013-5.
(892) Roy, S. Sedimentary manganese metallogenesis in response to the evolution of the Earth system. *Earth-Science Reviews* **2006**, *77* (4), 273-305. DOI: 10.1016/j.earscirev.2006.03.004.
(893) Röyset, J.; Ryum, N. Scandium in aluminium alloys. *International Materials Reviews* **2005**, *50*, 19-44. DOI: 10.1179/174328005X14311
(894) Rufford, T. E.; Chan, K. I.; Huang, S. H.; May, E. F. A Review of Conventional and Emerging Process Technologies for the Recovery of Helium from Natural Gas. *Adsorption Science & Technology* **2014**, *32* (1), 49-72. DOI: 10.1260/0263-6174.32.1.49.
(895) Ruzicka, V. Vein Uranium Deposits. *Ore Geology Reviews* **1993**, *8* (3-4), 247-276. DOI: 10.1016/0169-1368(93)90019-U.

(896) Ryan, J. G. Trace-element systematics of beryllium in terrestrial materials. *Beryllium: Mineralogy, Petrology, and Geochemistry* **2002**, *50*, 121-145. DOI: 10.2138/rmg.2002.50.3.
(897) Rylander, R. Bioavailability of magnesium salts–a review. *J Pharm Nutr Sci* **2014**, *4*, 57-59.
(898) Rzigalinski, B. A.; Carfagna, C. S.; Ehrich, M. Cerium oxide nanoparticles in neuroprotection and considerations for efficacy and safety. *Wiley Interdiscip Rev Nanomed Nanobiotechnol* **2017**, *9* (4). DOI: 10.1002/wnan.1444.
(899) Saerens, A.; Ghosh, M.; Verdonck, J.; Godderis, L. Risk of Cancer for Workers Exposed to Antimony Compounds: A Systematic Review. *International Journal of Environmental Research and Public Health* **2019**, *16* (22), 4474. DOI: 10.3390/ijerph16224474.
(900) Saha, J. C.; Dikshit, A. K.; Bandyopadhyay, M.; Saha, K. C. A review of arsenic poisoning and its effects on human health. *Critical Reviews in Environmental Science and Technology* **1999**, *29* (3), 281-313. DOI: 10.1080/10643389991259227.
(901) Saifullah; Dahlawi, S.; Naeem, A.; Iqbal, M.; Farooq, M.; Bibi, S.; Rengel, Z. Opportunities and challenges in the use of mineral nutrition for minimizing arsenic toxicity and accumulation in rice: A critical review. *Chemosphere* **2018**, *194*, 171-188. DOI: 10.1016/j.chemosphere.2017.11.149.
(902) Samac, D. A.; Tesfaye, M. Plant improvement for tolerance to aluminum in acid soils - a review. *Plant Cell Tissue and Organ Culture* **2003**, *75* (3), 189-207. DOI: 10.1023/A:1025843829545.
(903) Samal, S.; Ray, A. K.; Bandopadhyay, A. Proposal for resources, utilization and processes of red mud in India - A review. *International Journal of Mineral Processing* **2013**, *118*, 43-55. DOI: 10.1016/j.minpro.2012.11.001.
(904) Samantaray, S.; Rout, G. R.; Das, P. Role of chromium on plant growth and metabolism. *Acta Physiologiae Plantarum* **1998**, *20* (2), 201-212. DOI: 10.1007/s11738-998-0015-3.
(905) Samaras, M. Multiscale Modelling: the role of helium in iron. *Materials Today* **2009**, *12* (11), 46-53. DOI: 10.1016/S1369-7021(09)70298-6.
(906) Samimi, M.; Zakeri, M.; Alobaid, F.; Aghel, B. A brief review of recent results in arsenic adsorption process from aquatic environments by metal-organic frameworks: Classification based on kinetics, isotherms and thermodynamics behaviors. *Nanomaterials* **2022**, *13*. DOI: 10.3390/nano13010060
(907) Sana, S. S.; Singh, R. P.; Sharma, M.; Srivastava, A. K.; Manchanda, G.; Rai, A. R.; Zhang, Z. J. Biogenesis and Application of Nickel Nanoparticles: A Review. *Curr Pharm Biotechnol* **2021**, *22* (6), 808-822. DOI: 10.2174/1389201022999210101235233.
(908) Santore, R. C.; Croteau, K.; Ryan, A. C.; Schlekat, C.; Middleton, E.; Garman, E.; Hoang, T. A Review of Water Quality Factors that Affect Nickel Bioavailability to Aquatic Organisms: Refinement of the Biotic Ligand Model for Nickel in Acute and Chronic Exposures. *Environmental Toxicology and Chemistry* **2021**, *40* (8), 2121-2134. DOI: 10.1002/etc.5109.
(909) Santore, R. C.; Mathew, R.; Paquin, P. R.; DiToro, D. Application of the biotic ligand model to predicting zinc toxicity to rainbow trout, fathead minnow, and Daphnia magna. *Comp Biochem Physiol C Toxicol Pharmacol* **2002**, *133* (1-2), 271-285. DOI: 10.1016/s1532-0456(02)00106-0.
(910) Santoro, L.; Putzolu, F.; Mondillo, N.; Boni, M.; Herrington, R. Trace element geochemistry of iron-(oxy)-hydroxides in Ni(Co)-laterites: Review, new data and implications for ore forming processes. *Ore Geology Reviews* **2022**, *140*. DOI: 10.1016/j.oregeorev.2021.104501

(911) Sapkota, P.; Kim, H. Zinc-air fuel cell, a potential candidate for alternative energy. *Journal of Industrial and Engineering Chemistry* **2009**, *15* (4), 445-450. DOI: 10.1016/j.jiec.2009.01.002.
(912) Sarkar, A.; Paul, B. The global menace of arsenic and its conventional remediation - A critical review. *Chemosphere* **2016**, *158*, 37-49. DOI: 10.1016/j.chemosphere.2016.05.043.
(913) Saunders, J. A.; Pivetz, B. E.; Voorhies, N.; Wilkin, R. T. Potential aquifer vulnerability in regions down-gradient from uranium in situ recovery (ISR) sites. *J Environ Manage* **2016**, *183*, 67-83. DOI: 10.1016/j.jenvman.2016.08.049.
(914) Savignan, L.; Faucher, S.; Chery, P.; Lespes, G. Platinum group elements contamination in soils: Review of the current state. *Chemosphere* **2021**, *271*, 129517. DOI: 10.1016/j.chemosphere.2020.129517.
(915) Savoca, D.; Pace, A. Bioaccumulation, Biodistribution, Toxicology and Biomonitoring of Organofluorine Compounds in Aquatic Organisms. *Int J Mol Sci* **2021**, *22* (12). DOI: 10.3390/ijms22126276.
(916) Sawicka, E.; Jurkowska, K.; Piwowar, A. Chromium (III) and chromium (VI) as important players in the induction of genotoxicity - current view. *Annals of Agricultural and Environmental Medicine* **2021**, *28* (1), 1-10. DOI: 10.26444/aaem/118228.
(917) Schaumloffel, D. Nickel species: analysis and toxic effects. *J Trace Elem Med Biol* **2012**, *26* (1), 1-6. DOI: 10.1016/j.jtemb.2012.01.002.
(918) Schijf, J.; Byrne, R. H. Speciation of yttrium and the rare earth elements in seawater: Review of a 20-year analytical journey. *Chemical Geology* **2021**, *584,* 120479. DOI: 10.1016/j.chemgeo.2021.120479.
(919) Schlesinger, W. H.; Klein, E. M.; Vengosh, A. Global biogeochemical cycle of vanadium. *Proc Natl Acad Sci U S A* **2017**, *114* (52), E11092-E11100. DOI: 10.1073/pnas.1715500114.
(920) Schlesinger, W. H.; Klein, E. M.; Vengosh, A. Global Biogeochemical Cycle of Fluorine. *Global Biogeochemical Cycles* **2020**, *34* (12), e2020GB006722. DOI: 10.1029/2020GB006722.
(921) Schmidt, S. B.; Husted, S. The Biochemical Properties of Manganese in Plants. *Plants (Basel)* **2019**, *8* (10). DOI: 10.3390/plants8100381.
(922) Scibior, A. Vanadium (V) and magnesium (Mg)-In vivo interactions: A review. *Chemicobiological interactions* **2016**, *258*.
(923) Scibior, A. Vanadium: Risks and possible benefits in the light of a comprehensive overview of its pharmacotoxicological mechanisms and multi-applications with a summary of further research trends. *Journal of Trace Elements in Medicine and Biology* **2020**, *61*.
(924) Sears, J. M.; Boyle, T. J. Structural properties of scandium inorganic salts. *Coordination Chemistry Reviews* **2017**, *340*, 154-171. DOI: 10.1016/j.ccr.2016.12.005.
(925) Séby, F.; Potin-Gautier, M.; Giffaut, E.; Donard, O. A critical review of thermodynamic data for inorganic tin species. *Geochimica et Cosmochimica Acta* **2001**, *65*, 3041-3053. DOI: 10.1016/S0016-7037(01)00645-7
(926) Sekar, N.; Li, J. P.; Shechter, Y. Vanadium salts as insulin substitutes: Mechanisms of action, a scientific and therapeutic tool in diabetes mellitus research. *Critical Reviews in Biochemistry and Molecular Biology* **1996**, *31* (5-6), 339-359. DOI: 10.3109/10409239609108721.
(927) Selvakumar, R.; Ramadoss, G.; Mridula, P. M.; Rajendran, K.; Thavamani, P.; Ravi, N.; Megharaj, M. Challenges and complexities in remediation of uranium contaminated soils: A review. *J Environ Radioact* **2018**, *192*, 592-603. DOI: 10.1016/j.jenvrad.2018.02.018.

(928) Selway, J.; Breaks, F.; Tindle, A. A review of rare-element (Li-Cs-Ta) pegmatite exploration techniques for the Superior Province, Canada, and large worldwide tantalum deposits. *Exploration and Mining Geology* **2005**, *14*, 1-30. DOI: 10.2113/gsemg.14.1-4.1
(929) Seo, Y. R.; Kim, B. J.; Ryu, J. C. Molecular and genomic approaches on nickel toxicity and carcinogenicity. *Molecular & Cellular Toxicology* **2005**, *1* (2), 73-77.
(930) Seredin, V. V.; Dai, S. F. Coal deposits as potential alternative sources for lanthanides and yttrium. *International Journal of Coal Geology* **2012**, *94*, 67-93. DOI: 10.1016/j.coal.2011.11.001.
(931) Shah, A.; Tanveer, M.; Hussain, S.; Yang, G. Beryllium in the environment: Whether fatal for plant growth?. *Reviews in Environmental Science and Bio/Technology* **2016**, *15*, 549-561. https://doi.org/10.1007/s11157-016-9412-z
(932) Shaheen, S. M.; Alessi, D. S.; Tack, F. M. G.; Ok, Y. S.; Kim, K. H.; Gustafsson, J. P.; Sparks, D. L.; Rinklebe, J. Redox chemistry of vanadium in soils and sediments: Interactions with colloidal materials, mobilization, speciation, and relevant environmental implications- A review. *Adv Colloid Interface Sci* **2019**, *265*, 1-13. DOI: 10.1016/j.cis.2019.01.002.
(933) Shahid, M.; Shamshad, S.; Rafiq, M.; Khalid, S.; Bibi, I.; Niazi, N. K.; Dumat, C.; Rashid, M. I. Chromium speciation, bioavailability, uptake, toxicity and detoxification in soil-plant system: A review. *Chemosphere* **2017**, *178*, 513-533. DOI: 10.1016/j.chemosphere.2017.03.074.
(934) Shahzad, B.; Mughal, M. N.; Tanveer, M.; Gupta, D.; Abbas, G. Is lithium biologically an important or toxic element to living organisms? An overview. *Environmental Science and Pollution Research* **2017**, *24* (1), 103-115. DOI: 10.1007/s11356-016-7898-0.
(935) Shahzad, B.; Tanveer, M.; Hassan, W.; Shah, A. N.; Anjum, S. A.; Cheema, S. A.; Ali, I. Lithium toxicity in plants: Reasons, mechanisms and remediation possibilities - A review. *Plant Physiology and Biochemistry* **2016**, *107*, 104-115. DOI: 10.1016/j.plaphy.2016.05.034.
(936) Shahzad, B.; Tanveer, M.; Rehman, A.; Cheema, S. A.; Fahad, S.; Rehman, S.; Sharma, A. Nickel; whether toxic or essential for plants and environment - A review. *Plant Physiol Biochem* **2018**, *132*, 641-651. DOI: 10.1016/j.plaphy.2018.10.014.
(937) Shakeel, M.; Jabeen, F.; Shabbir, S.; Asghar, M.; Khan, M.; Chaudhry, A. Toxicity of nano-titanium dioxide (TiO 2-NP) through various routes of exposure: a review. *Biological Trace Element Research* **2016**, *172*, 1-36. https://doi.org/10.1007/s12011-015-0550-x
(938) Shankar, S.; Shanker, U.; Shikha. Arsenic contamination of groundwater: a review of sources, prevalence, health risks, and strategies for mitigation. *ScientificWorldJournal* **2014**, *2014*, 304524. DOI: 10.1155/2014/304524.
(939) Shanker, A. K.; Cervantes, C.; Loza-Tavera, H.; Avudainayagam, S. Chromium toxicity in plants. *Environ Int* **2005**, *31* (5), 739-753. DOI: 10.1016/j.envint.2005.02.003.
(940) Shanker, A. K.; Djanaguiraman, M.; Venkateswarlu, B. Chromium interactions in plants: current status and future strategies. *Metallomics* **2009**, *1* (5), 375-383. DOI: 10.1039/b904571f.
(941) Shao, M. Palladium-based electrocatalysts for hydrogen oxidation and oxygen reduction reactions. *Journal of Power Sources* **2011**, *196* (5), 2433-2444. DOI: 10.1016/j.jpowsour.2010.10.093.
(942) Sharma, A.; Kapoor, D.; Wang, J.; Shahzad, B.; Kumar, V.; Bali, A. S.; Jasrotia, S.; Zheng, B.; Yuan, H.; Yan, D. Chromium Bioaccumulation and Its Impacts on Plants: An Overview. *Plants (Basel)* **2020**, *9* (1). DOI: 10.3390/plants9010100.
(943) Sharma, A. K.; Tjell, J. C.; Sloth, J. J.; Holm, P. E. Review of arsenic contamination, exposure through water and food and low cost mitigation options for rural areas. *Applied Geochemistry* **2014**, *41*, 11-33. DOI: 10.1016/j.apgeochem.2013.11.012.

(944) Sharma, R.; Garg, R.; Kumari, A. A Review on Biogenic Synthesis, Applications and Toxicity Aspects of Zinc Oxide Nanoparticles. *Excli Journal* **2020**, *19*, 1325-1340. DOI: 10.17179/excli2020-2842.
(945) Sharma, V. K. Aggregation and toxicity of titanium dioxide nanoparticles in aquatic environment--a review. *J Environ Sci Health A Tox Hazard Subst Environ Eng* **2009**, *44* (14), 1485-1495. DOI: 10.1080/10934520903263231.
(946) Shaw, D. The geochemistry of gallium, indium, thallium—a review. *Physics and Chemistry of the Earth* **1957**, *2*, 164-211. https://doi.org/10.1016/0079-1946(57)90009-5
(947) Shay, E.; De Gandiaga, E.; Madl, A. K. Considerations for the development of health-based surface dust cleanup criteria for beryllium. *Crit Rev Toxicol* **2013**, *43* (3), 220-243. DOI: 10.3109/10408444.2013.767308.
(948) Shelnutt, S. R.; Goad, P.; Belsito, D. V. Dermatological toxicity of hexavalent chromium. *Crit Rev Toxicol* **2007**, *37* (5), 375-387. DOI: 10.1080/10408440701266582.
(949) Shemi, A.; Magumise, A.; Ndlovu, S.; Sacks, N. Recycling of tungsten carbide scrap metal: A review of recycling methods and future prospects. *Minerals Engineering* **2018**, *122*, 195-205. DOI: 10.1016/j.mineng.2018.03.036.
(950) Shen, L. T.; Li, X. B.; Lindberg, D.; Taskinen, P. Tungsten extractive metallurgy: A review of processes and their challenges for sustainability. *Minerals Engineering* **2019**, *142,* 105934. DOI: 10.1016/j.mineng.2019.105934.
(951) Shen, L. T.; Tesfaye, F.; Li, X. B.; Lindberg, D.; Taskinen, P. Review of rhenium extraction and recycling technologies from primary and secondary resources. *Minerals Engineering* **2021**, *161,* 106719. DOI: 10.1016/j.mineng.2020.106719.
(952) Shetty, R.; Vidya, C. S.; Prakash, N. B.; Lux, A.; Vaculik, M. Aluminum toxicity in plants and its possible mitigation in acid soils by biochar: A review. *Sci Total Environ* **2021**, *765*, 142744. DOI: 10.1016/j.scitotenv.2020.142744.
(953) Shi, H.; Magaye, R.; Castranova, V.; Zhao, J. Titanium dioxide nanoparticles: a review of current toxicological data. *Part Fibre Toxicol* **2013**, *10*, 15. DOI: 10.1186/1743-8977-10-15.
(954) Shi, J. Y.; Shi, B. Environment-Friendly Design of Lithium Batteries Starting from Biopolymer-Based Electrolyte. *Nano* **2021**, *16* (05), 2130006. DOI: 10.1142/S1793292021300061.
(955) Shi, W.; Gao, M.; Wei, J.; Gao, J.; Fan, C.; Ashalley, E.; Li, H.; Wang, Z. Tin Selenide (SnSe): Growth, Properties, and Applications. *Adv Sci (Weinh)* **2018**, *5* (4), 1700602. DOI: 10.1002/advs.201700602.
(956) Shikika, A.; Sethurajan, M.; Muvundja, F.; Mugumaoderha, M. C.; Gaydardzhiev, S. A review on extractive metallurgy of tantalum and niobium. *Hydrometallurgy* **2020**, *198,* 105496. DOI: 10.1016/j.hydromet.2020.105496.
(957) Shin, S. H.; Kim, H. O.; Rim, K. T. Worker Safety in the Rare Earth Elements Recycling Process From the Review of Toxicity and Issues. *Saf Health Work* **2019**, *10* (4), 409-419. DOI: 10.1016/j.shaw.2019.08.005.
(958) Shirale, A. O.; Meena, B. P.; Gurav, P. P.; Srivastava, S.; Biswas, A. K.; Thakur, J. K.; Somasundaram, J.; Patra, A. K.; Rao, A. S. Prospects and challenges in utilization of indigenous rocks and minerals as source of potassium in farming rocks and minerals as source of potassium. *Journal of Plant Nutrition* **2019**, *42* (19), 2682-2701. DOI: 10.1080/01904167.2019.1659353.
(959) Shotyk, W.; Krachler, M.; Chen, B. Antimony: global environmental contaminant. *J Environ Monit* **2005**, *7* (12), 1135-1136. DOI: 10.1039/b515468p.

(960) Shotyk, W.; Krachler, M.; Chen, B. Anthropogenic impacts on the biogeochemistry and cycling of antimony. *Met Ions Biol Syst* **2005**, *44*, 171-203.
(961) Shrestha, R.; Sorg, J. A. Terbium chloride influences Clostridium difficile spore germination. *Anaerobe* **2019**, *58*, 80-88. DOI: 10.1016/j.anaerobe.2019.03.016.
(962) Siddiqi, K. S.; Rahman, A. U.; Tajuddin; Husen, A. Properties of Zinc Oxide Nanoparticles and Their Activity Against Microbes. *Nanoscale Research Letters* **2018**, *13,* 141. DOI: 10.1186/s11671-018-2532-3.
(963) Siebel, A. M.; Vianna, M. R.; Bonan, C. D. Pharmacological and toxicological effects of lithium in zebrafish. *ACS Chem Neurosci* **2014**, *5* (6), 468-476. DOI: 10.1021/cn500046h.
(964) Silva, d.; Lallo, B. Relationship between structure and antimicrobial activity of zinc oxide nanoparticles: An overview. *International journal of nanomedicine* **2019**, *14*.
(965) Silveira, A. V.; Fuchs, M. S.; Pinheiro, D. K.; Tanabe, E. H.; Bertuol, D. A. Recovery of indium from LCD screens of discarded cell phones. *Waste Manag* **2015**, *45*, 334-342. DOI: 10.1016/j.wasman.2015.04.007.
(966) Simoes, C. C.; Melo, J. O.; Magalhaes, J. V.; Guimaraes, C. T. Genetic and molecular mechanisms of aluminum tolerance in plants. *Genet Mol Res* **2012**, *11* (3), 1949-1957. DOI: 10.4238/2012.July.19.14.
(967) Simon, A. C.; Pettke, T. Platinum solubility and partitioning in a felsic melt-vapor-brine assemblage. *Geochimica Et Cosmochimica Acta* **2009**, *73* (2), 438-454. DOI: 10.1016/j.gca.2008.10.020.
(968) Simonsen, L. O.; Harbak, H.; Bennekou, P. Cobalt metabolism and toxicology--a brief update. *Sci Total Environ* **2012**, *432*, 210-215. DOI: 10.1016/j.scitotenv.2012.06.009.
(969) Singh; Veerendra; Chakraborty, T.; Tripathy., S. K. A Review of low grade manganese ore upgradation processes. *Mineral Processing and Extractive Metallurgy Review* **2020**, *41*.
(970) Singh, G.; Kumari, B.; Sinam, G.; Kriti; Kumar, N.; Mallick, S. Fluoride distribution and contamination in the water, soil and plants continuum and its remedial technologies, an Indian perspective- a review. *Environ Pollut* **2018**, *239*, 95-108. DOI: 10.1016/j.envpol.2018.04.002.
(971) Singh, H. P.; Mahajan, P.; Kaur, S.; Batish, D. R.; Kohli, R. K. Chromium toxicity and tolerance in plants. *Environmental Chemistry Letters* **2013**, *11* (3), 229-254. DOI: 10.1007/s10311-013-0407-5.
(972) Singh, N.; Kumar, D.; Sahu, A. P. Arsenic in the environment: effects on human health and possible prevention. *J Environ Biol* **2007**, *28* (2 Suppl), 359-365.
(973) Singh, R.; Singh, S.; Parihar, P.; Singh, V. P.; Prasad, S. M. Arsenic contamination, consequences and remediation techniques: a review. *Ecotoxicol Environ Saf* **2015**, *112*, 247-270. DOI: 10.1016/j.ecoenv.2014.10.009.
(974) Singh, S. Zinc oxide nanoparticles impacts: cytotoxicity, genotoxicity, developmental toxicity, and neurotoxicity. *Toxicol Mech Methods* **2019**, *29* (4), 300-311. DOI: 10.1080/15376516.2018.1553221.
(975) Sinha, R.; Kumar, R.; Sharma, P.; Kant, N.; Shang, J.; Aminabhavi, T. M. Removal of hexavalent chromium via biochar-based adsorbents: State-of-the-art, challenges, and future perspectives. *J Environ Manage* **2022**, *317*, 115356. DOI: 10.1016/j.jenvman.2022.115356.
(976) Sinha, V.; Pakshirajan, K.; Chaturvedi, R. Chromium tolerance, bioaccumulation and localization in plants: An overview. *J Environ Manage* **2018**, *206*, 715-730. DOI: 10.1016/j.jenvman.2017.10.033.

(977) Sirelkhatim, A.; Mahmud, S.; Seeni, A.; Kaus, N. H. M.; Ann, L. C.; Bakhori, S. K. M.; Hasan, H.; Mohamad, D. Review on Zinc Oxide Nanoparticles: Antibacterial Activity and Toxicity Mechanism. *Nanomicro Lett* **2015**, *7* (3), 219-242. DOI: 10.1007/s40820-015-0040-x.
(978) Smedley, P. L.; Kinniburgh, D. G. A review of the source, behaviour and distribution of arsenic in natural waters. *Applied Geochemistry* **2002**, *17* (5), 517-568. DOI: 10.1016/S0883-2927(02)00018-5.
(979) Smith, K.; Huyck, H. An overview of the abundance, relative mobility, bioavailability, and human toxicity of metals. *The environmental geochemistry of mineral deposits* **1999**, *6*, 29-70. https://doi.org/10.5382/Rev.06.02
(980) Smith, S. E.; Christophersen, H. M.; Pope, S.; Smith, F. A. Arsenic uptake and toxicity in plants: integrating mycorrhizal influences. *Plant and Soil* **2010**, *327* (1-2), 1-21. DOI: 10.1007/s11104-009-0089-8.
(981) Sobolev, O. I.; Gutyj, B. V.; Sobolieva, S. V.; Borshch, O. O.; Kushnir, I. M.; Petryshak, R. A.; Naumyuk, O. S.; Kushnir, V. I.; Petryshak, O. Y.; Zhelavskyi, M. M.; Todoriuk, V. B.; Sus, H. V.; Levkivska, N. D.; Vysotskij, A. O.; Magrelo, N. V. A Review of germanium environmental distribution, migration and accumulation. *Ukrainian Journal of Ecology* **2020**, *10* (2), 200-208. DOI: 10.15421/2020_86.
(982) Sodhi, K. K.; Kumar, M.; Agrawal, P. K.; Singh, D. K. Perspectives on arsenic toxicity, carcinogenicity and its systemic remediation strategies. *Environmental Technology & Innovation* **2019**, *16,* 100462. DOI: 10.1016/j.eti.2019.100462.
(983) Song, B.; Zhou, T.; Yang, W.; Liu, J.; Shao, L. Contribution of oxidative stress to TiO(2) nanoparticle-induced toxicity. *Environ Toxicol Pharmacol* **2016**, *48*, 130-140. DOI: 10.1016/j.etap.2016.10.013.
(984) Song, J. F.; Pan, F. S.; Jiang, B.; Atrens, A.; Zhang, M. X.; Lu, Y. A review on hot tearing of magnesium alloys. *Journal of Magnesium and Alloys* **2016**, *4* (3), 151-172. DOI: 10.1016/j.jma.2016.08.003.
(985) Song, J. F.; She, J.; Chen, D. L.; Pan, F. S. Latest research advances on magnesium and magnesium alloys worldwide. *Journal of Magnesium and Alloys* **2020**, *8* (1), 1-41. DOI: 10.1016/j.jma.2020.02.003.
(986) Song, P. P.; Yang, Z. H.; Zeng, G. M.; Yang, X.; Xu, H. Y.; Wang, L. K.; Xu, R.; Xiong, W. P.; Ahmad, K. Electrocoagulation treatment of arsenic in wastewaters: A comprehensive review. *Chemical Engineering Journal* **2017**, *317*, 707-725. DOI: 10.1016/j.cej.2017.02.086.
(987) Song, X.; Fiati Kenston, S. S.; Kong, L.; Zhao, J. Molecular mechanisms of nickel induced neurotoxicity and chemoprevention. *Toxicology* **2017**, *392*, 47-54. DOI: 10.1016/j.tox.2017.10.006.
(988) Souidi, M.; Tissandie, E.; Racine, R.; Ben Soussan, H.; Rouas, C.; Grignard, E.; Dublineau, I.; Gourmelon, P.; Lestaevel, P.; Gueguen, Y. Uranium: properties and biological effects after internal contamination. *Ann Biol Clin (Paris)* **2009**, *67* (1), 23-38. DOI: 10.1684/abc.2008.0290.
(989) Souza, J. M. O.; Carneiro, M. F. H.; Paulelli, A. C. C.; Grotto, D.; Magalhaes, A. M.; Barbosa, F.; Batista, B. L. Arsenic and Rice: Toxicity, Metabolism, and Food Safety. *Quimica Nova* **2015**, *38* (1), 118-127. DOI: 10.5935/0100-4042.20140279.
(990) Sowlat, M. H.; Kakavandi, B.; Lotfi, S.; Yunesian, M.; Abdollahi, M.; Kalantary, R. R. A systematic review on the efficiency of cerium-impregnated activated carbons for the removal of gas-phase, elemental mercury from flue gas. *Environmental Science and Pollution Research* **2017**, *24* (13), 12092-12103. DOI: 10.1007/s11356-017-8559-7.

(991) Sparks, D. Potassium dynamics in soils. *Advances in soil science* Springer, New York, NY, **1987**, 1-63.
(992) Spencer, N.; Roe, A. Physical properties and phase identification in yttrium—alkaline earth—bismuth—copper oxide systems. **1988**, 145-154. http://doi.org/10.1021/bk-1988-0377.ch012
(993) Sposito, G. *The environmental chemistry of aluminum*; CRC Press, **2020**.
(994) Spry, D. J.; Wiener, J. G. Metal bioavailability and toxicity to fish in low-alkalinity lakes: A critical review. *Environ Pollut* **1991**, *71* (2-4), 243-304. DOI: 10.1016/0269-7491(91)90034-t.
(995) Sreekanth, T. V. M.; Nagajyothi, P. C.; Lee, K. D.; Prasad, T. N. V. K. V. Occurrence, physiological responses and toxicity of nickel in plants. *International Journal of Environmental Science and Technology* **2013**, *10* (5), 1129-1140. DOI: 10.1007/s13762-013-0245-9.
(996) Srivastava, R. R.; Lee, J. C.; Kim, M. S. Complexation chemistry in liquid-liquid extraction of rhenium. *Journal of Chemical Technology and Biotechnology* **2015**, *90* (10), 1752-1764. DOI: 10.1002/jctb.4707.
(997) Srivastava, S.; Flora, S. J. S. Fluoride in Drinking Water and Skeletal Fluorosis: a Review of the Global Impact. *Curr Environ Health Rep* **2020**, *7* (2), 140-146. DOI: 10.1007/s40572-020-00270-9.
(998) Sruthi, S.; Ashtami, J.; Mohanan, P. V. Biomedical application and hidden toxicity of Zinc oxide nanoparticles. *Materials Today Chemistry* **2018**, *10*, 175-186. DOI: 10.1016/j.mtchem.2018.09.008.
(999) Stambulska, U. Y.; Bayliak, M. M.; Lushchak, V. I. Chromium(VI) Toxicity in Legume Plants: Modulation Effects of Rhizobial Symbiosis. *Biomed Res Int* **2018**, *2018*, 8031213. DOI: 10.1155/2018/8031213.
(1000) Stefanidou, M.; Maravelias, C.; Dona, A.; Spiliopoulou, C. Zinc: a multipurpose trace element. *Arch Toxicol* **2006**, *80* (1), 1-9. DOI: 10.1007/s00204-005-0009-5.
(1001) Stojilovic, N.; Bender, E. T.; Ramsier, R. D. Surface chemistry of zirconium. *Progress in Surface Science* **2005**, *78* (3-4), 101-184. DOI: 10.1016/j.progsurf.2005.07.001.
(1002) Stollenwerk, K. Geochemical processes controlling transport of arsenic in groundwater: A review of adsorption. *Arsenic in ground water* **2003**, 67-100. DOI: 10.1007/0-306-47956-7_3
(1003) Strachel, R.; Zaborowska, M.; Wyszkowska, J. Deliberations on Zinc - a Trace Mineral or a Toxic Element? *Journal of Elementology* **2016**, *21* (2), 625-639. DOI: 10.5601/jelem.2015.20.3.991.
(1004) Strigul, N. Does speciation matter for tungsten ecotoxicology?. *Ecotoxicology and Environmental Safety* **2010**, *73*, 1099-1113. https://doi.org/10.1016/j.ecoenv.2010.05.005
(1005) Su, Z. J.; Zhang, Y. B.; Liu, B. B.; Lu, M. M.; Li, G. H.; Jiang, T. Extraction and Separation of Tin from Tin-Bearing Secondary Resources: A Review. *Jom* **2017**, *69* (11), 2364-2372. DOI: 10.1007/s11837-017-2509-1.
(1006) Sule, K.; Umbsaar, J.; Prenner, E. J. Mechanisms of Co, Ni, and Mn toxicity: From exposure and homeostasis to their interactions with and impact on lipids and biomembranes. *Biochimica Et Biophysica Acta-Biomembranes* **2020**, *1862* (8), 183250. DOI: 10.1016/j.bbamem.2020.183250.
(1007) Sultana, M. Y.; Akratos, C. S.; Pavlou, S.; Vayenas, D. V. Chromium removal in constructed wetlands: A review. *International Biodeterioration & Biodegradation* **2014**, *96*, 181-190. DOI: 10.1016/j.ibiod.2014.08.009.

(1008) Sun, H. J.; Rathinasabapathi, B.; Wu, B.; Luo, J.; Pu, L. P.; Ma, L. Q. Arsenic and selenium toxicity and their interactive effects in humans. *Environment International* **2014**, *69*, 148-158. DOI: 10.1016/j.envint.2014.04.019.
(1009) Sun, S. J.; Liao, R. Q.; Cong, Y. N.; Sui, Q. L.; Li, A. Geochemistry and mineralization of titanium. *Acta Petrologica Sinica* **2020**, *36* (1), 68-76. DOI: 10.18654/1000-0569/2020.01.08.
(1010) Sun, Y.; Liu, G.; Cai, Y. Thiolated arsenicals in arsenic metabolism: Occurrence, formation, and biological implications. *J Environ Sci (China)* **2016**, *49*, 59-73. DOI: 10.1016/j.jes.2016.08.016.
(1011) Sundar, S.; Chakravarty, J. Antimony toxicity. *Int J Environ Res Public Health* **2010**, *7* (12), 4267-4277. DOI: 10.3390/ijerph7124267.
(1012) Suriyagoda, L. D. B.; Dittert, K.; Lambers, H. Mechanism of arsenic uptake, translocation and plant resistance to accumulate arsenic in rice grains. *Agriculture Ecosystems & Environment* **2018**, *253*, 23-37. DOI: 10.1016/j.agee.2017.10.017.
(1013) Suzuki, Y.; Banfield, J. Geomicrobiology of Uranium. *Reviews in Mineralogy* **1999**, 393-432. DOI: 10.1515/9781501509193-013
(1014) Swaddle, T. W. Silicate complexes of aluminum(III) in aqueous systems. *Coordination Chemistry Reviews* **2001**, *219*, 665-686. DOI: 10.1016/S0010-8545(01)00362-9.
(1015) Swain, B. Recovery and recycling of lithium: A review. *Separation and Purification Technology* **2017**, *172*, 388-403. DOI: 10.1016/j.seppur.2016.08.031.
(1016) Swiatkowska, I.; Martin, N.; Hart, A. J. Blood titanium level as a biomarker of orthopaedic implant wear. *Journal of Trace Elements in Medicine and Biology* **2019**, *53*, 120-128. DOI: 10.1016/j.jtemb.2019.02.013.
(1017) Szeto, W.; Leung, M.; Leung, D. Recent developments of titanium dioxide materials for aquatic antifouling application. *Journal of Marine Science and Technology* **2021**, *26*, 301-321. DOI: 10.1007/s00773-020-00720-x
(1018) Szklarska, D.; Rzymski., P. Is lithium a micronutrient? From biological activity and epidemiological observation to food fortification. *Biological trace element research* **2019**, *189*, 18-27.
(1019) Tabelin, C. B.; Dallas, J.; Casanova, S.; Pelech, T.; Bournival, G.; Saydam, S.; Canbulat, I. Towards a low-carbon society: A review of lithium resource availability, challenges and innovations in mining, extraction and recycling, and future perspectives. *Minerals Engineering* **2021**, *163,* 106743. DOI: 10.1016/j.mineng.2020.106743.
(1020) Tachikawa, K.; Piotrowski, A. M.; Bayon, G. Neodymium associated with foraminiferal carbonate as a recorder of seawater isotopic signatures. *Quaternary Science Reviews* **2014**, *88*, 1-13. DOI: 10.1016/j.quascirev.2013.12.027.
(1021) Tadesse, B.; Makuei, F.; Albijanic, B.; Dyer, L. The beneficiation of lithium minerals from hard rock ores: A review. *Minerals Engineering* **2019**, *131*, 170-184. DOI: 10.1016/j.mineng.2018.11.023.
(1022) Takeda, O.; Okabe, T. H. Current Status of Titanium Recycling and Related Technologies. *Jom* **2019**, *71* (6), 1981-1990. DOI: 10.1007/s11837-018-3278-1.
(1023) Tamás, J. M. Cellular and molecular mechanisms of antimony transport, toxicity and resistance. *Environmental Chemistry* **2016**, *13(6),* 955-962.
(1024) Tang, D. M.; Qin, K. Z.; Liu, B. G.; Sun, H.; Li, J. X. The major types, metallogenesis of platinum-group element deposits and some prospects. *Acta Petrologica Sinica* **2008**, *24* (3), 569-588.

(1025) Tang, S. Y.; Tabor, C.; Kalantar-Zadeh, K.; Dickey, M. D. Gallium Liquid Metal: The Devil's Elixir. *Annual Review of Materials Research, Vol 51, 2021* **2021**, *51*, 381-408. DOI: 10.1146/annurev-matsci-080819-125403.
(1026) Tang, X.; Huang, Y.; Li, Y.; Wang, L.; Pei, X.; Zhou, D.; He, P.; Hughes, S. S. Study on detoxification and removal mechanisms of hexavalent chromium by microorganisms. *Ecotoxicol Environ Saf* **2021**, *208*, 111699. DOI: 10.1016/j.ecoenv.2020.111699.
(1027) Tanner, P. A. Some misconceptions concerning the electronic spectra of tri-positive europium and cerium. *Chemical Society Reviews* **2013**, *42* (12), 5090-5101. DOI: 10.1039/c3cs60033e.
(1028) Tanveer, M.; Wang, L. Potential targets to reduce beryllium toxicity in plants: A review. *Plant Physiol Biochem* **2019**, *139*, 691-696. DOI: 10.1016/j.plaphy.2019.04.022.
(1029) Tao, J.; Tao, Z.; Liu, Z. H. Review on resources and recycling of germanium, with special focus on characteristics, mechanism and challenges of solvent extraction. *J Clean Prod* **2021**, *294,* 126217. DOI: 10.1016/j.jclepro.2021.126217.
(1030) Tarale, P.; Chakrabarti, T.; Sivanesan, S.; Naoghare, P.; Bafana, A.; Krishnamurthi, K. Potential Role of Epigenetic Mechanism in Manganese Induced Neurotoxicity. *Biomed Res Int* **2016**, *2016*, 2548792. DOI: 10.1155/2016/2548792.
(1031) Tatarchuk, T.; Shyichuk, A.; Mironyuk, I.; Naushad, M. A review on removal of uranium(VI) ions using titanium dioxide based sorbents. *Journal of Molecular Liquids* **2019**, *293,* 111563. DOI: 10.1016/j.molliq.2019.111563.
(1032) Taube, F. Manganese in occupational arc welding fumes--aspects on physiochemical properties, with focus on solubility. *Ann Occup Hyg* **2013**, *57* (1), 6-25. DOI: 10.1093/annhyg/mes053.
(1033) Taylor, T. P.; Ding, M.; Ehler, D. S.; Foreman, T. M.; Kaszuba, J. P.; Sauer, N. N. Beryllium in the environment: a review. *J Environ Sci Health A Tox Hazard Subst Environ Eng* **2003**, *38* (2), 439-469. DOI: 10.1081/ese-120016906.
(1034) Tchounwou, P. B.; Yedjou, C. G.; Udensi, U. K.; Pacurari, M.; Stevens, J. J.; Patlolla, A. K.; Noubissi, F.; Kumar, S. State of the science review of the health effects of inorganic arsenic: Perspectives for future research. *Environ Toxicol* **2019**, *34* (2), 188-202. DOI: 10.1002/tox.22673.
(1035) Tebo, B. M.; Bargar, J. R.; Clement, B. G.; Dick, G. J.; Murray, K. J.; Parker, D.; Verity, R.; Webb, S. M. Biogenic manganese oxides: Properties and mechanisms of formation. *Annual Review of Earth and Planetary Sciences* **2004**, *32*, 287-328. DOI: 10.1146/annurev.earth.32.101802.120213.
(1036) Tebo, B. M.; Johnson, H. A.; McCarthy, J. K.; Templeton, A. S. Geomicrobiology of manganese(II) oxidation. *Trends Microbiol* **2005**, *13* (9), 421-428. DOI: 10.1016/j.tim.2005.07.009.
(1037) Teng, F. Z. Magnesium Isotope Geochemistry. *Non-Traditional Stable Isotopes* **2017**, *82*, 219-287. DOI: 10.2138/rmg.2017.82.7.
(1038) Teng, F. Z.; Hu, Y.; Chauvel, C. Magnesium isotope geochemistry in arc volcanism. *Proc Natl Acad Sci U S A* **2016**, *113* (26), 7082-7087. DOI: 10.1073/pnas.1518456113.
(1039) Thangavel, P.; Viswanath, B.; Kim, S. Recent developments in the nanostructured materials functionalized with ruthenium complexes for targeted drug delivery to tumors. *Int J Nanomedicine* **2017**, *12*, 2749-2758. DOI: 10.2147/IJN.S131304.
(1040) Thomas, D. J.; Bradham, K. Role of complex organic arsenicals in food in aggregate exposure to arsenic. *J Environ Sci (China)* **2016**, *49*, 86-96. DOI: 10.1016/j.jes.2016.06.005.

(1041) Thomas, V. G. Elemental tungsten, tungsten-nickel alloys and shotgun ammunition: resolving issues of their relative toxicity. *European Journal of Wildlife Research* **2016**, *62* (1), 1-9. DOI: 10.1007/s10344-015-0979-4.
(1042) Tickner, B. J.; Stasiuk, G. J.; Duckett, S. B.; Angelovski, G. The use of yttrium in medical imaging and therapy: historical background and future perspectives. *Chem Soc Rev* **2020**, *49* (17), 6169-6185. DOI: 10.1039/c9cs00840c.
(1043) Tiruvayipati, S.; Bhassu, S. Host, pathogen and the environment: the case of Macrobrachium rosenbergii, Vibrio parahaemolyticus and magnesium. *Gut Pathog* **2016**, *8*, 15. DOI: 10.1186/s13099-016-0097-1.
(1044) Tkaczyk, A. H.; Bartl, A.; Amato, A.; Lapkovskis, V.; Petranikova, M. Sustainability evaluation of essential critical raw materials: cobalt, niobium, tungsten and rare earth elements. *Journal of Physics D-Applied Physics* **2018**, *51* (20), 203001. DOI: 10.1088/1361-6463/aaba99.
(1045) Toghill, K. E.; Lu, M.; Compton, R. G. Electroanalytical Determination of Antimony. *International Journal of Electrochemical Science* **2011**, *6* (8), 3057-3076.
(1046) Tomascak, P. B. Developments in the understanding and application of lithium isotopes in the earth and planetary sciences. *Geochemistry of Non-Traditional Stable Isotopes* **2004**, *55*, 153-195. DOI: 10.2138/gsrmg.55.1.153.
(1047) Toshniwal, A.; Kheraj, V. Development of organic-inorganic tin halide perovskites: A review. *Solar Energy* **2017**, *149*, 54-59. DOI: 10.1016/j.solener.2017.03.077.
(1048) Town, R. M.; Filella, M. Crucial role of the detection window in metal ion speciation analysis in aquatic systems: the interplay of thermodynamic and kinetic factors as exemplified by nickel and cobalt. *Analytica Chimica Acta* **2002**, *466* (2), 285-293. DOI: 10.1016/S0003-2670(02)00570-6.
(1049) Tricot, R. The Metallurgy and Functional-Properties of Hafnium. *Journal of Nuclear Materials* **1992**, *189* (3), 277-288. DOI: 10.1016/0022-3115(92)90382-U.
(1050) Tripathi, D.; Mani, V.; Pal, R. P. Vanadium in Biosphere and Its Role in Biological Processes. *Biol Trace Elem Res* **2018**, *186* (1), 52-67. DOI: 10.1007/s12011-018-1289-y.
(1051) Tsai, W. T. The decomposition products of sulfur hexafluoride (SF6): Reviews of environmental and health risk analysis. *Journal of Fluorine Chemistry* **2007**, *128* (11), 1345-1352. DOI: 10.1016/j.jfluchem.2007.06.008.
(1052) Tsangaris, J. M.; Williams, D. R. Tin in Pharmacy and Nutrition. *Applied Organometallic Chemistry* **1992**, *6* (1), 3-18. DOI: DOI 10.1002/aoc.590060103.
(1053) Tschan, M.; Robinson, B. H.; Schulin, R. Antimony in the soil-plant system - a review. *Environmental Chemistry* **2009**, *6* (2), 106-115. DOI: 10.1071/En08111.
(1054) Tucker, M. O.; Mclachlan, N. Fracture and Microstructure of Graphites. *Journal of Physics D-Applied Physics* **1993**, *26* (6), 893-907. DOI: 10.1088/0022-3727/26/6/001.
(1055) Turner, R. J.; Borghese, R.; Zannoni, D. Microbial processing of tellurium as a tool in biotechnology. *Biotechnology Advances* **2012**, *30* (5), 954-963. DOI: 10.1016/j.biotechadv.2011.08.018.
(1056) Tyler, G. Rare earth elements in soil and plant systems - A review. *Plant and Soil* **2004**, *267* (1-2), 191-206. DOI: 10.1007/s11104-005-4888-2.
(1057) Uddin, M. J.; Jeong, Y. K. Review: Efficiently performing periodic elements with modern adsorption technologies for arsenic removal. *Environ Sci Pollut Res Int* **2020**, *27* (32), 39888-39912. DOI: 10.1007/s11356-020-10323-z.
(1058) Ugwu, E. I.; Agunwamba, J. C. A review on the applicability of activated carbon derived from plant biomass in adsorption of chromium, copper, and zinc from industrial wastewater.

Environmental Monitoring and Assessment **2020**, *192* (4), 240. DOI: 10.1007/s10661-020-8162-0.
(1059) Ukhurebor, K. E.; Aigbe, U. O.; Onyancha, R. B.; Nwankwo, W.; Osibote, O. A.; Paumo, H. K.; Ama, O. M.; Adetunji, C. O.; Siloko, I. U. Effect of hexavalent chromium on the environment and removal techniques: A review. *J Environ Manage* **2021**, *280*, 111809. DOI: 10.1016/j.jenvman.2020.111809.
(1060) Ungureanu, G.; Santos, S.; Boaventura, R.; Botelho, C. Arsenic and antimony in water and wastewater: overview of removal techniques with special reference to latest advances in adsorption. *J Environ Manage* **2015**, *151*, 326-342. DOI: 10.1016/j.jenvman.2014.12.051.
(1061) Unruh, C.; Van Bavel, N.; Anikovskiy, M.; Prenner, E. J. Benefits and Detriments of Gadolinium from Medical Advances to Health and Ecological Risks. *Molecules* **2020**, *25* (23). DOI: 10.3390/molecules25235762.
(1062) Upadhyay, M. K.; Shukla, A.; Yadav, P.; Srivastava, S. A review of arsenic in crops, vegetables, animals and food products. *Food Chemistry* **2019**, *276*, 608-618. DOI: 10.1016/j.foodchem.2018.10.069.
(1063) Upadhyay, M. K.; Yadav, P.; Shukla, A.; Srivastava, S. Utilizing the Potential of Microorganisms for Managing Arsenic Contamination: A Feasible and Sustainable Approach. *Frontiers in Environmental Science* **2018**, *6,* 24. DOI: 10.3389/fenvs.2018.00024.
(1064) Ushakov, S. V.; Navrotsky, A.; Hong, Q. J.; van de Walle, A. Carbides and Nitrides of Zirconium and Hafnium. *Materials (Basel)* **2019**, *12* (17). DOI: 10.3390/ma12172728.
(1065) Usman, M.; Humayun, M.; Garba, M. D.; Ullah, L.; Zeb, Z.; Helal, A.; Suliman, M. H.; Alfaifi, B. Y.; Iqbal, N.; Abdinejad, M.; Tahir, A. A.; Ullah, H. Electrochemical Reduction of CO_2: A Review of Cobalt Based Catalysts for Carbon Dioxide Conversion to Fuels. *Nanomaterials (Basel)* **2021**, *11* (8). DOI: 10.3390/nano11082029.
(1066) Vaiopoulou, E.; Gikas, P. Effects of chromium on activated sludge and on the performance of wastewater treatment plants: A review. *Water Res* **2012**, *46* (3), 549-570. DOI: 10.1016/j.watres.2011.11.024.
(1067) Vakili, M.; Rafatullah, M.; Yuan, J.; Zwain, H. M.; Mojiri, A.; Gholami, Z.; Gholami, F.; Wang, W.; Giwa, A. S.; Yu, Y.; Cagnetta, G.; Yu, G. Nickel ion removal from aqueous solutions through the adsorption process: A review. *Reviews in Chemical Engineering* **2020**, *37(6)*, 755-778.
(1068) Vasseghian, Y.; Rad, S. S.; Vilas-Boas, J. A.; Khataee, A. A global systematic review, meta-analysis, and risk assessment of the concentration of vanadium in drinking water resources. *Chemosphere* **2021**, *267*, 128904.
(1069) Vavrova, S.; Struharnanska, E.; Turna, J.; Stuchlik, S. Tellurium: A Rare Element with Influence on Prokaryotic and Eukaryotic Biological Systems. *Int J Mol Sci* **2021**, *22* (11). DOI: 10.3390/ijms22115924.
(1070) Velzen, V.; D.; Langenkamp, H.; Herb., G. "Antimony, its sources, applications and flow paths into urban and industrial waste: a review.". *Waste management & research* **1998**, *16*.
(1071) Ventura-Lima, J.; Bogo, M. R.; Monserrat, J. M. Arsenic toxicity in mammals and aquatic animals: a comparative biochemical approach. *Ecotoxicol Environ Saf* **2011**, *74* (3), 211-218. DOI: 10.1016/j.ecoenv.2010.11.002.
(1072) Vesely, J.; Norton, S. A.; Skrivan, P.; Majer, V.; Kram, P.; Navratil, T.; Kaste, J. M. Environmental chemistry of beryllium. *Beryllium: Mineralogy, Petrology, and Geochemistry* **2002**, *50*, 291-317. DOI: 10.2138/rmg.2002.50.7.

(1073) Vij, D. R.; Singh, N. Thermoluminescence dosimetric properties of beryllium oxide. *Journal of Materials Science* **1997**, *32* (11), 2791-2796. DOI: 10.1023/A:1018608113663.
(1074) Villaescusa, I.; Bollinger, J.-C. Arsenic in drinking water: sources, occurrence and health effects (a review). *Reviews in Environmental Science and Bio/Technology* **2008**, *7*, 307-323.
(1075) Villar, L. d.; Javier, F. Natural fluid-deposited graphite: mineralogical characteristics and mechanisms of formation. *American journal of science* **1998**, *298*.
(1076) Vincent, J. B. The bioinorganic chemistry of chromium(III). *Polyhedron* **2001**, *20* (1-2), 1-26. DOI: 10.1016/S0277-5387(00)00624-0.
(1077) Vincent, J. B. Recent developments in the biochemistry of chromium(III). *Biol Trace Elem Res* **2004**, *99* (1-3), 1-16. DOI: 10.1385/BTER:99:1-3:001.
(1078) Vithanage, M.; Dabrowska, B. B.; Mukherjee, A. B.; Sandhi, A.; Bhattacharya, P. Arsenic uptake by plants and possible phytoremediation applications: a brief overview. *Environmental Chemistry Letters* **2012**, *10* (3), 217-224. DOI: 10.1007/s10311-011-0349-8.
(1079) Vodyanitskii, Y. N. Mineralogy and geochemistry of manganese: A review of publications. *Eurasian Soil Science* **2009**, *42* (10), 1170-1178. DOI: 10.1134/S1064229309100123.
(1080) Vodyanitskii, Y. N. Chemical Aspects of Uranium Behavior in Soils: A Review. *Eurasian Soil Science* **2011**, *44* (8), 862-873. DOI: 10.1134/S1064229311080163.
(1081) Vymazal, J. Occurrence and chemistry of zinc in freshwaters—its toxicity and bioaccumulation with respect to algae: a review. Part 1: Occurrence and chemistry of zinc in freshwaters. *Acta hydrochimica et hydrobiologica* **1985**, *13(6),* 627-654.
(1082) Vymazal, J. Occurrence and Chemistry of Zinc in Freshwaters–its Toxicity and Bioaccumulation with Respect to Algae: A Review Part 2: Toxicity and Bioaccumulation with Respect to Algae. *Acta hydrochimica et hydrobiologica* **1986**, *14(2),* 83-102.
(1083) Walkey, C.; Das, S.; Seal, S.; Erlichman, J.; Heckman, K.; Ghibelli, L.; Traversa, E.; McGinnis, J. F.; Self, W. T. Catalytic Properties and Biomedical Applications of Cerium Oxide Nanoparticles. *Environ Sci Nano* **2015**, *2* (1), 33-53. DOI: 10.1039/C4EN00138A.
(1084) Wall, J. D.; Krumholz, L. R. Uranium reduction. *Annu Rev Microbiol* **2006**, *60*, 149-166. DOI: 10.1146/annurev.micro.59.030804.121357.
(1085) Walsh, C. T.; Sandstead, H. H.; Prasad, A. S.; Newberne, P. M.; Fraker, P. J. Zinc: health effects and research priorities for the 1990s. *Environ Health Perspect* **1994**, *102 Suppl 2* (Suppl 2), 5-46. DOI: 10.1289/ehp.941025.
(1086) Wang, B. Recent development of non-platinum catalysts for oxygen reduction reaction. *Journal of Power Sources* **2005**, *152* (1), 1-15. DOI: 10.1016/j.jpowsour.2005.05.098.
(1087) Wang, C.; Xu, L.; Li, X.; Lin, Q. Divalent europium nanocrystals: controllable synthesis, properties, and applications. *Chemphyschem* **2012**, *13* (17), 3765-3772. DOI: 10.1002/cphc.201200163.
(1088) Wang, D. K.; Zhang, J. P.; Dong, Y.; Bin, C.; Li, A.; Chen, X. H.; Yang, R.; Song, H. H. Progress on graphitic carbon materials for potassium-based energy storage. *New Carbon Materials* **2021**, *36* (3), 435-446. DOI: 10.1016/S1872-5805(21)60039-2.
(1089) Wang, S.; Ran, Y.; Lu, B.; Li, J.; Kuang, H.; Gong, L.; Hao, Y. A Review of Uranium-Induced Reproductive Toxicity. *Biol Trace Elem Res* **2020**, *196* (1), 204-213. DOI: 10.1007/s12011-019-01920-2.
(1090) Wang, W. W.; Cheng, C. Y. Separation and purification of scandium by solvent extraction and related technologies: a review. *Journal of Chemical Technology and Biotechnology* **2011**, *86* (10), 1237-1246. DOI: 10.1002/jctb.2655.

(1091) Wang, W. W.; Pranolo, Y.; Cheng, C. Y. Metallurgical processes for scandium recovery from various resources: A review. *Hydrometallurgy* **2011**, *108* (1-2), 100-108. DOI: 10.1016/j.hydromet.2011.03.001.
(1092) Wang, X.; Wang, X.; Guo, Z. Functionalization of Platinum Complexes for Biomedical Applications. *Acc Chem Res* **2015**, *48* (9), 2622-2631. DOI: 10.1021/acs.accounts.5b00203.
(1093) Wang, Y.; Wang, S.; Xu, P. P.; Liu, C.; Liu, M. S.; Wang, Y. L.; Wang, C. H.; Zhang, C. H.; Ge, Y. Review of arsenic speciation, toxicity and metabolism in microalgae. *Reviews in Environmental Science and Bio-Technology* **2015**, *14* (3), 427-451. DOI: 10.1007/s11157-015-9371-9.
(1094) Wang, Y.; Yu, J. L.; Wang, Z. H.; Liu, Y. X.; Zhao, Y. C. A review on arsenic removal from coal combustion: Advances, challenges and opportunities. *Chemical Engineering Journal* **2021**, *414,* 128785. DOI: 10.1016/j.cej.2021.128785.
(1095) Wang, Y. X.; Li, J. X.; Ma, T.; Xie, X. J.; Deng, Y. M.; Gan, Y. Q. Genesis of geogenic contaminated groundwater: As, F and I. *Critical Reviews in Environmental Science and Technology* **2021**, *51* (24), 2895-2933. DOI: 10.1080/10643389.2020.1807452.
(1096) Wang, Z. C.; Li, M. Y. H.; Liu, Z. R. R.; Zhou, M. F. Scandium: Ore deposits, the pivotal role of magmatic enrichment and future exploration. *Ore Geology Reviews* **2021**, *128,* 103906. DOI: 10.1016/j.oregeorev.2020.103906.
(1097) Wang, Z. W.; Chen, M.; Huang, D. L.; Zeng, G. M.; Xu, P.; Zhou, C. Y.; Lai, C.; Wang, H.; Cheng, M.; Wang, W. J. Multiply structural optimized strategies for bismuth oxyhalide photocatalysis and their environmental application. *Chemical Engineering Journal* **2019**, *374,* 1025-1045. DOI: 10.1016/j.cej.2019.06.018.
(1098) Wang, Z. Y.; Fan, H. R.; Zhou, L. L.; Yang, K. F.; She, H. D. Carbonatite-Related REE Deposits: An Overview. *Minerals* **2020**, *10* (11), 965. DOI: 10.3390/min10110965.
(1099) Wani, A.; Shadab, G. G. H. A.; Afzal, M. Lead and zinc interactions - An influence of zinc over lead related toxic manifestations. *Journal of Trace Elements in Medicine and Biology* **2021**, *64,* 126702. DOI: 10.1016/j.jtemb.2020.126702.
(1100) Wanty, R. B.; Goldhaber, M. B. Thermodynamics and Kinetics of Reactions Involving Vanadium in Natural Systems - Accumulation of Vanadium in Sedimentary-Rocks. *Geochimica Et Cosmochimica Acta* **1992**, *56* (4), 1471-1483. DOI: 10.1016/0016-7037(92)90217-7.
(1101) Warheit, D. B.; Donner, E. M. Risk assessment strategies for nanoscale and fine-sized titanium dioxide particles: Recognizing hazard and exposure issues. *Food Chem Toxicol* **2015**, *85*, 138-147. DOI: 10.1016/j.fct.2015.07.001.
(1102) Waseem, A.; Ullah, H.; Rauf, M. K.; Ahmad, I. Distribution of Natural Uranium in Surface and Groundwater Resources: A Review. *Critical Reviews in Environmental Science and Technology* **2015**, *45* (22), 2391-2423. DOI: 10.1080/10643389.2015.1025642.
(1103) Wasel, O.; Freeman, J. L. Comparative Assessment of Tungsten Toxicity in the Absence or Presence of Other Metals. *Toxics* **2018**, *6* (4). DOI: 10.3390/toxics6040066.
(1104) Waters, L. S. Bacterial manganese sensing and homeostasis. *Curr Opin Chem Biol* **2020**, *55*, 96-102. DOI: 10.1016/j.cbpa.2020.01.003.
(1105) Watt, C.; Le, X. C. Arsenic speciation in natural waters. *Biogeochemistry of Environmentally Important Trace Elements* **2003**, *835*, 11-32.
(1106) Office of Research and Development, U.S. EPA. Rare earth elements: A review of production, processing, recycling, and associated environmental issues. *EPA/600/R-12/572,* **2012**.

(1107) Weckhuysen, B. M.; Wachs, I. E.; Schoonheydt, R. A. Surface Chemistry and Spectroscopy of Chromium in Inorganic Oxides. *Chem Rev* **1996**, *96* (8), 3327-3350. DOI: 10.1021/cr940044o.
(1108) Wegscheider, S.; Steinlechner, S.; Leuchtenmuller, M. Innovative Concept for the Recovery of Silver and Indium by a Combined Treatment of Jarosite and Electric Arc Furnace Dust. *Jom* **2017**, *69* (2), 388-394. DOI: 10.1007/s11837-016-2192-7.
(1109) Wei, Q.; Deng, H.; Cui, H.; Fang, J.; Zuo, Z.; Deng, J.; Li, Y.; Wang, X.; Zhao, L. A mini review of fluoride-induced apoptotic pathways. *Environ Sci Pollut Res Int* **2018**, *25* (34), 33926-33935. DOI: 10.1007/s11356-018-3406-z.
(1110) Wei, Y. N.; Han, R. R.; Xie, Y. H.; Jiang, C. D.; Yu, Y. X. Recent Advances in Understanding Mechanisms of Plant Tolerance and Response to Aluminum Toxicity. *Sustainability* **2021**, *13* (4), 1782. DOI: 10.3390/su13041782.
(1111) Weng, H., Z.; Jowitt, S. M.; Mudd, G. M.; Haque, N. Assessing rare earth element mineral deposit types and links to environmental impacts. *Applied Earth Science* **2013**, *122(2),* 83-96.
(1112) White, S. J. O.; Hemond, H. F. The anthrobiogeochemical cycle of indium: a review of the natural and anthropogenic cycling of indium in the environment. *Critical reviews in environmental science and technology* **2012**, *42,* 155-186. DOI: 10.1080/10643389.2010.498755
(1113) White, D. J.; Levy, L. S. Vanadium: environmental hazard or environmental opportunity? A perspective on some key research needs. *Environmental Science-Processes & Impacts* **2021**, *23* (4), 527-534. DOI: 10.1039/d0em00470g.
(1114) White, P. J.; Broadley, M. R. Mechanisms of caesium uptake by plants. *New Phytologist* **2000**, *147* (2), 241-256. DOI: 10.1046/j.1469-8137.2000.00704.x.
(1115) Wiche, O.; Szekely, B.; Moschner, C.; Heilmeier, H. Germanium in the soil-plant system-a review. *Environ Sci Pollut Res Int* **2018**, *25* (32), 31938-31956. DOI: 10.1007/s11356-018-3172-y.
(1116) Wilkins, M. J.; Livens, F. R.; Vaughan, D. J.; Lloyd, J. R. The impact of Fe(III)-reducing bacteria on uranium mobility. *Biogeochemistry* **2006**, *78* (2), 125-150. DOI: 10.1007/s10533-005-3655-z.
(1117) Willhite, C. C.; Ball, G. L.; McLellan, C. J. Total allowable concentrations of monomeric inorganic aluminum and hydrated aluminum silicates in drinking water. *Crit Rev Toxicol* **2012**, *42* (5), 358-442. DOI: 10.3109/10408444.2012.674101.
(1118) Williams-Jones, A. E.; Vasyukova, O. V. The Economic Geology of Scandium, the Runt of the Rare Earth Element Litter. *Economic Geology* **2018**, *113* (4), 973-988. DOI: 10.5382/econgeo.2018.4579.
(1119) Wilson, S. C.; Lockwood, P. V.; Ashley, P. M.; Tighe, M. The chemistry and behaviour of antimony in the soil environment with comparisons to arsenic: a critical review. *Environ Pollut* **2010**, *158* (5), 1169-1181. DOI: 10.1016/j.envpol.2009.10.045.
(1120) Winship, K. A. Toxicity of tin and its compounds. *Adverse Drug React Acute Poisoning Rev* **1988**, *7* (1), 19-38.
(1121) Winslow, K. M.; Laux, S. J.; Townsend, T. G. A review on the growing concern and potential management strategies of waste lithium-ion batteries. *Resour Conserv Recy* **2018**, *129*, 263-277. DOI: 10.1016/j.resconrec.2017.11.001.
(1122) Winter, B. L.; Johnson, C. M.; Clark, D. L. Strontium, neodymium, and lead isotope variations of authigenic and silicate sediment components from the Late Cenozoic Arctic Ocean:

Implications for sediment provenance and the source of trace metals in seawater. *Geochimica Et Cosmochimica Acta* **1997**, *61* (19), 4181-4200. DOI: 10.1016/S0016-7037(97)00215-9.
(1123) Wiseman, C. L.; Zereini, F. Airborne particulate matter, platinum group elements and human health: a review of recent evidence. *Sci Total Environ* **2009**, *407* (8), 2493-2500. DOI: 10.1016/j.scitotenv.2008.12.057.
(1124) Wood, S. A.; Mountain, B. W.; Pan, P. The aqueous geochemistry of platinum, palladium and gold; recent experimental constraints and a re-evaluation of theoretical predictions. *The Canadian Mineralogist* **1992**, *30(4)*, 955-982.
(1125) Wood, S. A. The Aqueous Geochemistry of the Rare-Earth Elements and Yttrium .1. Review of Available Low-Temperature Data for Inorganic Complexes and the Inorganic Ree Speciation of Natural-Waters. *Chemical Geology* **1990**, *82* (1-2), 159-186. DOI: 10.1016/0009-2541(90)90080-Q.
(1126) Wood, S. A.; Samson, I. M. The aqueous geochemistry of gallium, germanium, indium and scandium. *Ore Geology Reviews* **2006**, *28* (1), 57-102. DOI: 10.1016/j.oregeorev.2003.06.002.
(1127) Wu, J.; Yang, H. Platinum-based oxygen reduction electrocatalysts. *Acc Chem Res* **2013**, *46* (8), 1848-1857. DOI: 10.1021/ar300359w.
(1128) Wu, S. J.; Yan, P. J.; Yu, W. S.; Cheng, K.; Wang, H.; Yang, W.; Zhou, J.; Xi, J. H.; Qiu, J. S.; Zhu, S. X.; Che, L. Efficient removal of mercury from flue gases by regenerable cerium-doped functional activated carbon derived from resin made by in situ ion exchange method. *Fuel Processing Technology* **2019**, *196,* 106167. DOI: 10.1016/j.fuproc.2019.106167.
(1129) Wu, Y.; Kong, L. Advance on toxicity of metal nickel nanoparticles. *Environ Geochem Health* **2020**, *42* (7), 2277-2286. DOI: 10.1007/s10653-019-00491-4.
(1130) Xiao, Z.; Laplante, A. R. Characterizing and recovering the platinum group minerals - a review. *Minerals Engineering* **2004**, *17* (9-10), 961-979. DOI: 10.1016/j.mineng.2004.04.001.
(1131) Xie, K.; Cakmak, I.; Wang, S.; Zhang, F.; Guo, S. Synergistic and antagonistic interactions between potassium and magnesium in higher plants. *Crop J* **2021**, *9(2)*, 249-256.
(1132) Xing, P.; Wang, C. Y.; Chen, Y. Q.; Ma, B. Z. Rubidium extraction from mineral and brine resources: A review. *Hydrometallurgy* **2021**, *203,* 105644. DOI: 10.1016/j.hydromet.2021.105644.
(1133) Xu, C.; Zhang, Y.; Zhang, N.; Liu, X.; Yi, J.; Liu, X.; Lu, X.; Ru, Q.; Lu, H.; Peng, X.; Zhao, X. S.; Ma, J. 2020 Roadmap on Zinc Metal Batteries. *Chem Asian J* **2020**, *15* (22), 3696-3708. DOI: 10.1002/asia.202000946.
(1134) Xu, W.; Wang, Y. Recent Progress on Zinc-Ion Rechargeable Batteries. *Nanomicro Lett* **2019**, *11* (1), 90. DOI: 10.1007/s40820-019-0322-9.
(1135) Xu, X. Y.; Lin, Y. J.; Evans, D. G.; Duan, X. Layered intercalated functional materials based on efficient utilization of magnesium resources in China. *Science China-Chemistry* **2010**, *53* (7), 1461-1469. DOI: 10.1007/s11426-010-4031-y.
(1136) Yamamoto, Y. Aluminum toxicity in plant cells: Mechanisms of cell death and inhibition of cell elongation. *Soil Science and Plant Nutrition* **2019**, *65* (1), 41-55. DOI: 10.1080/00380768.2018.1553484.
(1137) Yan, L.; Riaz, M.; Liu, J.; Yu, M.; Cuncang, J. The aluminum tolerance and detoxification mechanisms in plants; recent advances and prospects. *Critical Reviews in Environmental Science and Technology* **2021**, *52(9)*, 1491-1527.

(1138) Yan, L.; Rui, X.; Chen, G.; Xu, W.; Zou, G.; Luo, H. Recent advances in nanostructured Nb-based oxides for electrochemical energy storage. *Nanoscale* **2016**, *8* (16), 8443-8465. DOI: 10.1039/c6nr01340f.
(1139) Yang, W.-F.; Igbari, F.; Lou, Y.-H.; Wang, Z.-K.; Liao, L.-S. Tin halide perovskites: progress and challenges. *Advanced Energy Materials* **2020**, *10*.
(1140) Yang, D. J.; Zhang, L. J.; Yan, X. C.; Yao, X. D. Recent Progress in Oxygen Electrocatalysts for Zinc-Air Batteries. *Small Methods* **2017**, *1* (12), 1700209. DOI: 10.1002/smtd.201770123.
(1141) Yang, J. L.; Fan, W.; Zheng, S. J. Mechanisms and regulation of aluminum-induced secretion of organic acid anions from plant roots. *J Zhejiang Univ Sci B* **2019**, *20* (6), 513-527. DOI: 10.1631/jzus.B1900188.
(1142) Yang, J. X.; Retegan, T.; Steenari, B. M.; Ekberg, C. Recovery of indium and yttrium from Flat Panel Display waste using solvent extraction. *Separation and Purification Technology* **2016**, *166*, 117-124. DOI: 10.1016/j.seppur.2016.04.021.
(1143) Yang, N.; Sun, H. Biocoordination chemistry of bismuth: Recent advances. *Coordination Chemistry Reviews* **2007**, *251* (17-20), 2354-2366. DOI: 10.1016/j.ccr.2007.03.003.
(1144) Yang, N.; Tang, S. H.; Zhang, S. H.; Huang, W. H.; Chen, P.; Chen, Y. Y.; Xi, Z. D.; Yuan, Y.; Wang, K. F. Fluorine in Chinese Coal: A Review of Distribution, Abundance, Modes of Occurrence, Genetic Factors and Environmental Effects. *Minerals* **2017**, *7* (11), 219. DOI: 10.3390/min7110219.
(1145) Yang, S.; Ellis, A. M. Helium droplets: a chemistry perspective. *Chem Soc Rev* **2013**, *42* (2), 472-484. DOI: 10.1039/c2cs35277j.
(1146) Yang, X. S. Beneficiation studies of tungsten ores - A review. *Minerals Engineering* **2018**, *125*, 111-119. DOI: 10.1016/j.mineng.2018.06.001.
(1147) Yao, H.; Zhou, F.; Li, Z.; Ci, Z.; Ding, L.; Jin, Z. Strategies for Improving the Stability of Tin-Based Perovskite (ASnX(3)) Solar Cells. *Adv Sci (Weinh)* **2020**, *7* (10), 1903540. DOI: 10.1002/advs.201903540.
(1148) Yao, L.; Yang, H.; Chen, Z. S.; Qiu, M. Q.; Hu, B. W.; Wang, X. X. Bismuth oxychloride-based materials for the removal of organic pollutants in wastewater. *Chemosphere* **2021**, *273*, 128576. DOI: 10.1016/j.chemosphere.2020.128576.
(1149) Yaqoob, S. B.; Adnan, R.; Rameez Khan, R. M.; Rashid, M. Gold, Silver, and Palladium Nanoparticles: A Chemical Tool for Biomedical Applications. *Front Chem* **2020**, *8*, 376. DOI: 10.3389/fchem.2020.00376.
(1150) Ye, Q.; Park, J. E.; Gugnani, K.; Betharia, S.; Pino-Figueroa, A.; Kim, J. Influence of iron metabolism on manganese transport and toxicity. *Metallomics* **2017**, *9* (8), 1028-1046. DOI: 10.1039/c7mt00079k.
(1151) Ye, Y. Q.; Medina-Velo, I. A.; Cota-Ruiz, K.; Moreno-Olivas, F.; Gardea-Torresdey, J. L. Can abiotic stresses in plants be alleviated by manganese nanoparticles or compounds? *Ecotoxicology and Environmental Safety* **2019**, *184*, 109671. DOI: 10.1016/j.ecoenv.2019.109671.
(1152) Yi, Y.; Zhi-Dong, A.; Xiao-Yi, C.; Ming-Lei, G.; Cheng-Bin, J.; Yan-Qing, L. Recent progress of tin-based perovskites and their applications in light-emitting diodes. *Acta Physica Sinica* **2021**, *70(4)*.
(1153) Yi, H.; Qin, L.; Huang, D. L.; Zeng, G. M.; Lai, C.; Liu, X. G.; Li, B. S.; Wang, H.; Zhou, C. Y.; Huang, F. L.; Liu, S. Y.; Guo, X. Y. Nano-structured bismuth tungstate with

controlled morphology: Fabrication, modification, environmental application and mechanism insight. *Chemical Engineering Journal* **2019**, *358*, 480-496. DOI: 10.1016/j.cej.2018.10.036.
(1154) Yi, J.; Liang, P. C.; Liu, X. Y.; Wu, K.; Liu, Y. Y.; Wang, Y. G.; Xia, Y. Y.; Zhang, J. J. Challenges, mitigation strategies and perspectives in development of zinc-electrode materials and fabrication for rechargeable zinc-air batteries. *Energy & Environmental Science* **2018**, *11* (11), 3075-3095. DOI: 10.1039/c8ee01991f.
(1155) Yin, X. B.; Martineau, C.; Demers, I.; Basiliko, N.; Fenton, N. J. The potential environmental risks associated with the development of rare earth element production in Canada. *Environmental Reviews* **2021**, *29* (3), 354-377. DOI: 10.1139/er-2020-0115.
(1156) Yokel, R. A. Aluminum chelation principles and recent advances. *Coordination Chemistry Reviews* **2002**, *228* (2), 97-113. DOI: 10.1016/S0010-8545(02)00078-4.
(1157) Yokel, R. A. Aluminum reproductive toxicity: a summary and interpretation of scientific reports. *Crit Rev Toxicol* **2020**, *50* (7), 551-593. DOI: 10.1080/10408444.2020.1801575.
(1158) You, G.; Hou, J.; Xu, Y.; Miao, L.; Ao, Y.; Xing, B. Surface Properties and Environmental Transformations Controlling the Bioaccumulation and Toxicity of Cerium Oxide Nanoparticles: A Critical Review. *Reviews of Environmental Contamination and Toxicology Volume 253,* **2020**.
(1159) Young, E. D.; Galy, A. The isotope geochemistry and cosmochemistry of magnesium. *Geochemistry of Non-Traditional Stable Isotopes* **2004**, *55*, 197-230. DOI: 10.2138/gsrmg.55.1.197.
(1160) Young, R. S. The Geochemistry of Cobalt. *Geochimica Et Cosmochimica Acta* **1957**, *13* (1), 28-41. DOI: Doi 10.1016/0016-7037(57)90056-X.
(1161) Yu; Hai-tao. Zinc as a countermeasure for cadmium toxicity. *Acta Pharmacologica Sinica* **2021**, *42*.
(1162) Yu, X.; Liu, C.; Guo, Y.; Deng, T. Speciation Analysis of Trace Arsenic, Mercury, Selenium and Antimony in Environmental and Biological Samples Based on Hyphenated Techniques. *Molecules* **2019**, *24* (5). DOI: 10.3390/molecules24050926.
(1163) Yu, X. W.; Ye, S. Y. Recent advances in activity and durability enhancement of Pt/C catalytic cathode in PEMFC - Part II: Degradation mechanism and durability enhancement of carbon supported platinum catalyst. *Journal of Power Sources* **2007**, *172* (1), 145-154. DOI: 10.1016/j.jpowsour.2007.07.048.
(1164) Yu, Y.; Yu, L.; Koh, K. Y.; Wang, C. H.; Chen, J. P. Rare-earth metal based adsorbents for effective removal of arsenic from water: A critical review. *Critical Reviews in Environmental Science and Technology* **2018**, *48* (22-24), 1127-1164. DOI: 10.1080/10643389.2018.1514930.
(1165) Yu, Z.; Chan, W. K.; Tan, T. T. Y. Neodymium-Sensitized Nanoconstructs for Near-Infrared Enabled Photomedicine. *Small* **2020**, *16* (1), e1905265. DOI: 10.1002/smll.201905265.
(1166) Yuan, W.; Chen, J. B.; Teng, H.; Chetelat, B.; Cai, H. M.; Liu, J. C.; Wang, Z. C.; Bouchez, J.; Moynier, F.; Gaillardet, J.; Schott, J.; Liu, C. Q. A Review on the Elemental and Isotopic Geochemistry of Gallium. *Global Biogeochemical Cycles* **2021**, *35* (9), e2021GB007033. DOI: 10.1029/2021GB007033.
(1167) Yudovich, Y. E. Notes on the marginal enrichment of Germanium in coal beds. *International Journal of Coal Geology* **2003**, *56* (3-4), 223-232. DOI: 10.1016/j.coal.2003.08.003.
(1168) Liu, Y.; Wu, Y.; Pang, H.; Wang, X.; Yu, S.; Wang, X. Study on the removal of water pollutants by graphite phase carbon nitride materials. *Progress in Chemistry* **2019**, *31(6)*, 831.

(1169) Yue, Y. C.; Li, M. H.; Wang, H. B.; Zhang, B. L.; He, W. The toxicological mechanisms and detoxification of depleted uranium exposure. *Environ Health Prev Med* **2018**, *23* (1), 18. DOI: 10.1186/s12199-018-0706-3.
(1170) Zachariadis, G. A.; Vogiatzis, C. An Overview of the Use of Yttrium for Internal Standardization in Inductively Coupled Plasma-Atomic Emission Spectrometry. *Applied Spectroscopy Reviews* **2010**, *45* (3), 220-239. DOI: 10.1080/05704921003719122.
(1171) Zare, B.; Nami, M.; Shahverdi, A. R. Tracing Tellurium and Its Nanostructures in Biology. *Biol Trace Elem Res* **2017**, *180* (2), 171-181. DOI: 10.1007/s12011-017-1006-2.
(1172) Zayed, A. M.; Terry, N. Chromium in the environment: factors affecting biological remediation. *Plant and Soil* **2003**, *249* (1), 139-156. DOI: 10.1023/A:1022504826342.
(1173) Zeimentz, P. M.; Arndt, S.; Elvidge, B. R.; Okuda, J. Cationic organometallic complexes of scandium, yttrium, and the lanthanoids. *Chem Rev* **2006**, *106* (6), 2404-2433. DOI: 10.1021/cr050574s.
(1174) Zeman, T.; Loh, E. W.; Cierny, D.; Sery, O. Penetration, distribution and brain toxicity of titanium nanoparticles in rodents' body: a review. *IET Nanobiotechnol* **2018**, *12* (6), 695-700. DOI: 10.1049/iet-nbt.2017.0109.
(1175) Zeng; Kai; Xue, D.-J.; Tang., J. Antimony selenide thin-film solar cells. *Semiconductor Science and Technology* **2016**, *31*.
(1176) Zhai, J. H.; Chen, P.; Sun, W.; Chen, W.; Wan, S. A review of mineral processing of ilmenite by flotation. *Minerals Engineering* **2020**, *157*, 106558. DOI: 10.1016/j.mineng.2020.106558.
(1177) Zhang, X. J.; Lai, T. B.; Kong, R.Y.-C. Biology of Fluoro-Organic Compounds. In: *Fluorous Chemistry. Topics in Current Chemistry vol. 308;* Horváth, I. (Ed.); Springer, Berlin, Heidelberg **2011**. DOI: 10.1007/128_2011_270.
(1178) Zhang Y.; Sun, W.; Xu, R.; Wang, L.; Tang, H. Lithium extraction from water lithium resources through green electrochemical-battery approaches: A comprehensive review. *Journal of Cleaner Production* **2021**, *285I, 124905*.
(1179) Zhang; Z.; Zhang, J.-H.; Wang, J.; Li, Z.-H.; Xie, J.-S.; Liu, S.-J.; Guan, K.; Wu, R.-Z. Toward the development of Mg alloys with simultaneously improved strength and ductility by refining grain size via the deformation process. *International Journal of Minerals, Metallurgy and Materials* **2021**, *28*, 30-45.
(1180) Zhang, J. F.; Zhou, Y.; Yoon, J.; Kim, J. S. Recent progress in fluorescent and colorimetric chemosensors for detection of precious metal ions (silver, gold and platinum ions). *Chem Soc Rev* **2011**, *40* (7), 3416-3429. DOI: 10.1039/c1cs15028f.
(1181) Zhang, J. J.; Wang, H.; Yuan, X. Z.; Zeng, G. M.; Tu, W. G.; Wang, S. B. Tailored indium sulfide-based materials for solar-energy conversion and utilization. *Journal of Photochemistry and Photobiology C-Photochemistry Reviews* **2019**, *38*, 1-26. DOI: 10.1016/j.jphotochemrev.2018.11.001.
(1182) Zhang, K.; Kleit, A. N.; Nieto, A. An economics strategy for criticality - Application to rare earth element Yttrium in new lighting technology and its sustainable availability. *Renewable & Sustainable Energy Reviews* **2017**, *77*, 899-915. DOI: 10.1016/j.rser.2016.12.127.
(1183) Zhang, K. H.; Wu, Y. F.; Wang, W.; Li, B.; Zhang, Y. N.; Zuo, T. Y. Recycling indium from waste LCDs: A review. *Resour Conserv Recy* **2015**, *104*, 276-290. DOI: 10.1016/j.resconrec.2015.07.015.
(1184) Zhang, N.; Li, H. X.; Liu, X. M. Recovery of scandium from bauxite residue-red mud: a review. *Rare Metals* **2016**, *35* (12), 887-900. DOI: 10.1007/s12598-016-0805-5.

(1185) Zhang, N. N.; Nguyen, A. V.; Zhou, C. C. A review of the surface features and properties, surfactant adsorption and floatability of four key minerals of diasporic bauxite resources. *Advances in Colloid and Interface Science* **2018**, *254*, 56-75. DOI: 10.1016/j.cis.2018.03.005.
(1186) Zhang, S.; Li, P.; Li, Z. H. Toxicity of organotin compounds and the ecological risk of organic tin with co-existing contaminants in aquatic organisms. *Comp Biochem Physiol C Toxicol Pharmacol* **2021**, *246*, 109054. DOI: 10.1016/j.cbpc.2021.109054.
(1187) Zhang, X.; Li, W.; Yang, Z. Toxicology of nanosized titanium dioxide: an update. *Arch Toxicol* **2015**, *89* (12), 2207-2217. DOI: 10.1007/s00204-015-1594-6.
(1188) Zhang, X.; Long, Y.; Huang, J.; Xia, J. Molecular Mechanisms for Coping with Al Toxicity in Plants. *Int J Mol Sci* **2019**, *20* (7). DOI: 10.3390/ijms20071551.
(1189) Zhang, X.; Ma, G. J.; Liu, M. K.; Li, Z. Removal of Residual Element Tin in the Ferrous Metallurgy Process: A Review. *Metals* **2019**, *9* (8), 834. DOI: 10.3390/met9080834.
(1190) Zhao, F. J.; McGrath, S. P.; Meharg, A. A. Arsenic as a Food Chain Contaminant: Mechanisms of Plant Uptake and Metabolism and Mitigation Strategies. *Annual Review of Plant Biology, Vol 61* **2010**, *61*, 535-559. DOI: 10.1146/annurev-arplant-042809-112152.
(1191) Zhao, J.; Shi, X.; Castranova, V.; Ding, M. Occupational toxicology of nickel and nickel compounds. *J Environ Pathol Toxicol Oncol* **2009**, *28* (3), 177-208. DOI: 10.1615/jenvironpatholtoxicoloncol.v28.i3.10.
(1192) Zhao, X. H.; Cheruvally, G.; Kim, C.; Cho, K. K.; Ahn, H. J.; Kim, K. W.; Ahn, J. H. Lithium/Sulfur Secondary Batteries: A Review. *Journal of Electrochemical Science and Technology* **2016**, *7* (2), 97-114. DOI: 10.5229/Jecst.2016.7.2.97.
(1193) Zhao, X. Q.; Shen, R. F. Aluminum-Nitrogen Interactions in the Soil-Plant System. *Front Plant Sci* **2018**, *9*, 807. DOI: 10.3389/fpls.2018.00807.
(1194) Zhao, X. Y.; Yang, H. C.; Wang, Y. F.; Sha, Z. L. Review on the electrochemical extraction of lithium from seawater/brine. *Journal of Electroanalytical Chemistry* **2019**, *850*, 113389. DOI: 10.1016/j.jelechem.2019.113389.
(1195) Zheng; Jiangfu. A review of public and environmental consequences of organic germanium. *Critical Reviews in Environmental Science and Technology* **2020**, *50*.
(1196) Zheng, H. D.; Ding, Y. J.; Wen, Q.; Liu, B.; Zhang, S. G. Separation and purification of platinum group metals from aqueous solution: Recent developments and industrial applications. *Resour Conserv Recy* **2021**, *167*, 105417. DOI: 10.1016/j.resconrec.2021.105417.
(1197) Zheng, L.; Lan, P.; Shen, R. F.; Li, W. F. Proteomics of aluminum tolerance in plants. *Proteomics* **2014**, *14* (4-5), 566-578. DOI: 10.1002/pmic.201300252.
(1198) Zheng, R. L.; Sun, G. X.; Zhu, Y. G. Effects of microbial processes on the fate of arsenic in paddy soil. *Chinese Science Bulletin* **2013**, *58* (2), 186-193. DOI: 10.1007/s11434-012-5489-0.
(1199) Zheng, S. J.; Yang, J. L. Target sites of aluminum phytotoxicity. *Biologia Plantarum* **2005**, *49* (3), 321-331. DOI: DOI 10.1007/s10535-005-0001-1.
(1200) Zheng, W.; Fu, S. X.; Dydak, U.; Cowan, D. M. Biomarkers of manganese intoxication. *Neurotoxicology* **2011**, *32* (1), 1-8. DOI: 10.1016/j.neuro.2010.10.002.
(1201) Zhenghui, C.; Jifu, S.; Lijun, L.; Denghong, W.; Ting, L.; Chenghui, W. A preliminary review of metallogenic regularity of tin deposits in China. *Acta Geologica Sinica (English Edition)* **2015**, *89*, 1021-1035.
(1202) Zhi, Y.; Zhang, C.; Hjorth, R.; Baun, A.; Duckworth, O. W.; Call, D. F.; Knappe, D. R. U.; Jones, J. L.; Grieger, K. Emerging lanthanum (III)-containing materials for phosphate

removal from water: A review towards future developments. *Environ Int* **2020**, *145*, 106115. DOI: 10.1016/j.envint.2020.106115.
(1203) Zhou, B. L.; Li, Z. X.; Chen, C. C. Global Potential of Rare Earth Resources and Rare Earth Demand from Clean Technologies. *Minerals* **2017**, *7* (11), 203. DOI: 10.3390/min7110203.
(1204) Zhou, H.; Fu, C. Manganese-oxidizing microbes and biogenic manganese oxides: characterization, Mn(II) oxidation mechanism and environmental relevance. *Reviews in Environmental Science and Bio-Technology* **2020**, *19* (3), 489-507. DOI: 10.1007/s11157-020-09541-1.
(1205) Zhou, L. B.; Tan, Y. H.; Huang, L. M.; Fortin, C.; Campbell, P. G. C. Aluminum effects on marine phytoplankton: implications for a revised Iron Hypothesis (Iron-Aluminum Hypothesis). *Biogeochemistry* **2018**, *139* (2), 123-137. DOI: 10.1007/s10533-018-0458-6.
(1206) Zhu, S. S.; Ho, S. H.; Jin, C.; Duan, X. G.; Wang, S. B. Nanostructured manganese oxides: natural/artificial formation and their induced catalysis for wastewater remediation. *Environmental Science-Nano* **2020**, *7* (2), 368-396. DOI: 10.1039/c9en01250h.
(1207) Zhu, Y.; Li, Y.; Miao, L.; Wang, Y.; Liu, Y.; Yan, X.; Cui, X.; Li, H. Immunotoxicity of aluminum. *Chemosphere* **2014**, *104*, 1-6. DOI: 10.1016/j.chemosphere.2013.10.052.
(1208) Zhu, Y.; Yang, J.; Wang, L.; Lin, Z.; Dai, J.; Wang, R.; Yu, Y.; Liu, H.; Rensing, C.; Feng, R. Factors influencing the uptake and speciation transformation of antimony in the soil-plant system, and the redistribution and toxicity of antimony in plants. *Sci Total Environ* **2020**, *738*, 140232. DOI: 10.1016/j.scitotenv.2020.140232.
(1209) Zhu, Y. G.; Yoshinaga, M.; Zhao, F. J.; Rosen, B. P. Earth Abides Arsenic Biotransformations. *Annu Rev Earth Planet Sci* **2014**, *42*, 443-467. DOI: 10.1146/annurev-earth-060313-054942.
(1210) Zimmermann, S.; Sures, B. Significance of platinum group metals emitted from automobile exhaust gas converters for the biosphere. *Environ Sci Pollut Res Int* **2004**, *11* (3), 194-199. DOI: 10.1007/BF02979675.
(1211) Zinner, E.; Amari, S.; Wopenka, B.; Lewis, R. S. Interstellar Graphite in Meteorites - Isotopic Compositions and Structural-Properties of Single Graphite Grains from Murchison. *Meteoritics* **1995**, *30* (2), 209-226. DOI: DOI 10.1111/j.1945-5100.1995.tb01115.x.
(1212) Ziwa, G.; Crane, R.; Hudson-Edwards, K. A. Geochemistry, Mineralogy and Microbiology of Cobalt in Mining-Affected Environments. *Minerals* **2021**, *11* (1), 22. DOI: 10.3390/min11010022.
(1213) Zoroddu, M. A.; Medici, S.; Peana, M.; Nurchi, V. M.; Lachowicz, J. I.; Laulicht-Glickc, F.; Costa, M. Tungsten or Wolfram: Friend or Foe? *Curr Med Chem* **2018**, *25* (1), 65-74. DOI: 10.2174/0929867324666170428105603.
(1214) Zou, H.; Zhou, C.; Li, Y.; Yang, X.; Wen, J.; Hu, X.; Sun, C. Occurrence, toxicity, and speciation analysis of arsenic in edible mushrooms. *Food Chem* **2019**, *281*, 269-284. DOI: 10.1016/j.foodchem.2018.12.103.
(1215) Zubair, M.; Martyniuk., C. J. A review on hemato-biochemical, accumulation and patho-morphological responses of arsenic toxicity in ruminants. *Toxin Reviews* **2018**, *38(3)*, 176-186.
(1216) Zwolak, I. Vanadium carcinogenic, immunotoxic and neurotoxic effects: a review of in vitro studies. *Toxicol Mech Methods* **2014**, *24* (1), 1-12. DOI: 10.3109/15376516.2013.843110.
(1217) Zwolak, I. The Role of Selenium in Arsenic and Cadmium Toxicity: an Updated Review of Scientific Literature. *Biol Trace Elem Res* **2020**, *193* (1), 44-63. DOI: 10.1007/s12011-019-01691-w.
